# Phylogenomic analysis sheds light on the evolutionary pathways towards acoustic communication in Orthoptera

**DOI:** 10.1038/s41467-020-18739-4

**Published:** 2020-10-02

**Authors:** Hojun Song, Olivier Béthoux, Seunggwan Shin, Alexander Donath, Harald Letsch, Shanlin Liu, Duane D. McKenna, Guanliang Meng, Bernhard Misof, Lars Podsiadlowski, Xin Zhou, Benjamin Wipfler, Sabrina Simon

**Affiliations:** 1 Department of Entomology, Texas A&M University, College Station, TX 77843-2475 USA; 2 CR2P (Centre de Recherche en Paléontologie – Paris), MNHN – CNRS – Sorbonne Université, Muséum National d’Histoire Naturelle, 75005 Paris, France; 3 Department of Biological Sciences and Center for Biodiversity Research, University of Memphis, Memphis, TN 38152 USA; 4 School of Biological Sciences, Seoul National University, Seoul, 08826 Republic of Korea; 5 Center for Molecular Biodiversity Research (ZMB), Zoological Research Museum Alexander Koenig (ZFMK), 53113 Bonn, Germany; 6 Department für Botanik und Biodiversitätsforschung, Universität Wien, 1030 Vienna, Austria; 7 China National GeneBank, BGI-Shenzhen, 518083 Guangdong, China; 8 Department of Entomology, College of Plant Protection, China Agricultural University, 100193 Beijing, China; 9 Institut für Spezielle Zoologie und Evolutionsbiologie, Friedrich-Schiller-University Jena, 07743 Jena, Germany; 10 Center of Taxonomy and Evolutionary Research, Zoological Research Museum Alexander Koenig, 53113 Bonn, Germany; 11 Biosystematics Group, Wageningen University and Research, 6708 PB Wageningen, Netherlands

**Keywords:** Phylogenetics, Sexual selection, Animal behaviour, Entomology

## Abstract

Acoustic communication is enabled by the evolution of specialised hearing and sound producing organs. In this study, we performed a large-scale macroevolutionary study to understand how both hearing and sound production evolved and affected diversification in the insect order Orthoptera, which includes many familiar singing insects, such as crickets, katydids, and grasshoppers. Using phylogenomic data, we firmly establish phylogenetic relationships among the major lineages and divergence time estimates within Orthoptera, as well as the lineage-specific and dynamic patterns of evolution for hearing and sound producing organs. In the suborder Ensifera, we infer that forewing-based stridulation and tibial tympanal ears co-evolved, but in the suborder Caelifera, abdominal tympanal ears first evolved in a non-sexual context, and later co-opted for sexual signalling when sound producing organs evolved. However, we find little evidence that the evolution of hearing and sound producing organs increased diversification rates in those lineages with known acoustic communication.

## Abstract (web summary)

Song et al. inferred that stridulatory wings and tibial ears co-evolved in a sexual context among crickets, katydids, and their allies, while abdominal ears evolved first in a non-sexual context in grasshoppers, and were later co-opted for courtship. They found little evidence that the evolution of these organs increased lineage diversification.

## Introduction

Acoustic communication is one of the most conspicuous modes of signalling among animals. The use of acoustic signalling has been well documented in bony fishes, frogs, birds, cetaceans, terrestrial mammals and insects. Moreover, the intricate interplay and co-evolution between signal sender and receiver in the context of mating, prey location, predator avoidance and other interactions has led to the amazing diversity and complexity of the soundscape we know today^1–4^.

Signal emission and reception are the two major components of acoustic communication, and are enabled by dedicated sound-producing organs and matching hearing sensory organs. Across the animal kingdom, different vertebrate and ‘invertebrate’ lineages have independently evolved diverse structures and mechanisms for hearing and sound production^4–7^. For example, although all inner ear structures of vertebrates can be traced to the same structure found in Silurian ostracoderms^8^, tympanal ears have evolved independently in frogs, mammals, and reptiles^6^. As for the sound-producing organs, vocal cords in the larynx have evolved several times within tetrapods^6^, while birds have evolved a unique organ called the syrinx^9^. As for insects, the ability to hear using tympanal ears has independently evolved at least in seven different orders (Orthoptera, Mantodea, Hemiptera, Neuroptera, Coleoptera, Lepidoptera and Diptera), involving at least 15 body locations^10–13^. Although the lack of tympanal ears does not necessarily mean that other insect orders cannot hear, as it has been shown that internal sensory organs can be sensitive to sound without external tympana^14,15^, the tympanal ears clearly enable far-field hearing over a broad frequency range and at high sensitivity^14^. The ability to produce sound that can travel over a long distance using specialised organs, such as stridulatory (vibration-producing) apparatus or tymbals, has evolved at least in six insect orders (Blattodea, Coleoptera, Hemiptera, Lepidoptera, Mantodea and Orthoptera), also involving many body parts^6,11,16^.

While many studies have focused on the proximate mechanisms of hearing and sound production and the evolutionary processes driving the diversity of acoustic signalling^1–3,10,17–20^, questions about when, how, and in what context hearing and sound-producing organs evolved in the first place, and how these organs have co-evolved along the phylogeny remain inadequately addressed^7,11,12^. For insects that use acoustic signalling, there are at least two prevailing views on how these structures might have evolved originally^11,12^. The first view is that they could have evolved as an adaptation to detect and escape vertebrate predators^7,11,12,21–23^. Tympanal hearing may have evolved in the context of general auditory surveillance of the environment for predator movements, as it has been demonstrated in moths^24,25^, mantises^26^, and grasshoppers^27^. Likewise, early forms of stridulatory organs could have evolved as a defensive mechanism^6^, as part of a deimatic behaviour. These hearing and sound-producing organs could have also led to the evolution of sexual signalling via the so-called ‘sensory bias’ mechanism, in which male sexual signals evolve from structures originally involved in a non-sexual context that females already have perception for, also in a non-sexual context^11^. A phylogenetic pattern consistent with this sensory bias mechanism would be that, in a given lineage, the evolution of one component (e.g. hearing organ) would precede the evolution of its counterpart (e.g. sound-producing organ). The second view is that hearing and sound-producing organs could have evolved jointly as female perception and male signalling devices, co-evolved via a Fisherian mechanism^11^. It has been suggested that cicadas, crickets, and katydids evolved acoustic communication in this way^7,11^. A predictable phylogenetic pattern would be that the origin of both hearing and sound-producing organs would be traced to a single common ancestor. Thus, in order to gain deeper understanding of the evolution of acoustic communication, it is important to trace the evolution of hearing and sound-producing organs in a phylogenetic framework and in a lineage including both species lacking the ability to hear or produce sound and species with diverse acoustic communication strategies.

Among animal groups that exhibit acoustic communication, the insect order Orthoptera (crickets, katydids, grasshoppers and allies) stands out as an ideal model to address these evolutionary questions^7,11^. With about 16,000 species primarily using acoustic signalling as a main mode of sexual communication, it is the most species-rich clade of all acoustically communicating animals, outnumbering frogs, birds, mammals^28^ or any of the known acoustically-active insect lineages. Furthermore, within Orthoptera, there are lineages that do not use acoustic signalling for mating but for defensive signalling^29,30^, and others lacking specialised structures for hearing or sound production^11^. Orthoptera is also the earliest known lineage of animals to have evolved complex acoustic communication, as evidenced by fossil forewings possessing a stridulatory apparatus homologous to that of present-day crickets, which is known from as early as the Triassic^31,32^. Therefore, Orthoptera is an excellent group to study the evolution of acoustic communication, but the lack of robust, time-calibrated phylogeny has been a major challenge for inferring the complex and dynamic patterns of how hearing and sound-producing organs originated and evolved over time.

In this study, we reconstruct the evolution of hearing and sound-producing organs in Orthoptera, which can provide a bird’s-eye view of how acoustic communication originated and diversified during several hundred million years of evolution. We first establish reliable phylogenetic relationships among major lineages within Orthoptera by combining 4986 multiple sequence alignments of protein-coding genes selected from the transcriptomes of 60 taxa (50 orthopterans and 10 polyneopteran outgroups) and 249 previously and newly generated mitochondrial genomes (mtgenomes). We employ carefully selected fossils and rigorous topology testing to produce a robust, time-calibrated phylogeny of the order. This framework is then used to trace the evolution of tympanal ears and associated internal sensory organs, as well as that of diverse sound-producing mechanisms known in Orthoptera. This allows us to test evolutionary hypotheses regarding the origins of these organs and whether diversification patterns were influenced by these innovations. We find lineage-specific and dynamic patterns of evolution for hearing and sound-producing organs. Specifically, we infer that these two organs co-evolved in a sexual context in crickets, katydids and their allies, but we find little evidence that the evolution of these organs increased diversification rates in the singing lineages. Contrastingly, we find that the hearing organs evolved first in a non-sexual context in grasshoppers, and later co-opted for sexual signalling when sound-producing organs evolved.

## Results

### Phylogenetic relationships and divergence times of major orthopteran lineages

We thoroughly explored the signal in the phylogenomic data by creating and analysing six phylogenomic data sets differing in the level of matrix saturation, character coding (amino acid vs. nucleotide), and data size (nuclear genes only vs. combined) in a maximum likelihood framework (see Supplementary Methods 1.1-1.6). The six data sets resulted in largely congruent topologies in terms of family-level relationships (see Supplementary Fig. 3), but the phylogenetic placements of Rhaphidophoridae, Gryllotalpidae and Pamphagidae varied among the resulting trees. We applied four-cluster likelihood mapping (FcLM)^33^ and permutation tests for these specific relationships using all six data sets to check for confounding signal, such as among-lineage heterogeneity that violates globally stationary, reversible and homogeneous conditions, non-random distribution of missing data, and a mixture of both (Supplementary Methods 1.7). We found that the placement of Rhaphidophoridae was robust and unbiased, but the placements of Pamphagidae and Gryllotalpidae were potentially biased by the confounding signal and our small taxon sampling for these families was not sufficient to make unambiguous conclusions about their relationships (see Supplementary Methods 1.7). Nevertheless, the ambiguous placements of these two latter families had little impact in inferring the evolution of hearing and sound-producing organs.

**Fig. 1:**
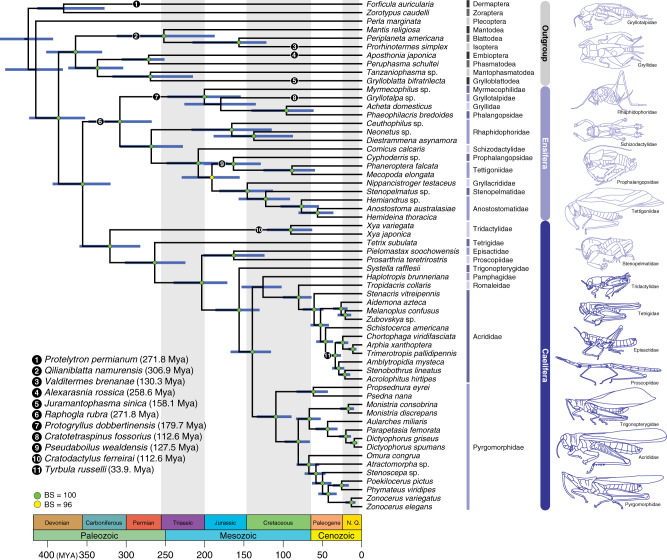
Dated phylogeny of Orthoptera based on the phylogenomic data. This chronogram is a result of a divergence time estimate analysis based on the most decisive data set (D_aa,trans,strict_) consisting of 436,488 aligned amino acids. Bootstrap support (BS) values are indicated by coloured nodes (green: BS = 100; yellow: BS = 96); values below 90 are not shown. Divergence time estimates were calculated using 86,043 amino-acid sites and 11 fossil calibrations (species names and dates listed on figure). Blue bars indicate 95% credibility intervals of node ages. Geological timescale is shown at the bottom. Additional details on data generation and analyses can be found in Supplementary Methods 1 and 2.

**Fig. 2:**
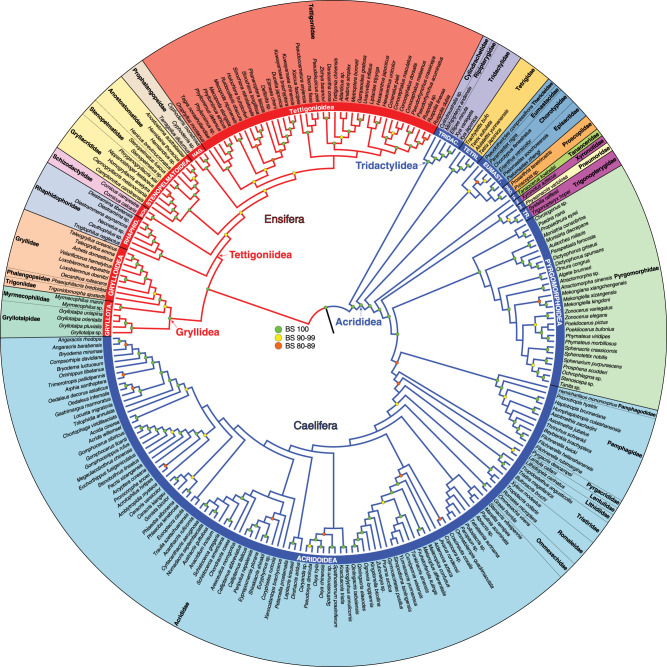
Comprehensive phylogeny of Orthoptera. This phylogeny is estimated based on analyses of data from transcriptomes and mitochondrial genomes (D_nt,trans+mito,strict_). The tree is derived from a maximum likelihood analysis of 448,861 aligned sites of nucleotides. Bootstrap support (BS) values are indicated by coloured nodes (green: BS = 100; yellow: BS = 90-99; orange: BS = 80-89). Red branches indicate the suborder Ensifera and blue branches indicate Caelifera. The red and blue clade names indicated by arrows (Gryllidea, Tettigoniidea, Tridactylidea and Acrididea) are infraorder names. The names in white, in red or blue bars are superfamily names. Broad circular bars are colour-coded by superfamily. TRIDAC Tridactyloidea, TETRI Tetrigoidea, EUMAST Eumastacoidea, PRO Proscopioidea, TA Tanaoceroidea, TR Trignopterygoidea, PN Pneumoroidea, GRYLLOTA Gryllotalpoidea, RHAPHID Rhaphidophoroidea, SCH Schizodactyloidea, HAG Hagloidea.

Our analyses confirmed the monophyly of Orthoptera and its two suborders, Ensifera and Caelifera (Figs. 1, 2). Moreover, we recovered a comparatively ancient age for crown-Orthoptera, at ~355 million years ago (Mya) [95% credibility interval (CI), 393.8–320.0 million years (My)] (Fig. 1), which is ~63 My earlier than a previous estimate for this group^34^. We estimated crown-Ensifera to have appeared during the Late Carboniferous (308 Mya; CI, 348.0–267.4 My) (Fig. 1), which is consistent with the known fossil record, with the earliest stem-Ensifera being 272 million-years-old^31,32^. Our analyses recovered two monophyletic infraorders within this group, Gryllidea and Tettigoniidea, the former consisting of Grylloidea (including Gryllidae, Phalangopsidae, Trigonidiidae and Mogoplistidae), Gryllotalpidae and Myrmecophilidae, and the latter consisting of the remaining families (Figs. 1, 2). We inferred that crown-Gryllidea originated in the late Triassic or early Jurassic (200 Mya; CI, 247.5–154.1 My) (Fig. 1). Crown-Tettigoniidea originated in the Permian (268 Mya; CI, 308.1–227.7 My) and diverged into its major extant lineages throughout the Mesozoic (Fig. 1). Within Tettigoniidea, we recovered the following family-level relationships: (Rhaphidophoridae (Schizodactylidae ((Gryllacrididae (Stenopelmatidae + Anostostomatidae)) + (Prophalangopsidae + Tettigoniidae)))) (Fig. 2). We estimated that crown-Caelifera originated in the Carboniferous (320 Mya; CI, 359.5–282 My), and our analyses recovered two monophyletic infraorders (Figs. 1,2), Tridactylidea and Acrididea, the former consisting of Cylindrachetidae, Ripipterygidae and Tridactylidae, which diverged in the late Carboniferous, and the latter consisting of the remaining families. The more diverse Acrididea originated in the Late Permian (263 Mya; CI, 301.5–224.6 My) and split into two monophyletic groups, Tetrigidae and the superfamily group Acridomorpha (grasshopper-like insects) (Fig. 1). Most modern grasshopper diversity arose in the Cenozoic (Fig. 1). Additional details regarding the specific relationships within Orthoptera are described in Supplementary Methods 1.9.

### Evolution of hearing and sound-producing organs in Orthoptera

**Fig. 3:**
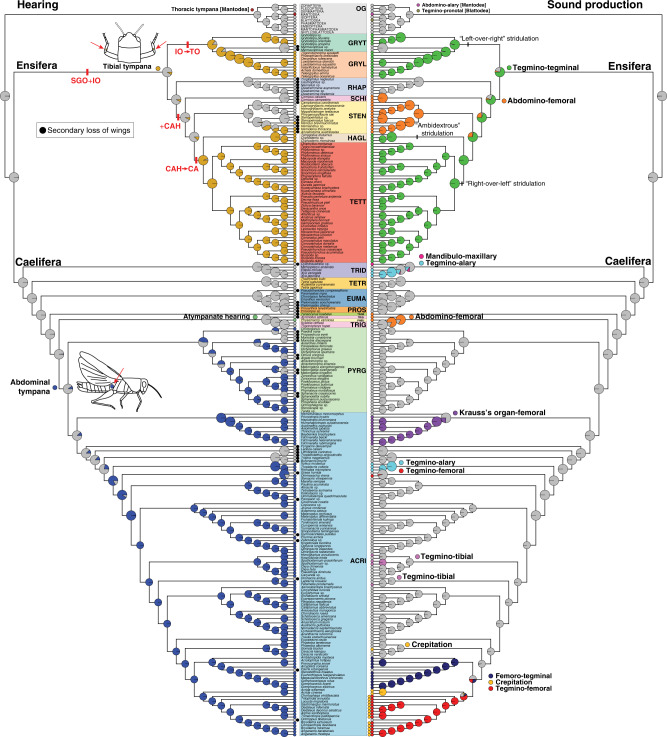
Ancestral character state reconstruction of hearing and sound-producing organs. The topology used for this analysis is the comprehensive phylogeny based on D_nt,trans+mito,strict_ (presented in Fig. 2). The coloured circle at each branch tip indicates the character state of the corresponding species, with grey circles indicating the absence. The coloured circle at each node shows the probability of each ancestral character state. On the left, the character evolution of hearing organs is shown and the character states are colour-coded. In addition to the ancestral character state reconstruction, two additional traits are mapped. The first trait is the internal sensory organs in the ensiferan foretibia, shown in red. The ancestral condition for Ensifera is the presence of the subgenual organ (SGO) and the intermediate organ (IO). In the common ancestor of Gryllidea, IO was modified to tibial organ (TO), Rhaphidophoridae retains the ancestral SGO + IO. In the common ancestor of Schizodactyloidea, Stenopelmatoidea, Hagloidea and Tettigonioidea, a novel third component known as crista acustica homologue (CAH) evolved. In the common ancestor of Hagloidea and Tettigonioidea, CAH was modified to an auditory sensory organ called crista acustica (CA). The second trait is the loss of wings, which is indicated by black circles. Often, the species that lack tympanal hearing also have lost wings. On the right, the character evolution of sound-producing organs, in the form of stridulatory apparatus, is shown, and the character states are colour-coded. We used a specific naming convention in which the first-named structure has the stridulatory file and the second named structure has the scraper. For example, abdominal-femoral stridulation would have the stridulatory files on the abdomen and the scraper on the inner side of hind femora. Different mechanics of tegmino-tegminal stridulation are mapped onto the phylogeny. The common ancestor of Gryllidea evolved “left-over-right” stridulation, the common ancestor of Hagloidea evolved “ambidextrous” stridulation, and the common ancestor of Tettigonioidea evolved “right-over-left” stridulation. OG Outgroups, GRYT Gryllotalpoidea, GRYL Grylloidea, RHAP Rhaphidophoroidea, SCHI Schizodactyloidea, STEN Stenopelmatoidea, HAG Hagloidea, TETT Tettigonioidea, TRID Tridactyloidea, TETR Tetrigoidea, EUMAS Eumastacoidea, PROS Proscopioidea, TANA Tanaoceroidea, TRIG Trignopterygoidea, PNEU Pneumoroidea, PYRG Pyrgomorphoidea, ACRI Acridoidea.

Using ancestral character state reconstruction, we found lineage-specific patterns of evolution for hearing and sound-producing organs in Orthoptera (Fig. 3). In Ensifera, our analysis found that tegmino-tegminal stridulation likely evolved in the common ancestor of all extant lineages (Fig. 3). It is secondarily absent in Myrmecophilidae and Rhaphidophoridae, but as a consequence of the complete loss of wings in these families. Genuine loss occurred in Schizodactylidae and the common ancestor of Gryllacrididae, Stenopelmatidae and Anostostomatidae (Fig. 3), but in these families, abdomino-femoral stridulation evolved instead, known to produce defensive signalling against predators^29,30^. As for the hearing organs, we inferred that tibial tympana evolved at least three times in Ensifera (Fig. 3), once in the common ancestor of Gryllidea, once in the common ancestor of Anostostomatidae, and once in the common ancestor of Prophalangopsidae and Tettigoniidae. However, our analysis did recover a small probability that tibial tympana evolved in the common ancestor of Ensifera (Fig. 3), and thus, we could not completely rule out the possibility that the presence of tibial tympana was the ground plan for the suborder as well. We also examined the evolution of the complex tibial organ in the forelegs based on character mapping (Fig. 3), and found that the ancestral Ensifera had the tibial organ consisting of the subgenual organ (SGO) and the intermediate organ (IO), and the common ancestor of Gryllidea gained far-field hearing by evolving the tympanal organ (TO), which was modified from the IO^7^, and the tibial tympana. Rhaphidophoridae retained the ancestral configuration, but in the common ancestor of Tettigoniidea, a third component known as the crista acustica homologue (CAH) evolved. With the modification of this third component as the crista acustica (CA) and with the evolution of tibial tympana, the common ancestor of Prophalangopsidae and Tettigoniidae gained far-field hearing.

Within Caelifera, stridulatory organs evolved at least 10 times across the phylogeny based on our current taxon sampling, involving many different body parts (Fig. 3). However, the ability of these structures to produce sound remains largely unconfirmed, except for a few species that use acoustic signalling for mating or defence^11,35,36^, and thus, we must consider them putative for now. Definitive sound-producing organs used for mating evolved at least three times in Caelifera (Fig. 3), once in the common ancestor of Pneumoridae using abdomino-femoral stridulation, once in the common ancestor of Pamphagidae using Krauss’s organ-femoral stridulation, and once in the common ancestor of acridid subfamilies Acridinae, Gomphocerinae and Oedipodinae using hind femora and tegmina, although the location of the stridulatory file varies within these insects^36^. Our analysis showed that abdominal tympana likely evolved at least three times (Fig. 3), once in the common ancestor of Pyrgomorphidae, once in the common ancestor of Pamphagidae, and once in the common ancestor of Romaleidae, Ommexechidae and Acrididae. Similar to the case of tibial tympana in Ensifera, our ancestral character state reconstruction recovered a small probability that abdominal tympana evolved in the common ancestor of all these tympanate lineages (Fig. 3), and thus the presence of this structure could have been the ground plan for them as well.

**Fig. 4:**
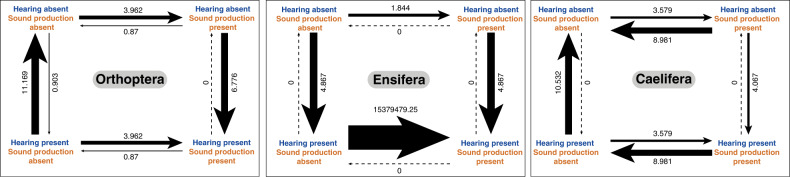
Evolutionary correlation between hearing and sound production in Orthoptera. Pagel’s test for evolutionary correlation was calculated between hearing and sound production in Orthoptera and its two suborders, Ensifera and Caelifera. The thickness of arrows corresponds to the rate of change from one combination of trait states (i.e. no hearing and no sound production) to another combination (i.e. no hearing and sound production). The higher the rate, the thicker the arrow. In each of the three analyses, there is a strong evolutionary correlation between hearing and sound production, but the patterns are different. In Orthoptera as a whole, the strongest transition rate is from hearing present & sound production absent to both hearing and sound production absent. In Ensifera, the strongest rate is from hearing present & sound production absent to both hearing and sound production present. In Caelifera, the strongest transition rate is from hearing present and & sound production absent to both hearing and sound production absent. The differences between Ensifera and Caelifera show that the co-evolutionary dynamics between hearing and sound production differ between the two lineages.

We performed Pagel’s^37^ binary character correlation test to determine whether hearing and sound-producing organs co-evolved within Orthoptera and within each of its suborders (Fig. 4). For all comparisons, we recovered statistically significant correlation between the two organs, but their co-evolutionary dynamics were different depending on the lineages (Fig. 4, Supplementary Methods 3.2). Considering Orthoptera as a whole, the best-supported model was that the evolution of hearing organs depended on the evolution of sound-producing organs (weighted AIC = 0.7685). Specifically, there were much higher instances of the absence of hearing organs when sound-producing organs were absent, and of the presence of hearing organs when sound-producing organs were present (Fig. 4). However, because taxon sampling is known to affect correlation analyses^38^ and because the known patterns of acoustic communication are very different between Ensifera and Caelifera^11^, we examined the patterns of character correlation for each suborder, which delivered highly contrasting patterns (Fig. 4). For Ensifera, the model that the evolution of sound-producing organs depended on that of hearing organs (weighted AIC = 0.4490) and the model that the evolution of hearing organs depended on that of sound-producing organ (weighted AIC = 0.4343) similarly explained the pattern. Of all possible interactions between the two organs, we found magnitudes higher instances of the presence of sound-producing organs when hearing organs were present (Fig. 4), which indicates that nearly all ensiferans that can hear also produce sound, suggesting an extremely high correlation between the two traits. For Caelifera, the model that the evolution of hearing organs depended on that of sound-producing organs (weighted AIC = 0.4361) best explained the data, but the model that the evolution of sound-producing organs depended on that of hearing organs (weighted AIC = 0.3567) also reasonably explained the data. We found that there were higher instances of the absence of sound-producing organs regardless of the presence or absence of hearing organs in Caelifera (Fig. 4), almost an opposite pattern from what we found in Ensifera.

### Rates of lineage diversification in relation to acoustic communication

**Fig. 5:**
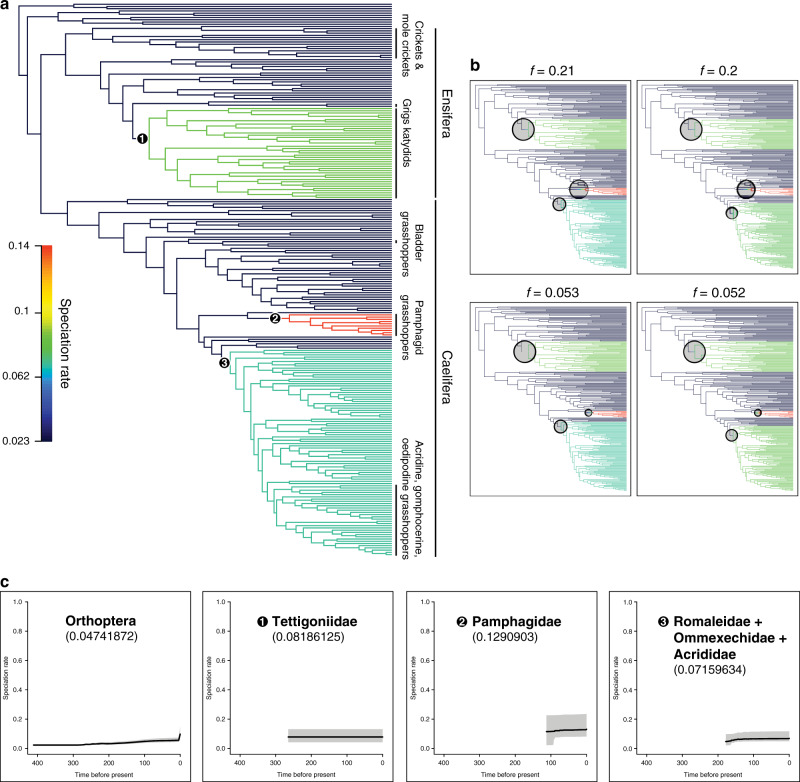
Bayesian analysis of macroevolutionary mixtures for Orthoptera. The ultrametric tree used in this analysis is the dated phylogeny based on the combined data (D_nt,trans+mito,strict_). **a** Phylorate plot showing speciation rates (cool colours = slow, warm = fast; specific rate range shown in the vertical colour legend) along each branch of the Orthoptera phylogeny. The three clades indicated by the black circled nodes are the clades with increased rate shifts. Lineages that show acoustic communication are indicated with vertical lines near the terminals. Branches are coloured according to the rate shifts. **b** About 95% credible sets of macroevolutionary shift configurations. *f* value of 0.21 indicates that 21% of the samples in posterior can be assigned to shift configuration shown in the upper left plot. These four shift configurations collectively account for 51.5% of the posterior distribution. **c** Clade-specific evolutionary rate variation through time for Orthoptera and the three lineages identified to have rate shifts.

The Bayesian analysis of macroevolutionary mixtures (BAMM)^39^ on our larger data set found three episodes of rate shift along the phylogeny of Orthoptera (Fig. 5). The first episode of rate shift took place in the common ancestor of Tettigoniidae, during the Cretaceous, with a mean clade-specific evolutionary rate for the family (0.08186125) nearly double the background rate for Orthoptera (0.04820052) as well as for Ensifera (0.04996942) (Fig. 5). The second episode of rate shift took place in the common ancestor of Pamphagidae, during the late Cretaceous and the early Paleogene, with a mean clade-specific rate (0.1290903) almost tripling the background rate for Orthoptera as well as for Caelifera (0.04905859), which was retrieved as the highest evolutionary rate among all orthopteran lineages (Fig. 5). The third episode of rate shift took place in the common ancestor of Romaleidae, Ommexechidae and Acrididae, during the late Cretaceous and throughout the Paleogene, with a mean clade-specific rate (0.07159634) slightly higher than the background rate (Fig. 5). Interestingly, however, we found that other singing lineages within Ensifera, namely Grylloidea, Gryllotalpidae and Prophalangopsidae, did not show any discernible rate shift (Fig. 5). We tried not to over-interpret the recovered patterns from this analysis, because the appropriateness of BAMM in diversification analyses has been questioned, especially concerning its ability to accurately estimate diversification rates^40^ although the developers of BAMM have argued that these criticisms were unjustified^41^.

**Fig. 6:**
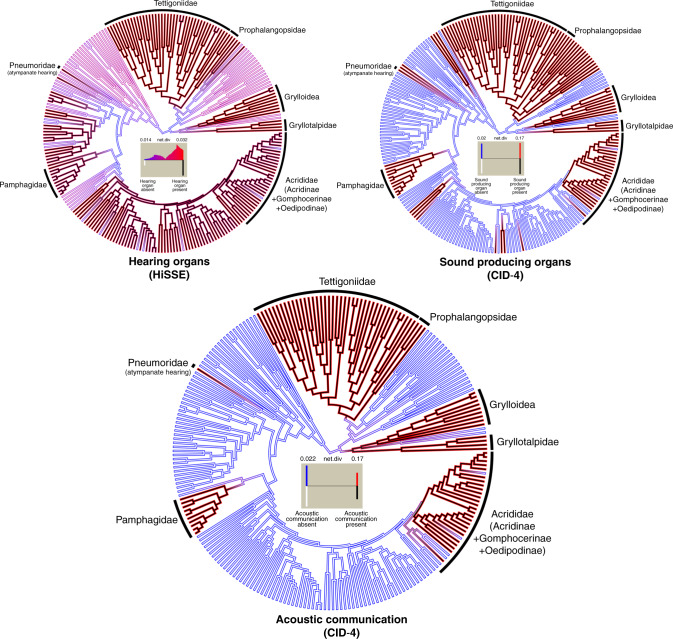
Models of trait-dependent diversification. Character reconstruction of states and net diversification rates estimated using multimodel inference methods implemented in *hisse*. Shown here are the best-fitting models for each tested trait (presence/absence of hearing organs, of sound hearing organs and of acoustic communication) from the 24 models of trait-dependent and trait-independent diversification models. All clades that are characterised by having sexual communication using acoustic signalling are labelled in the circular trees, and estimates of the most likely state and rate are based on the model-averaged marginal reconstructions inferred under the best-fitting models. The histograms inside the trees show the location of the rates on a gradient of rates, as well as the frequency of both these rates and states for each contemporary tip taxa. For hearing organs, the best-fitting model was one of the HiSSE models, but for both sound-producing organs and acoustic communication, the best-fitting model was one of the trait-independent diversification models (CID-4).

We fitted various models of trait-dependent and trait-independent diversification using HiSSE (Hidden State Speciation and Extinction)^42^ to test whether the evolution of hearing and sound-producing organs affected speciation and extinction rates of different orthopteran lineages (Fig. 6). For the hearing organs, the best-fitting model, according to AIC scores, was one of the HiSSE models, which suggests character-dependent diversification where all the diversification parameters are free and where transitions between hidden states of hearing-organ-absent and hearing-organ-present were disallowed (HiSSE, q0B1B = 0, q1B0B = 0, all other q’s equal). We found a higher net diversification rate associated with the presence of hearing organs, which is likely due to a higher diversification rate of the hidden state (Fig. 6). The net diversification rate associated with the absence of hearing organs was relatively lower. For the sound-producing organs, the best-fitting model was one of the CID (trait-independent) models, which assumes that the evolution of a binary trait (presence or absence of sound-producing organ) is independent of the diversification process without forcing the diversification process to be constant across the tree (CID-4: q’s equal) (Fig. 6). In other words, the selected model suggested that the evolution of sound-producing organs did not affect the net diversification rate. When acoustic communication was coded as a binary trait, the best-fitting model was the identical trait-independent model selected for the sound-producing organs (CID-4: q’s equal), which suggested that diversification process was independent from the evolution of acoustic communication (Fig. 6).

## Discussion

Orthopteran insects, such as crickets, katydids and grasshoppers, have been model systems for studying acoustic communication for decades^2,7,11,18,30,43–46^, but how hearing and sound-producing organs originated and evolved throughout the diversification of these insects has remained elusive due to the lack of a well-resolved phylogeny. This work firmly establishes phylogenetic relationships among the major lineages and divergence time estimates within Orthoptera based on phylogenomic data and carefully selected fossil calibration points. We find that crown-Orthoptera likely originated 355 million years ago, and diverged into Ensifera and Caelifera in the Carboniferous (Fig. 1). Our study suggests that these two suborders have each followed very different, lineage-specific patterns of evolution for hearing and sound-producing organs (Figs. 3, 4).

### Hearing and sound-producing organs co-evolved in Ensifera

Ensifera, the larger of the two suborders, encompasses ~15,500 extant described species, many of which are nocturnal and use acoustic signalling as a primary mode for sexual communication. The singing ensiferans include four extant lineages (crickets [Grylloidea, including Gryllidae, Phalangopsidae, Trigonidiidae and Mogoplistidae], mole crickets [Gryllotalpidae], katydids [Tettigoniidae] and grigs [Prophalangopsidae]), which have specialised hearing sensory organs in the form of tympanal ears located on front tibiae and a stridulatory apparatus on male tegmina (forewings)^3^. They account for nearly 85% of the ensiferan diversity^3,17,47,48^. The remaining ensiferan lineages have neither tibial tympana nor stridulatory tegmina (ant-loving crickets [Myrmecophilidae] and cave crickets [Rhaphidophoridae]), or lack tibial tympana but possess a stridulatory apparatus on the abdomen, used for defensive signalling^29,30,49^ and present in both sexes and in nymphs (splay-footed crickets [Schizodactylidae], raspy crickets [Gryllacrididae], Jerusalem crickets [Stenopelmatidae] and some king crickets and wetas [Anostostomatidae]). The monophyly of Ensifera has been consistently supported by all modern cladistic analyses^34,43,50,51^, and most researchers agree that the suborder consists of two monophyletic infraorders, Gryllidea and Tettigoniidea^34,51^, which our study also confirmed (Figs. 1,2). However, there has not been a consensus on the internal relationships among families and superfamilies, as different phylogenetic studies utilising different character systems (morphology, ribosomal RNAs or mtgenomes) disagreed with each other^34,43,50,52^, leading to conflicting inferences about the evolution of acoustic communication^7,11,43,53^. Especially, whether the stridulatory apparatus evolved once or multiple times has been contentious^32,43,53^. Our phylogenomic analysis recovered strongly supported relationships among the families (Figs. 1,2), which are more congruent with a morphology-based phylogeny^43^ than with the previous molecular studies^34,50–52^. Based on the recovered topology and divergence time estimates (Figs. 1,2), as well as ancestral character state reconstruction (Fig. 3), we can infer the following evolutionary scenario regarding how hearing and sound-producing organs might have evolved in Ensifera.

Between the late Carboniferous and the early Permian, crown-Ensifera diverged (Fig. 1) and male-specific tegmino-tegminal stridulation evolved in the common ancestor of Ensifera (Fig. 3), which represents one of the earliest occurrences of airborne sound generation in animals. Although the oldest fossil ensiferans (such as *Gryllavus* and *Protogryllus*) with a well-preserved stridulatory apparatus homologous to the present-day structure are known from the Triassic^31,32^, our finding suggests that a similar mechanism of sound production could have evolved much earlier. The earliest insectivorous tetrapods appeared in the early Carboniferous, and these animals did not have tympanic ears^6^. It has been hypothesised that these predators would have been deterred by stridulation produced by insect prey upon seizure, which would have stimulated their tactile receptors that caused them to release the prey^6,54,55^. In a sense, stridulation could have originally evolved as part of a deimatic behaviour^56^. Fossil evidence shows that specialised sound-producing organs involving wings were present among the Permian and Triassic stem-Orthoptera^31,32,57,58^. If we accept the possibility that the ability to move wings to produce sound was an ancient invention during the early diversification of Orthoptera, it is conceivable that this behaviour could have been co-opted for sexual communication, possibly in parallel within several lineages. For instance, the stem-orthopteran lineage Titanoptera had modified veins in the forewings highly indicative of sound production, present in both sexes^57,58^ and possibly used for pair formation via reciprocal duetting^31,59^. This group evolved from the Permian ‘tcholmanvissiids’^59^, which lack specialised forewing sound-producing organs, if any. Another contemporaneous lineage of stem-orthopterans, the Mesoedischiidae, had male-specific tegmino-tegminal stridulation, although the specific veins modified for sound production were not homologous to those in extant Ensifera^57,58^. Among the four extant singing ensiferan lineages, the specific mechanics of tegmino-tegminal stridulation are known to differ^43^. Crickets and mole crickets stridulate by moving the left forewing over the right, and katydids stridulate in the opposite way by moving the right forewing over the left^43^. Grigs are able to stridulate by moving their wings both ways^43^. Moreover, a recent comparative morphological analysis proposed that the stridulatory apparatus involved different veins of the forewing in these four lineages^53^, although the case remains debated. Regardless, it can be argued that the muscular mechanics and associated neurocircuit enabling male-specific tegmino-tegminal stridulation are phylogenetically conserved and potentially plesiomorphic in Ensifera, but different lineages independently evolved different ways of creating audible sound, building on the same physiological mechanism.

Our analysis suggests that tegmino-tegminal stridulation was secondarily lost in several ensiferan lineages (Fig. 3), and this loss is often associated with adaptations to novel environments that promote the loss of wings. For example, extant members of Rhaphidophoridae are completely apterous and often associated with caves^60^. Similarly, members of Myrmecophilidae are wingless and intimately associated with ant colonies^61^. Many members of Schizodactylidae, Gryllacrididae, Stenopelmatidae and Anostostomatidae are specialists on subterranean habitats and wingless as well^62^. However, each of these four latter families includes some species with fully functional tegmina lacking stridulatory apparatus^63^. It has been documented that several cricket and katydid species have secondarily lost the ability to sing^47,48^, and one well-documented case, that of the Hawaiian cricket *Teleogryllus oceanicus*, demonstrates that the loss of stridulatory apparatus repeatedly and convergently evolved due to a strong selective pressure from an introduced phonotactic parasitoid fly, *Ormia ochreacea*^64^. This loss has a genetic basis in the form of a simple alteration of a master regulatory switch during early development that can lead to the dramatic change in the adult phenotype^65^. While it is difficult to attribute the same process to explain the loss of tegmino-tegminal stridulation in the non-singing ensiferans, we conclude that this loss of complex trait could have been achieved easily multiple times during the diversification of Ensifera.

Interestingly, many of the non-singing ensiferans are known to engage in some type of intraspecific communication using substrate-borne vibration, drumming using abdomen or legs, or tremulation (shaking without any substrate)^29,49,63,66,67^, and have well-developed chordotonal organs for sensing vibration^7,68^. Our analysis also shows that abdomino-femoral stridulation likely evolved at least twice (Fig. 3), once in the common ancestor of Schizodactylidae and once in the common ancestor of Gryllacrididae, Stenopelmatidae and Anostostomatidae. This mechanism is found in both sexes as well as in nymphs, and it is not used for sexual communication, but for producing defensive signal against predators^29,30^. These patterns collectively suggest that the loss of tegmino-tegminal stridulation could have promoted the evolution of both vibratory signalling in a sexual context, and an alternative acoustic signalling in a non-sexual context in these non-singing lineages.

For hearing, it is less clear whether the first hearing organs also evolved in the common ancestor of Ensifera based on our current data. Hearing organs in the forelegs are complex organs consisting of external tympana as well as internal complex tibial organs^7,14,69^. It is unclear what the original form of sound detection was in the ancestral ensiferans, but it is conceivable that thin cuticle of foretibia could have initially functioned as a resonator for the internal sensory organs to pick up sound wave. We infer that thinning of the cuticle evolved at least three times to give rise to tympanal membrane within this group (Fig. 3). However, the neurophysiological mechanisms underlying hearing independently evolved twice (Fig. 3), leading to two different types of hearing sensory organs, SGO + TO found in crickets and mole crickets, and SGO + IO + CA found in katydids and grigs. These results are consistent with the idea that the common ancestor of Ensifera probably did not have the structures enabling far-field hearing, but different lineages independently evolved far-field hearing. Although it is generally hypothesised that the early form of hearing in insects evolved in the context of detecting and avoiding predators^11,12,25^, the specific position where tympana evolved in the singing ensiferans raises an intriguing possibility that hearing in Ensifera could have evolved in another context. Extant ensiferan ears usually have two auditory inputs, with sound arriving at the external surface of the tibial tympana, and also internally via the acoustic trachea, which open on the acoustic spiracles at the side of the pronotum^44,70–72^. These ears are pressure difference receivers^73,74^, as the sound travelling internally on the trachea travels slower and a longer distance than that reaching the external surface of the tympanum from the outside at the normal speed of sound propagation in air. This causes differences in gain between sound arriving externally and internally^72,74,75^. This complex acoustic tracheal system also shows lineages-specific differences. In crickets and mole crickets, acoustic trachea connect all four sound inputs with an enlarged part in its midline, accompanied by two thin septa originating from each trachea^72,76^. In katydids and grigs, the acoustic trachea starting at the acoustic spiracles do not connect in the middle, such that the trachea starting from the right and left acoustic spiracles connect to the right and left tibial tympana, respectively^70,75,77^. In katydids, the tracheae are enlarged as acoustic bullae at the acoustic spiracles and gradually narrow as they approach the tympanal ears^47,70^. Therefore, we conclude that this elaborate directional hearing mechanism evolved independently in the context of accurately locating the source of conspecific calls.

### Sound-producing organs and hearing organs evolved separately in Caelifera

Caelifera is the other of the two orthopteran suborders, with ~12,200 extant species, and consisting of familiar insects such as grasshoppers and locusts, as well as the lesser-known pygmy mole crickets, pygmy grasshoppers, monkey grasshoppers, stick grasshoppers, and their relatives^34^. Sexual communication using acoustic signalling is a relatively rare feature across Caelifera, which has only been documented in a small number of divergent families (bladder grasshoppers [Pneumoridae], pamphagid grasshoppers [Pamphagidae], tooth-legged grasshoppers [Acrididae: Gomphocerinae] and banded-wing grasshoppers [Acrididae: Oedipodinae])^11,78^. Our literature survey shows that these lineages each use different stridulatory mechanisms to produce sound (Fig. 3), but they all involve rubbing hind femora up and down against other body parts, such as thickened veins on tegmina or specialised areas on the abdomen. We find that most of the early-diverging caeliferan lineages do not have hearing organs, and tympanal hearing is only found in a few grasshopper families (Pamphagidae, Pyrgomorphidae, Romaleidae, Ommexechidae and Acrididae) (Fig. 3), which originated in the Cretaceous and the Paleogene (Fig. 1). When present, tympana are located on both sides of the first abdominal segment, which usually have large tympanal membranes that are innervated with the auditory sensory organs, and externally encircled by sclerotised rings, with air-filled tracheal sacs internally positioned between the tympanal membranes^45^. According to our phylogenomic analysis (Fig. 2), which recovered relationships that are largely congruent with previous studies^34,51,79,80^, hearing and sound-producing organs in Caelifera did not evolve jointly, but followed different evolutionary trajectories (Fig. 3). There is no fossil evidence to suggest the antiquity of hearing or sound production in Caelifera, and we deduce that acoustic communication is generally a more recent invention in Caelifera compared to Ensifera.

Our study shows that, throughout the diversification of Caelifera, several lineages evolved paired structures equipped with a stridulatory file on one body part and a scraper on another body part (Fig. 3), which involve mouthparts, forewings and hindwings, middle legs and hind legs and abdomen. However, it is largely unconfirmed whether these paired structures are actually used for sound production, except for the aforementioned families that use acoustic signalling. It is also not clear in what context these structures evolved. For example, these structures are found in both sexes and in nymphs in some lineages (e.g. mandibulo-maxillary stridulation found in Cylindrachetidae)^81^, which could have evolved in the context of defence. Similarly, these putative sound-producing organs are found only in males in some lineages (e.g. abdomino-femoral stridulation found in Tanaoceridae)^82^, which could have evolved in a sexual context. In other words, there is much to be learned in terms of the diversity, mechanisms and functions of sound production in Caelifera. Intriguingly, none of the caeliferans is known to engage in tegmino-tegminal stridulation, which is the primary and phylogenetically conserved sound-producing mechanism in Ensifera. This implies that the neurophysiological machinery enabling tegmino-tegminal stridulation was never part of the caeliferan ground plan.

Our study finds that the first form of sexual communication using acoustic signalling in Caelifera likely evolved in the common ancestor of the South African family Pneumoridae, in the Jurassic (Figs. 2,3). By this time, complex acoustic signalling was already well-established in Ensifera. Extant bladder grasshopper males, which are fully winged, have an inflated abdomen that functions as a resonating chamber to produce loud low-frequency calls that can travel up to 2 km using abdomino-femoral stridulation^83^. In response to male calling, receptive females, which are flightless, indicate their willingness to mate by acoustically responding, which leads to pair formation via reciprocal dueting^84^. Female sound-producing organs are not homologous to those in males and different species use different body parts to create sound (V. Couldridge, personal communication). Interestingly, both males and females lack tympanal ears, and instead have chordotonal organs innervating each abdominal segment, and as such, the entire abdomen functions as a hearing organ^46^. This pattern suggests that there could have been a selective pressure for evolving acoustic communication as early as the Jurassic, but perhaps because dedicated directional hearing organs did not yet evolve. These lineages never radiated like their ensiferan counterparts did.

It was not until the Cretaceous that abdominal tympana appeared in Caelifera (Figs. 1,3). Our finding is more consistent with the idea of multiple origins of abdominal tympana, although we did recover a small probability that the common ancestor of Pyrgomorphoidea and Acridoidea could have evolved abdominal tympana once (Fig. 3). An intermediate option would involve a rather unspecialised, early form of abdominal hearing organ which might have then undergone parallel evolution, towards proper abdominal tympana, within the different lineages. The context in which these hearing organs evolved is not clear. Grasshoppers with abdominal tympana generally show jumping or flying behaviour upon hearing approaching sound^27^, which indicates that its current function is most likely for detecting predators or disturbances, and this is indeed the most commonly invoked hypothesis on the origin of grasshopper ears^11,23^. However, insectivorous predators were already well-diversified by the Cretaceous^6^ and it is unlikely that a sudden and strong selective pressure triggered the evolution of predator-detection hearing. There were also other caeliferan lineages that radiated without evolving hearing, such as Tetrigoidea and Eumastacoidea, and these insects faced predators, yet succeeded without tympana. Given that most grasshopper species with abdominal tympana do not have sexual communication using acoustic signalling, it is also difficult to think that hearing evolved in a sexual context. One alternative explanation comes from our observation that secondary loss of abdominal tympana is often found in those species that evolved wing reduction or loss^85^, which suggests that there could be a connection between flight and hearing. The physiological mechanisms of the auditory pathway in grasshoppers and locusts have been intensely studied^45^, and it has been shown that auditory information processing through abdominal tympana is in fact intimately influenced by thoracic muscle movement and wingbeat noise during flight^86^. Although the ability to fly is a plesiomorphy for Orthoptera, Pyrgomorphoidea and Acridoidea are the first large-bodied caeliferans with an exceptionally strong dispersal capacity, which raises an intriguing possibility that abdominal tympana could have originally evolved in the context of modulating flight, rather than detecting disturbances or locating mates. This idea is indirectly bolstered by the pattern that many brachypterous katydids and crickets still retain the ability to hear through tibial tympana^47,48^, which are probably not involved in flight modulation.

The evolution of abdominal tympana in early grasshoppers could have led to the evolution of sexual signalling under the ‘sensory-bias’ mechanism^11^, which we think was achieved by the independent evolution of sound-producing organs in two grasshopper lineages, Pamphagidae and a monophyletic group within Acrididae consisting of Acridinae, Gomphocerinae and Oedipodinae (Fig. 3). However, we find that the path to evolving acoustic communication differed considerably between the two. Pamphagidae is a large-bodied family that originated in the Cretaceous (Fig. 1). Similar to the bladder grasshoppers, pamphagid grasshopper engage in pair formation via reciprocal dueting^78^, and males are often fully winged and females are flightless, although the loss of wings is quite common in this family^35^. Our study finds that Krauss’s organ-femoral stridulation is a phylogenetically conserved mechanism of sound production for the family (Fig. 3). The Krauss’s organ is a specialised plate located on the lower anterior corners of the second abdominal tergite, which is rubbed by the ridges inside hind femora^87^. This mechanism is present in both males and females, and the sound produced by this mechanism is species-specific^35^. Although not included in our taxon sampling, many pamphagids are also known to utilise other types of sound-producing mechanisms for mating, involving abdomen, hind femora, forewings, hindwings, middle tibiae and thorax^35,78^. These collectively suggest that the evolution of sound production occurred in the common ancestor of Pamphagidae, which already had the ability to hear, and this could have led to the elaboration of acoustic communication in the entire lineage.

On the other hand, sound production evolved much later in Acrididae, after the lineage has already diversified (Fig. 3). We find that the presence of abdominal tympana is plesiomorphic for the family (Fig. 3), and the male-specific stridulatory mechanism using tegmina and hind femora likely evolved between the Eocene and the Oligocene in the common ancestor of Acridinae, Gomphocerinae and Oedipodinae, likely in a sexual context. However, even within this lineage, the sound-producing organs followed different evolutionary trajectories in terms of specific modifications of the stridulatory apparatus. For example, in Gomphocerinae, stridulatory pegs are located on the hind femora, which rub against the thick veins in the forewings, whereas in Oedipodinae, a row of stridulatory files on the intercalary veins in the forewing rubs against the scrapers in the hind femora^36^. In addition to the stridulatory signalling, Oedipodinae and some members of Acridinae evolved an alternative and non-stridulatory acoustic mechanism, called crepitation, which produces sound by snapping wings when they fold and unfold^36^. In all these grasshoppers, acoustic signalling is often complemented with visual signalling, such as leg movements, characterising a multimodal sexual selection^36^. Thus, acoustic signalling found in Acrididae represents the most recently evolved form of sexual communication within Orthoptera.

### Evolution of acoustic communication did not influence diversification rates in Orthoptera

We have shown that the evolution of sexual communication using acoustic signalling in Ensifera and Caelifera each followed a very different trajectory (Figs. 3, 4). In Ensifera, we infer that tegmino-tegminal stridulation was an ancestral feature that could have evolved as defensive signalling in crown-Orthoptera, and different lineages independently evolved tibial tympana in a sexual context. In each common ancestor of the singing lineages, both hearing and sound-producing organs were present, allowing the Fisherian mechanism to shape the co-evolution between female perception and male signalling devices^11^. Our Pagel’s binary character correlation test found overwhelming evidence that hearing and sound-producing organs co-evolved in Ensifera (Fig. 4), supporting this hypothesis. In Caelifera, abdominal tympana evolved later in the lineage diversification (Fig. 3), possibly in the context of modulating flight in large-bodied grasshoppers, which was later co-opted for detecting predators, and again co-opted for sexual communication when male-specific sound-producing organs evolved independently in different lineages. This pattern fits well with the ‘sensory bias’ mechanism. The Pagel’s test found little support for the co-evolution between hearing and sound-producing organs (Fig. 4), thus supporting the alternative hypothesis. Having established these evolutionary mechanisms, we now ask whether the evolution of hearing and sound-producing organs affected diversification rates in different lineages that use acoustic signalling in a sexual context.

It is generally accepted that sexual selection is a major driving evolutionary force shaping the diversification of singing insects^88,89^, and theory predicts that sexually selected traits tend to evolve rapidly^88,90^. Especially, if the inferred mechanism for the evolution of hearing and sound-producing organs is the Fisherian mechanism, we would expect an elevated diversification rate in a clade that is characterised by sexual communication using acoustic signalling^89^. This idea was recently tested in tetrapods but, surprisingly, it was found that acoustic communication did not increase diversification rates in these animals^4^. To test this proposal in Orthoptera, we first performed a diversification analysis using BAMM^39^ to determine clade-specific evolutionary rates. Among the lineages with known acoustic communication, we find that Tettigoniidae was the only lineage within Ensifera to show an increased mean clade-specific evolutionary rate, while other lineages (Grylloidea, Gryllotalpidae and Prophalangopsidae) did not show any discernible rate shifts. Likewise, Pamphagidae was the only lineage within Caelifera with an increased mean clade-specific evolutionary rate, and neither Pneumoridae nor the monophyletic group consisting of Acridinae, Gomphocerinae and Oedipodinae showed any rate shifts. Rate shifts are usually associated with key innovations leading to increased diversification rates^39^, which would indicate that the evolution of sexual communication using acoustic signalling was not necessarily the major key innovation for all of these singing lineages. It is conceivable that both Tettigoniidae and Pamphagidae did experience the increased diversification rates due to their evolution of acoustic signalling, which is the most widespread and dominant mode of communication in these lineages^35,47^ and other forms of signalling (visual or chemical) are not known. However, it is also possible that, at least for Tettigoniidae, there could have been other key innovations, such as impressive leaf masquerade and diverse feeding habits^91^, that might have led to the rate shift possibly related to the contemporaneous rise of angiosperms. These findings are bolstered by a more direct analysis of trait-dependent diversification using HiSSE^42^. Regardless of models used, the lineages that evolved hearing and sound-producing organs, as well as the lineages with confirmed acoustic communication had higher net diversification rates. However, when the multimodel inference method was used, the best-fitting models collectively suggest that the evolution of hearing organs affected the net diversification rate, but both the evolution of sound-producing organs and the evolution acoustic communication were independent of the diversification processes, and thus did not affect the net diversification rate. Therefore, our study finds a pattern consistent with what was shown in tetrapods^4^ in that we find little evidence that acoustic communication alone increased net diversification. Our results have a major implication in enhancing our understanding of signal sender-receiver co-evolution and diversification in Orthoptera, revealing more general insights about the evolution and mechanisms of animal communication.

## Methods

### Phylogenomic analyses and divergence time estimation

Our taxon sampling consisted of 239 species of Orthoptera and 10 polyneopteran outgroups, totalling 249 species. Together, these data represented all 16 superfamilies and 36 families of extant Orthoptera. We included 60 transcriptomes, of which 39 orthopteran species were newly generated either by the 1K Insect Transcriptome Evolution (1KITE) consortium or by the Song Lab at Texas A&M University. The remaining 21 transcriptomes (11 orthopteran and 10 polyneopteran) were from the previous publications (see Supplementary Methods 1.1). To increase taxon sampling, we then combined the transcriptome data with 169 previously and 80 newly generated mtgenomes from 249 taxa. RNA extraction, cDNA library preparation and transcriptome sequencing and assembly were performed within the 1KITE project using the protocols detailed in Supplementary Methods 1.2 and 1.5. Protocols used for the Song Lab samples, as well as for mtgenome data generation are detailed in Supplementary Methods 1.3 and 1.4. A detailed list of all species, including their collection data and National Center for Biotechnology Information (NCBI) accession numbers, is presented in Supplementary Data 1 and 2.

For the transcriptome data, a custom-made orthologous gene set was designed with OrthoDB v7 (ref. ^92^) using four hemimetabolous (*Zootermopsis nevadensis, Pediculus humanus, Acyrthosiphon pisum* and *Rhodnius prolixus*) and one holometabolous (*Nasonia vitripennis*) species, which resulted in 5414 protein-coding genes. We used Orthograph v0.5.3 (ref. ^93^) to generate a profile hidden Markov model (pHMM) from the amino-acid sequences of each reference gene, which was used to search for ortholog candidates in transcript libraries. Orthograph ran protein BLAST (blastp) search using the translated query protein sequences against a database of all amino-acid sequences from all the reference orthologous genes/groups (OGs). For each pHMM hit transcript, the corresponding BLAST result was checked whether the best hit sequence belonged to the OG that the pHMM is based on. Only if the sequence matched, the best-reciprocal hit criterion was fulfilled and the OG was extended with the candidate transcript. Using these methods, we identified on average 3700 OGs. The amino-acid sequences of these OGs were individually aligned on amino-acid level using MAFFT v7.130b^94^ with the L-INS-i algorithm, and the quality of the multiple sequence alignment (MSA) was checked using the pipeline described in Supplementary Methods 1.5.

For downstream phylogenetic analyses, we considered regions identified as protein clans, families, single domains or non-annotated regions as evolutionary units in the partitioned analyses. The methods for identifying these evolutionary units are detailed in Supplementary Methods 1.5. Using custom Perl scripts, the results from the protein domain identification step and the identified randomised MSA sections were merged into a masked supermatrix. The total alignment length spanned 1,647,472 amino-acid positions, and a back-translated nucleotide supermatrix was created using several custom-made Perl scripts. MARE v0.1.2-rc^95^ was used to assess the information content (IC) of each data block, and all identified data blocks showing an information content of 0 (IC = 0) were removed from the supermatrices. From these data, we created four transcriptome data sets: (i) D_aa,trans,complete_, 1,541,865 aligned amino acids with 1743 domain-based metapartitions; (ii) D_aa,trans,strict_, 436,488 aligned amino acids with 102 metapartitions with 100% matrix saturation; (iii) D_nt,trans,complete_, a corresponding data set of D_aa,trans,comple_, comprising 1,541,865 aligned sites of second codon positions only; and (iv) D_nt,trans,strict_, a corresponding data set of D_aa,trans,strict_, comprising 436,488 aligned sites of second codon positions only. In order to select the most appropriate number of partitions for these data sets, we used PartitionFinder 2.0 (ref. ^96^) in combination with the provided RAxML version. For the mtgenome data, we created a concatenated matrix of nucleotide sequences consisting of 13 protein-coding genes aligned based on the conservation of reading frames using MUSCLE^97^ and divided the data matrix into a total of 39 data blocks (13 mitochondrial protein-coding genes divided into individual codon positions). We used PartitionFinder to search for the best-fit scheme as well as to estimate the model of nucleotide evolution for each partition. We then combined the transcriptome data with the mtgenome data by concatenating the D_nt,trans,complete_ and D_nt,strans,strict_ data sets each with the aligned matrix of mtgenomes of 249 taxa, 60 of which overlapped with the transcriptome data: (v) D_nt,trans+mito,complete_, comprising 1,554,238 aligned sites of nucleotides with 1766 metapartitions; and (vi) D_nt,trans+mito,strict_, comprising 448,861 aligned sites of nucleotides with 125 metapartitions. Additional details on data set preparation are presented in Supplementary Methods 1.5.

We analysed these six data sets in a maximum likelihood framework using IQ-TREE v1.5.4 (ref. ^98^) with the best-scoring substitution matrix for each partition. We performed 50 independent tree searches for each data set and node support was estimated via non-parametric bootstrapping of 100 bootstraps replicates in IQ-TREE and mapped onto the ML tree with the best log-likelihood. We also determined support for specific phylogenetic relationships using four-cluster likelihood mapping (FcLM)^33^ by selecting incongruent nodes based on the tree inferences of the six data sets and additionally checking for confounding signal due to among-lineage heterogeneity, non-random substitution processes and/or distribution of missing data with permuted data sets with phylogenetic signal destroyed.

To estimate divergence times, we first conducted a thorough review of available fossils to identify potential calibration points (see Supplementary Methods 2). We applied a rigorous set of criteria to select the most reliable ones. In total, we included 5 polyneopteran and 6 orthopteran fossils to time-calibrate for the analysis. All the calibrations, including the root age, were set to soft maximum bound at 412 million years ago (the oldest age of Rhynie Chert^99^) using uniform priors. We estimated divergence times using MCMCTree implemented in the software package PAML v.4.9 (ref. ^100^) based on the modified matrix of the D_aa,trans,strict_ data set as it represented the most decisive data set. This modified matrix containing only sites with unambiguous data for at least 80% of the 60 taxa was necessary to overcome computational limitations when estimating node ages resulting from the large size of the data set. Previous studies have shown that results of dating analysis are robust to missing data patterns and this data set reduction^101^. In addition, to further reduce computational effort, we chose an unpartitioned dating analysis. We set the model LG (aaRatefile = lg.dat) + G with 5 rate categories, empirically estimated base frequencies (model = 2) and allowed rates to be inferred from individual sites (RateAncestor = 1). We conducted Hessian matrix calculations according to the above specifications with CODEML as implemented in PAML using empirical +F base frequencies estimated from the respective data set. MCMC chains ran for 1,000,000 generations (sfreq = 50) while discarding a burn-in of 100,000 generations. A total of four independent runs were done at the University of Memphis HPC cluster and using Texas A&M HPC cluster. Additional details on phylogenetic analysis, topology testing, and divergence time estimate analysis are presented in Supplementary Methods 1.6, 1.7, and 1.8.

### Phylogenetic comparative methods

To trace the evolution of hearing and sound-producing organs along the phylogeny, we first conducted a thorough literature review and physical examination of the specimens to characterise these organs in all species included in this study. For hearing organs, we coded whether tympanum was absent, present on thorax (Mantidae), on fore tibiae (Ensifera) or on abdomen (Caelifera). We also included atympanate hearing found in Pneumoridae as one of the states. For sound-producing organs, we used a specific naming convention in which the first-named structure has the stridulatory file and the second named structure has the scraper. For example, abdominal-femoral stridulation would have the stridulatory files on the abdomen and the scraper on the inner side of hind femora. The possible combinations used were: absent, tegmino-pronotal, tegmino-femoral, tegmino-alary, tegmino-tegminal, abdomino-alary, abdomino-femoral, Krauss’s organ-femoral and femoro-tegminal stridulation. In addition, we included another type of sound-producing mechanism, only found in Acrididae, known as crepitation^36^, which produces sound by snapping wings when grasshoppers fold and unfold. The complete list of characters used for this analysis is presented in Supplementary Data File 14.

We performed ancestral character state reconstruction of hearing and sound-producing organs in a maximum likelihood framework using the topology resulting from the D_nt,trans+mito,strict_. We fitted a continuous-time Markov chain (Mk) single-rate (ER) model to our data to infer character evolution using the R package *phytools*^102^. Using the same data set, we also performed Pagel’s^37^ binary character correlation test for the evolutionary correlation between hearing and sound production, using *phytools*. We pruned the phylogenetic tree to create Orthoptera-only, Ensifera-only and Caelifera-only data sets to compare and contrast lineage-specific patterns. We recoded different types of tympanal and stridulatory mechanisms as simple presence-absence binary characters for both hearing and sound production to reveal the general co-evolutionary dynamics of these two traits. For each data set, we fitted four models of co-evolution between hearing and sound production and compared the results using the Akaike Information Criterion (AIC): (i) hearing and sound production evolve independently; (ii) the evolution of hearing depends on the evolution of sound production; (iii) the evolution of sound production depends on the evolution of hearing; and (iv) hearing and sound production evolve interdependently.

For Ensifera, we also examined the evolution of the complex tibial organ in the forelegs. Although detailed neuroanatomical studies of auditory sensory organs are limited to only a small number of species^7,68^, it has been suggested that the complex tibial organ consisting of the subgenual organ (SGO) and the intermediate organ (IO) was the ancestral condition in Ensifera^7^. Extant Grylloidea and Gryllotalpidae have the sensory organ consisting of the SGO and the tympanal organ (TO), which is presumed to be modified from the IO as the auditory receptor cells^7^. By contrast, Tettigoniidae and Prophalangopsidae have the sensory organ consisting of the SGO, the IO, and the sensory neurons in the crista acustica (CA) responding to the auditory signal^7^. Atympanate ensiferans are also known to vary in terms of the configuration of the complex tibial organ. Rhaphidophoridae have the SGO and the IO, with no obvious trace of specialised auditory receptor cells^103^. Schizodactylidae, Gryllacrididae, Stenopelmatidae and Anostostomatidae all have the sensory organ similar to that of Tettigoniidae, consisting of the SGO, the IO and the sensory neurons that are homologous to the CA, but with no auditory specialisation, called the crista acustica homologue (CAH)^69,104^. Because we did not have detailed neuroanatomical data for our taxon sampling, we could not perform ancestral character state reconstruction of the complex tibial organ in Ensifera, but we were able to map this character on the phylogeny based on the assumption that the configuration would be conserved at the taxonomic family level.

To estimate the rates of lineage-specific diversification, we performed a diversification analysis using the program Bayesian analysis of macroevolutionary mixtures (BAMM)^39^ and the R package *BAMMtools*^105^. Because BAMM required a comprehensive time-calibrated ultrametric tree, we performed a divergence time estimate analysis using the 249-taxa D_nt,trans+mito,strict_ data set with the same 11 fossil calibration points using MCMCTree as described in Supplementary Methods 1.8. To accurately represent species diversity and to account for incomplete taxon sampling, we specified sampling fraction for each family based on the number of described species recorded in the Orthoptera Species File^106^. We set priors using setBAMMpriors function in *BAMMtools* before the analysis and modified the default setting to achieve convergence. The priors used for the analysis were expectedNumberOfShifts=1.0; lambdaInitPrior=17.0512659943593; lambdaShiftPrior=0.00279913753644403; muInitPrior=17.0512659943593; lambdaIsTimeVariablePrior=0. We used “speciationextinction” as a model for the diversification analysis in BAMM, and ran for 10 million generations with a sampling frequency of 1000. Convergence assessment, analysis of rate shifts, and calculation of clade-specific rates were performed using *BAMMtools*.

To test whether the evolution of hearing and sound production has affected speciation and extinction rates, we fitted models of trait-dependent diversification using the R package *hisse*^42^. Because it has been shown that the presence of unmeasured factors (or hidden states) could impact estimation of diversification rates for any observed trait when analysed under the framework of BiSSE (Binary State Speciation and Extinction) methods^107^, we adopted a multimodel inference method, implemented in HiSSE (Hidden State Speciation and Extinction)^42^. We first pruned the time-calibrated ultrametric tree to only include Orthoptera (239 terminals), and used the binary character data sets for hearing and sound-producing organs previously used for the Pagel’s test. Because the presence of these organs does not necessarily indicate the presence of acoustic communication, we created an additional data set to code acoustic communication as a binary character to test if its evolution affected the diversification rate. We fitted 24 different models, used in Beaulieu and O’Meara^42^, to both the hearing data set and the sound production data set for Orthoptera. These models included four models corresponding to BiSSE models, four models corresponding to trait-independent models (described as CID models), and 16 models corresponding to different HiSSE models that assumed a hidden state associated with both the observed states. Detailed descriptions of these models are included in Supplementary Methods 3.4. For all cases, we included the sampling fraction for the observed states in the models by calculating the proportion of the known 0’s (i.e. absence) represented and the proportion of the known 1’s (i.e. presence) represented in our tree. The resulting models were compared using the AIC. All analyses were carried out in *hisse*.

### Reporting summary

Further information on research design is available in the Nature Research Reporting Summary linked to this article.

## Supplementary information

Supplementary Information

Peer Review

Reporting Summary

Description of Additional Supplementary Files

Supplementary Data 1-15

## Data Availability

All data sets generated and/or analysed during this study, including those related to phylogenomic analyses, divergence time estimation, and character evolution analyses, have been published in Dryad Digital Repository [10.5061/dryad.qjq2bvqc6]. All transcriptome and mitochondrial genome data, both newly sequenced and previously published, can be accessed at NCBI databases and specific accession numbers with hyperlinks can be found in Supplementary Data 1 and 3. Furthermore, additional information related to methods and analyses is included in Supplementary Information and Supplementary Data.

## Code availability

All scripts for the phylogenomic pipeline, including alignment refinement, protein domain identification, alignment masking, optimizing data sets and topological tests haven been previously published [10.1126/science.1257570] and can be accessed [https://www.zfmk.de/en/research/projects/1kite-the-evolution-of-the-insects]. In addition, we used following published custom code: AliCUT v2.3 [https://github.com/PatrickKueck/AliCUT] SymTest v.2.0.47 [https://github.com/ottmi/symtest], MitoZ [https://github.com/linzhi2013/MitoZ], selectSites.pl [https://github.com/justincbagley/MAGNET/blob/master/perl/selectSites.pl], pfam_scan.pl [http://ftp.ebi.ac.uk/pub/databases/Pfam/Tools/] and plot_runs_MCMCtree.R [https://github.com/kmeusemann/scripts/blob/master/plot_runs_MCMCtree.R].
