# Author Correction: Phylogenomic analysis sheds light on the evolutionary pathways towards acoustic communication in Orthoptera

**DOI:** 10.1038/s41467-020-19626-8

**Published:** 2020-11-03

**Authors:** Hojun Song, Olivier Béthoux, Seunggwan Shin, Alexander Donath, Harald Letsch, Shanlin Liu, Duane D. McKenna, Guanliang Meng, Bernhard Misof, Lars Podsiadlowski, Xin Zhou, Benjamin Wipfler, Sabrina Simon

**Affiliations:** 1 Department of Entomology, Texas A&M University, College Station, TX 77843 USA; 2 CR2P (Centre de Recherche en Paléontologie – Paris), MNHN – CNRS – Sorbonne Université, Muséum National d’Histoire Naturelle, 75005 Paris, France; 3 Department of Biological Sciences and Center for Biodiversity Research, University of Memphis, Memphis, TN 38152 USA; 4 School of Biological Sciences, Seoul National University, Seoul, 08826 Republic of Korea; 5 Center for Molecular Biodiversity Research (ZMB), Zoological Research Museum Alexander Koenig (ZFMK), 53113 Bonn, Germany; 6 Department für Botanik und Biodiversitätsforschung, Universität Wien, 1030 Vienna, Austria; 7 China National GeneBank, BGI-Shenzhen, 518083 Guangdong, China; 8 Department of Entomology, College of Plant Protection, China Agricultural University, 100193 Beijing, China; 9 Institut für Spezielle Zoologie und Evolutionsbiologie, Friedrich-Schiller-University Jena, 07743 Jena, Germany; 10 Center of Taxonomy and Evolutionary Research, Zoological Research Museum Alexander Koenig, 53113 Bonn, Germany; 11 Biosystematics Group, Wageningen University and Research, 6708 PB Wageningen, Netherlands

**Keywords:** Phylogenetics, Sexual selection, Animal behaviour, Entomology

Correction to: *Nature Communications* 10.1038/s41467-020-18739-4, published online 2 October 2020.

The original version of this Article contained an error in Fig. 3, in which the types of tegmino-tegminal stridulation in Gryllidea and in Tettigonioidea shown on the right side of the phylogeny were reversed. The correct version of Fig. 3 is:

which replaces the previous incorrect version.

The original version of this Article contained an error in the last sentence of the Fig. 3 legend, which incorrectly read ‘The common ancestor of Gryllidea evolved “left-over-right” stridulation, the common ancestor of Hagloidea evolved “ambidextrous” stridulation, and the common ancestor of Tettigonioidea evolved “right-over-left” stridulation’. The correct version replaces this sentence with ‘The common ancestor of Gryllidea evolved “right-over-left” stridulation, the common ancestor of Hagloidea evolved “ambidextrous” stridulation, and the common ancestor of Tettigonioidea evolved “left-over-right” stridulation’.

The original version of this Article contained an error in the Discussion, which incorrectly read ‘Crickets and mole crickets stridulate by moving the left forewing over the right, and katydids stridulate in the opposite way by moving the right forewing over the left^43^’. The correct version replaces this sentence with ‘Crickets and mole crickets stridulate by moving the right forewing over the left, and katydids stridulate in the opposite way by moving the left forewing over the right^43^’.

This has been corrected in both the PDF and HTML versions of the Article.

