# Supplementary material for: Phylogenomic analysis sheds light on the evolutionary pathways towards acoustic communication in Orthoptera
Source: Nat Commun. 2020 Oct 2;11:4939. doi: 10.1038/s41467-020-18739-4 (PMC7532154; doi:10.1038/s41467-020-18739-4)
Supplement: Supplementary file 1 — Supplementary Information [file 41467_2020_18739_MOESM1_ESM.pdf]

Supplementary Information

Phylogenomic analysis sheds light on the evolutionary pathways towards acoustic communication in Orthoptera

Hojun Song, Olivier Béthoux, Seunggwan Shin, Alexander Donath, Harald Letsch, Shanlin Liu, Duane McKenna, Guanliang Meng, Bernhard Misof, Lars Podsiadlowski, Xin Zhou, Benjamin Wipfler, and Sabrina Simon

Table of Contents

SUPPLEMENTARY METHODS: DATASETS AND MOLECULAR ANALYSES 2

Taxon Sampling – Transcriptome 2
Transcriptome sequencing & assembly....................................2
Taxon Sampling – Mitochondrial genome....................................2
Mitochondrial genome assembly & annotation....................................2
Dataset generation 3
Phylogenetic inference 5
Topology tests: Four-cluster Likelihood Mapping (FcLM) 6
Divergence time estimation....................................12
Discussion on recovered topology and divergence time estimates 12

SUPPLEMENTARY METHODS: FOSSIL SELECTION 15

Selected fossil calibrations 15
Excluded fossil calibrations....................................19
Locality ages....................................21

SUPPLEMENTARY METHODS: CHARACTER EVOLUTION....................................22

Ancestral character state reconstruction....................................22
Pagel's evolutionary correlation analysis....................................24
Diversification analysis 26
Trait-dependent diversification analysis....................................27

SUPPLEMENTARY FIGURES 29

SUPPLEMENTARY NOTE: ARCHIVE FILES AND DESCRIPTIONS PROVIDED VIA THE DIGITAL REPOSITORY DRYAD....................................43

SUPPLEMENTARY REFERENCES....................................45

Supplementary Methods: Datasets and molecular analyses

Taxon Sampling – Transcriptome

We sampled RNA of 39 Orthoptera species, of which 30 have been collected within the 1KITE initiative (https://www.1kite.org/) and 9 have been generated by the Song lab (indicated by * in Supplementary Table 1.1). We also included in our study transcriptomes of 21 already sequenced species (11 Orthoptera and 10 outgroup species) published by us in two other investigations[1,2]. Our sampling of transcriptomes thus comprised 50 samples of Orthoptera and 10 outgroup taxa, representing all other Polyneoptera lineages. Specimens were either preserved in liquid nitrogen, then kept frozen at -35°C or directly grounded in RNAlater and kept at 4°C until further processing. Details on all samples used for sequencing are provided in Supplementary Table 1.1 and can be found at the National Center for Biotechnology Information (NCBI) (e.g., sex, collection date, collector, etc.) under the 1KITE umbrella project and / or the respective BioSample number (Supplementary Table 1.1).

Transcriptome sequencing & assembly

For all transcriptomes generated within the 1KITE initiative, RNA extraction and cDNA library preparation, and transcriptome sequencing were conducted at the Beijing Genomics Institute (BGI) Shenzhen and are described in detail in Peters et al.[3]. These samples were all sequenced on the HiSeq 2000 platform with 150 bp paired-end (PE) reads, generating approximately 2.5Gb of raw data each. Sequenced raw RNA-Seq reads were assembled using SOAPdenovo-Trans v1.01[4] following the methods described in Peters et al.[3]. Before assembly, the adapter contamination, reads included >10 Ns, and reads included > 50 base pairs of low-quality were sorted out. After filtering steps, we performed a *de novo* assembly for each taxon. Details of the *de novo* assembly were described by Misof et al.[2] and Xie et al.[4]. The 1KITE transcriptome assemblies were checked for vector and linker/adapter contamination using VecScreen (http://www.ncbi.nlm.nih.gov/tools/vecscreen/) and the UniVec database build 7.1 (http://www.ncbi.nlm.nih.gov/tools/vecscreen/univec/) following the protocol of Peters et al.[3]. The cross-contamination check was done by comparison of abundances of high-quality blast hits, as described in detail in Mayer et al.[5]. For details on the number of contigs before and after contamination check, see Supplementary Table 1.2. Submission to NCBI Sequence Read Archive (SRA) and the Transcriptome Shotgun Assembly (TSA) database were conducted as described in Peters et al.[3]

For the nine species sequenced by Song lab (indicated by * in Supplementary Table 1.1), RNA was extracted using a Trizol-chloroform extraction, followed by clean-up with a RNeasy mini kit using an on-column DNAse treatment with an RNAse-free DNAse set (Thermofisher Scientific, Waltham, MA, and Qiagen, Valencia, CA). RNA concentrations were measured with a spectrophotometer (DS-11, DeNovix, Wilmington, DE), and RNA integrity was analyzed with a Fragment Analyzer (Agilent Technologies, Ankeny, IA). Library preparation, sequencing and pre-processing steps up to demultiplexing base call files were all performed at Texas A&M's AgriLife Research Genomics and Bioinformatics Service. For library preparation, Illumina's TruSeq Stranded Total RNA Library Prep Kit was used and paired-end sequencing (125 bp) was performed using 1 lane on an Illumina HiSeq2500 (San Diego, CASequence). For further processing, raw reads were imported into a personalized Galaxy environment[6] on a supercomputing cluster of the High-Performance Research Computing group of Texas A&M University (Ada, https://hprc.tamu.edu) for trimming and quality check. The filtered reads were used for *de novo* transcriptome assembly using Trinity v2.2.0[7]; default settings, in silico normalization using the default value of 50 as max read coverage enabled). The quality of these final transcriptomes was analyzed with Trinitystats[7] and the fraction of reads mapping back to their transcriptome was obtained with bowtie2[8,9] in the preset mode 'very sensitive, end-to-end' and flagstat[10,11].

Taxon Sampling – Mitochondrial genome

In total, we sampled for 249 species the complete mitochondrial genome (Supplementary Table 2). For this study, we generated 69 new mitochondrial genomes, all of which were generated using high-throughput-sequencing (HTS) technologies. For 63 species, we extracted 13 mitochondrial protein-coding genes from the assembled transcriptomes. Of these, 60 were the corresponding species of the used transcriptome taxa (indicated by # in Supplementary Table 2) and for the remaining three species (*Gymnoscirtetes pusillus*, *Conocephalus dorsalis*, and *Xyronotus azteca*) which we sequenced transcriptomes for, we only used the mitochondrial genome data. For six species, we performed shotgun sequencing of genomic DNA using the Illumina platform as described in Song et al.[12]. We further added 180 previously published Orthoptera mitochondrial genomes[13-81].

Mitochondrial genome assembly & annotation

Mitochondrial genomes were assembled from high-throughput-sequencing (HTS) data from the 1KITE project with a beta version of MitoZ[82]. Briefly, MitoZ adopts all the algorithms in SOAPdenovo-Trans[4] but for the scaffolding step we ask for higher linkage support to avoid connections between mitochondrial reads and NUMTs and improve accuracy. Then, MitoZ uses a Hidden Markov Model (HMM)[83] based method[84] to filter out candidate

mitogenome sequences, and further removes potential false positive mitochondrial scaffold, such as nuclear mitochondrial DNA segments (NUMTs) and contaminations based on species annotation and sequence confident. In the species annotation step, the protein coding genes (PCG) of each candidate mitochondrial sequence were first annotated with an in-house Perl script (explained with more details in subsequent mitochondrial genome annotation step) and then assigned to a taxonomy lineage. Only the candidate mitochondrial sequences which belonged to a user defined clade (e.g. order, family) were retained for further analyses. Then a confident score for each sequence was calculated based on sequence assembly reliability (output by SOAPdenovo-Trans) and PCG number and its completeness. Sequences were ranked by their confident scores and MitoZ tries to find all 13 PCGs with the highest confident score. tBlastn v2.2.19[85] was used to align mitochondrial sequences to a local protein sequences database and get the candidate PCG sequences. Afterwards Genewise v2.2.0[86] was used to determine the boundaries of each PCG. A covariance model (CM)[87] based method, MiTFi [88], was used to perform tRNA annotation for mitochondrial sequences. For the rRNA annotation, MitoZ with the global searching mode implemented in infernal-1.1.1[89] was used. In case no candidates were detected, the local searching mode was used. To achieve more comprehensive results, we ran MitoZ with different kmer sizes (-K 29, -K 41, -K 71) separately and combined the annotated results output from MitoZ.
From the nine transcriptomes generated by Song Lab, we extracted transcripts of mitochondrial protein-coding genes using “Map to read” function in Geneious 10 (Biomatters, Ltd.) with a complete mitochondrial genome of *Acrida willemsei* (NC_011303) as a reference. The resulting contigs were manually inspected to identify start and stop codons of each protein-coding gene.

Dataset generation

Transcriptomic data: ortholog prediction

A custom-made orthologous gene set was designed from the public database OrthoDB v7[90-92] as recommended in Orthograph manual[93]. We selected clustering orthologous gene IDs for the node Insecta on OrthoDB v7 and listed single copy genes across five reference taxa from Blattodea (*Zootermopsis nevadensis*), Phthiraptera (*Pediculus humanus*), two Hemiptera (*Acyrthosiphon pisum*, *Rhodnius prolixus*), and Hymenoptera (*Nasonia vitripennis*). Unfortunately, the Orthoptera genome has been poorly studied, so we could not include any Orthopteran reference genome for this Cluster of Orthologous Genes (COG) set. The reference genomes were selected based on annotation and sequence quality. After collecting gene IDs for this COG set, we found that each combination of three taxa including Isoptera could generate an additional number of reference gene set which has only one of four taxa (except Isoptera) have the multicopy or missing genes. The closest relative genome available for the Orthoptera is Isoptera in the OrthoDB v7. Thus, we made four combinations that excluded one of four taxa (except Isoptera) respectively and then ran OrthoDB v7 single copy only search for each set, using the same option. We extracted four sets of reference species gene set IDs information regarding orthologous gene EOG numbers from OrthoDB v7. These tables generated by OrthoDB v7 were concatenated to remove duplicated gene IDs from later four taxa sets. The official gene sets were downloaded from OrthoDB v7 (ftp://cegg.unige.ch/OrthoDB7/FASTA/) and the sequence IDs were confirmed matching with the reference table. Following official gene sets were used for this bioinformatics pipeline, *Acyrthosiphon pisum* (International Aphid Genomics 2010), *Pediculus humanus*[94], *Rhodnius prolixus*[95], *Zootermopsis nevadensis*[96], *Nasonia vitripennis*[97]. Finally, the resulting ortholog set comprised 5,414 protein-coding genes.
We used Orthograph v0.5.3[93] to generate a profile hidden Markov model (pHMM) from the amino acid sequences of each reference gene (i.e., ortholog group (OG) equivalent to OrthoDB EOG IDs, containing the orthologous amino acid sequences of the five reference species). These pHMMs were used to search (at the translational level) for ortholog candidates in transcript libraries. Therefore, Orthograph converted the nucleotide sequences to the protein level with six open reading frame (ORF) using Exonerate[98]. After that, Orthograph ran protein BLAST (blastp) search using the translated query protein sequences against a database of all amino acid sequences from all the reference OGs. Both, pHMM and BLAST search results were stored in a database for orthology prediction at the later step. For each pHMM hit transcript, the corresponding BLAST result was checked whether the best hit sequence belonged to the OG that the pHMM is based on. Only if the sequence matched, the best-reciprocal hit (BRH) criterion was fulfilled and the OG was extended with the candidate transcript. Orthograph was applied with non-strict reciprocal searches (best reciprocal hit (BRH) criterion being fulfilled if the reciprocal BLAST search found in at least one of the five reference taxa the candidate ortholog sequence as best hit) and keeping all parameters as default.
Using these settings, we identified on average 3,700 orthologous genes/groups (OGs) (minimum: 806 OGs identified in *Psedna nana*, maximum 4,631 OGs identified in *Stenacris vitreipennis*), see Supplementary Table 3 for Orthograph results.

Transcriptomic data: alignment, protein domain identification, alignment masking, optimizing datasets, partitioning, and substitution model selection

Orthograph results were summarized according to each OG with the script summarize_orthograph_results.pl provided with the Orthograph package (-m option). This resulted into a total of 4,986 summarized OGs. The

sequences of retrieved OGs were aligned on amino-acid level (aa) using MAFFT v7.130b[99] with the L-INS-i algorithm. Each aa multiple sequence alignment (MSA) was subsequently checked for the presence of outliers as described in Misof et al.[2]. Although the MAFFT generated alignment and checked traditional way, in case of the NGS *de novo* assembled sequences could have possible misalignment due to the different region of the gene could generate unpredictable match (if there was no region overlapping). Thus, we assessed the quality of MSA by bioinformatics script(s) (updates available upon request), which can identify outlier genes following recent phylogenomics study[3]. All possible problematic outlier sequences were removed from the MSA set. The nucleotide set was handled the same filtering with amino acid alignments that all regarding outliers were removed at this stage. We further removed sequences from all five reference species (*Acyrthosiphon pisum*, *Pediculus humanus*, *Rhodnius prolixus*, *Zootermopsis nevadensis*, *Nasonia vitripennis*) from the amino-acid MSAs and nucleotide OGs. Resulting gap only columns in the aa MSAs due to removal of sequences were removed as well. We then generated MSAs of nucleotides corresponding to the aa MSAs with a modified version of the software PAL2NAL [100] using the corresponding amino-acid MSAs as blueprint.

For downstream phylogenetic analyses, we considered regions identified as protein clans, families, single domains or non-annotated regions (so called voids) as evolutionary units in the partitioned analyses. For a rationale see Misof et al.[2]. Using the Pfam database[101] release 28.0 (including only the Pfam-A database) in conjunction with the software pfam_scan.pl v1.5 and HMMER[102], Domain-identification-v1.3 and Domain-parser-v1.4.1-dist, we identified 9,937 Pfam-A domains, and 13,968 void regions using the same strategy as described in Wipfler et al.[1]. In parallel to the protein domain identification, a modified version of Aliscore v1.2[103,104] was used to identify blocks of putative alignment ambiguities and randomized MSA sections within each aa MSA separately using the default sliding window size, the –r option in order to compare all sequence pairs in each sliding window and a special scoring for gappy aa data (option –e). Using AliCUT v2.3[105] (https://github.com/PatrickKueck/AliCUT) putative alignment ambiguities or randomized alignments were removed from both aa and nt MSAs.

Using custom Perl scripts (updates available upon request), the results from the protein domain identification step and the identified randomized MSA sections were merged into a masked supermatrix. Thus, the resulting supermatrix consists of data blocks with regions of putative alignment sections removed. The total alignment length spanned 1,647,472 amino-acid (aa) positions. A nucleotide (nt) supermatrix that exactly corresponds to the amino-acid supermatrix was created using several custom-made Perl scripts (updates available upon request), resulting in a total alignment length of 4,942,416 nucleotide positions. We further used MARE v0.1.2-rc[106] to assess the information content (IC) of each data block (domains, domains assigned to a clan and voids) on the translational level. All identified data blocks showing an information content of 0 (IC=0) were removed from the aa supermatrix and correspondingly from the nt supermatrix. This resulted in the final amino-acid complete transcriptome only datasets $D_{aa,trans,complete}$ (60 taxa, 1,541,865 aa, 5,069 initial data blocks), see also Supplementary Table 4 for overview of datasets. For the corresponding nt dataset we evaluated whether the nt complete transcriptome only dataset have evolved under globally stationary, reversible and homogeneous (SRH) conditions. Therefore, we applied SymTest v.2.0.47 (https://github.com/ottmi/symtest), which uses three matched-pairs tests of homogeneity; further details are provided in Misof et al.[2]. The tests were applied on the 1st only, 2nd only, 3rd only, 1st + 2nd only and all codon positions dataset. We generated heat maps based on the p-values obtained from Bowker's matched-pairs test of symmetry in order to determine which sequence pairs could be assumed to have evolved under globally SRH conditions (Supplementary Figure 1). Further downstream analyses were performed on the transcriptome nucleotide dataset with the 2nd codon positions only, $D_{nt,trans,complete}$ (60 taxa, 1,541,865 nt, 5,069 initial data blocks), as this showed a smaller heterogeneity compared to the other datasets consisting of either only the 1st or 3rd codon positions or consisting of 1st + 2nd or all codon positions (Supplementary Figure 1). In order to reduce the amount of missing data in these two datasets ($D_{aa,trans,complete}$ and $D_{nt,trans,complete}$), we generated two additional transcriptome only datasets, one on the translational and one on the transcriptional level using again only the 2nd codon positions. We increased data coverage to 100% matrix saturation by including only data blocks, i.e. that contained sequence information for each taxon in our dataset. This resulted in strict datasets: $D_{aa,trans,strict}$ (60 taxa, 436,488 aa, 137 initial data blocks), $D_{nt,trans,strict}$ (60 taxa, 436,488 nt, 137 initial data blocks), see Supplementary Table 4 for overview of datasets.

To further evaluate the coverage of all four transcriptome only datasets with respect to pairwise sequence coverage of (missing) data, we used AliStat v1.6[107], see also Misof et al.[2], and generated the respective heat map. See Supplementary Table 4 for overview of completeness scores and Supplementary Figures S2A-D for corresponding heatmaps.

In order to select the most appropriate number of partitions (i.e., merged data blocks), we used PartitionFinder 2.0.0 (prerelease 17)[108] in combination with the provided RAxML version. Because we used protein domains as an evolutionary unit, we first used the two amino-acid datasets: 1) data blocks with IC=0 removed: $D_{aa,trans,complete}$ (60 taxa, 1,541,865 aa, 5,069 initial data blocks), and 2) data blocks with IC=0 removed and 100% matrix saturation: $D_{aa,trans,strict}$ (60 taxa, 436,488 aa, 137 initial data blocks), to merge data blocks and to select the optimal partitioning scheme in PartitionFinder (options --rclusterf --rcluster-max 11000 (--rcluster-max 1000 for $D_{aa,trans,strict}$) --rcluster-percent 100 -q -p 28 --weights 1,1,0,1 -v --all-states --min-subset-size 100). This

partitioning scheme search (merging data blocks) resulted in 1,743 metapartitions for $D_{aa,trans,complete}$ and 102 metapartitions for $D_{aa,trans,strict}$, respectively. Please note that the boundaries of the metapartitions identified on translational level for the two aa datasets are equivalent to the boundaries we kept for the corresponding two nucleotide datasets (see below). For the two aa datasets, we further restricted the PartitionFinder search to eleven amino-acid substitution models as these are the most selected models for empirical studies on Hexapoda[2,3,109], namely LG+G, WAG+G, DCMUT+G, JTT+G, BLOSUM62+G, LG+G+F, WAG+G+F, DCMUT+G+F, JTT+G+F, BLOSUM62+G+F, LG4X[110-117]. To select the best substitution model for each of these metapartitions for the two nucleotide datasets ($D_{nt,trans,complete}$ and $D_{nt,trans,strict}$), we applied ModelFinder as implemented in IQ-TREE v1.5.4[118,119]; options –m MF –gmedian. The best model for each partition was selected according to the Bayesian information criterion (BIC). All four alignments and the corresponding partitioning scheme with the selected substitution models are deposited on Dryad (https://doi.org/10.5061/dryad.qjq2bvqc6).

Mitochondrial data: alignment, alignment masking, optimizing datasets, partitioning, and substitution model selection

We first created 13 individual mitochondrial protein-coding gene datasets from all 249 species. For some species, certain mitochondrial protein-coding genes were not properly sequenced, and thus, the total number of species included for each mitochondrial gene ranged from 231 species (ND6 gene) to 249 species (ND1 gene). We aligned each gene based on the conservation of reading frames by first translating into amino acids, aligning individually in MUSCLE[120] using default parameters, and back-translating into nucleotide sequences in Geneious. All these individual alignments were concatenated into a single matrix using SequenceMatrix[121]. We divided the data into a total of 39 data blocks (13 mitochondrial protein-coding genes divided into individual codon positions). We then used PartitionFinder v1.1.1[122] using the 'greedy' algorithm (heuristic search) with branch lengths estimated as 'linked' to search for the best-fit scheme as well as to estimate the model of nucleotide evolution for each partition using the Bayesian Information Criterion (BIC).

Creating combined datasets: transcriptomic + mitochondrial data

We used the two nucleotide transcriptome datasets ($D_{nt,trans,complete}$ and $D_{nt,trans,strict}$) comprising 60 taxa to combine them with the mitochondrial dataset, comprising 249 taxa. Please note that the 60 transcriptome taxa are included in the mitochondrial dataset. Consequently, the two combined datasets comprised in total 249 taxa, but now comprising for $D_{nt,trans+mito,complete}$ 1,554,238 nt and 1,766 metapartitions, and for $D_{nt,trans+mito,strict}$ 448,861 nt and 125 metapartitions, see Supplementary Table 4 for overview of datasets. Also for these two combined datasets, the coverage with respect to pairwise sequence coverage of (missing) data was assessed using AliStat v.1.6[107] and generated respective heat map were generated. See Supplementary Table 4 for overview of completeness scores and Supplementary Figures S2E&F for corresponding heatmaps.

Phylogenetic inference

Phylogenetic relationships or all six datasets were inferred under the maximum likelihood (ML) optimality criterion as implemented in IQ-TREE v1.5.4[119,123] and by using the best-scoring substitution matrix for each partition (option –spp). We performed 50 independent tree searches (25 searches with a random and 25 with a parsimony start tree). Node support was estimated via non-parametric bootstrapping of 100 bootstraps replicates in IQ-TREE and mapped onto the ML tree with the best log-likelihood. Inferred phylogenetic relationships are shown in Figures 1&2 and Supplementary Figures S3A-E.

The topology inferred from the maximum likelihood analysis of $D_{aa,trans,complete}$ (Supplementary Figure 3A) was identical to that inferred from $D_{aa,trans,strict}$ (Supplementary Figure 3B) except the position of *Haplotropis brunneriana* (Pamphagidae) such that this species was recovered as basal to the clade consisting of Acridoidea (Romaleidae + Acrididae) and Pyrgomorphoidea (Pyrgomorphidae) in the $D_{aa,trans,complete}$ tree, while it was recovered as sister to sister to the remaining Acridoidea in the $D_{aa,trans,strict}$ tree. Based on morphology, Pamphagidae is classified as Acridoidea, and the topology recovered from the $D_{aa,trans,strict}$ dataset is more congruent with our understanding of the phylogenetic position of Pamphagidae.

$D_{aa,trans,complete}$ (Supplementary Figure 3A) and $D_{nt,trans,complete}$ (Supplementary Figure 3C) resulted in the identical topologies in terms of Caelifera, but the relationships within Ensifera differed between the two. While the $D_{aa,trans,complete}$ tree recovered the first diverging lineage within Ensifera as Gryllidea (Myrmecophilidae, Gryllotalpidae, Gryllidae, Phalangopsidae), the $D_{nt,trans,complete}$ tree recovered Rhaphidophoroidea (Rhaphidophoridae) as the first diverging lineage within Ensifera. Based on morphology and other previous molecular studies, Ensifera has always been considered to consist of two infraorders Gryllidea and Tettigoniidea, and the $D_{aa,trans,complete}$ was more congruent with our current understanding of the relationships. These two datasets also differed in the position of Gryllotalpidae related to other members of Gryllidea. In $D_{aa,trans,complete}$, Gryllotalpidae was sister to Grylloidea (Gryllidae + Phalangopsidae), while in $D_{nt,trans,complete}$, it was basal to other Gryllidea.

$D_{nt,trans,complete}$ (Supplementary Figure 3C) and $D_{nt,trans,strict}$ (Supplementary Figure 3D) resulted in the identical topologies in terms of Ensifera, but differ in the position of Pamphagidae as described above. $D_{nt,trans,complete}$ tree

recovered the same phylogenetic position of Pamphagidae as the $D_{aa,trans,complete}$ tree while the $D_{nt,trans,strict}$ tree recovered the same phylogenetic position of Pamphagidae as the $D_{aa,trans,strict}$ tree.

$D_{nt,trans+mito,complete}$ (Supplementary Figure 3E) and $D_{nt,trans+mito,strict}$ (Supplementary Figure 3F) both recovered congruent topologies except the phylogenetic position of Rhaphidophoridae, which in the $D_{nt,trans+mito,complete}$ tree it was at the base of Ensifera, whereas in the $D_{nt,trans+mito,strict}$ tree, it was at the base of Tettigoniidea. The relationships within Caelifera were mostly congruent between the two datasets.

Overall, the positions of Pamphagidae, Rhaphidophoridae, and Gryllotalpidae were sensitive to the amount of data saturation and to how the data were coded (amino acid vs. nucleotide). Our preferred topologies were from $D_{aa,trans,strict}$ and $D_{nt,trans+mito,strict}$. Therefore, we tested for these topologies using FcLM test (see Section 1.7).

Topology tests: Four-cluster Likelihood Mapping (FcLM)

In addition to the non-parametric bootstrap support, we determined support for specific phylogenetic relationships with the aid of the Four-cluster Likelihood Mapping (FcLM) method from Strimmer & von Haeseler [124]. FcLM only addresses single splits in a tree. Therefore, this approach enables identification of hidden signal for single relationships that may not be seen in ML trees. For the FcLM analyses, we selected incongruent nodes based on the tree inferences of the six datasets (see above) and additionally checked for confounding signal due to among-lineage heterogeneity, non-random substitution processes and/or distribution of missing data using the FcLM approach with permuted datasets with phylogenetic signal destroyed, for a rationale see Simon et al.[125] and Misof et al.[2]. FcLM analyses were performed for all six datasets using IQ-TREE v.1.6.2. As substitution model for the permutated datasets, we used LG or GTR, respectively, for each partition.

For each test, we defined four groups and included only partitions for which at least one representative species of the addressed groups was present. Taxa that did not address a particular hypothesis were discarded from the alignment (see Supplementary Table 5 for included species, group definitions are also described below).

Below we describe the rationale for each phylogenetic relationship being tested within each dataset and discuss the results. The tables below present the proportions of quartets that map into areas respective areas in the 2D-simplex graph. T1: unambiguous support for G1,G2 – G3,G4. T2: unambiguous support for G1,G3 – G2,G4. T3: unambiguous support for G1,G4 – G2,G3. Quartets falling into the areas 1 (T1), 2 (T2) and (T3) 3 are informative. Areas 4 (T12), 5 (T23) and 6 (T13) are partly informative, and area 7, i.e. T* is not informative. See Supplementary Table 5 for definition of the four groups (G1-G4) for each test and each dataset.

a) Position of Rhaphidophoridae within Ensifera

A sistergroup relationship of Rhaphidophoridae to (Schizodactylidae ((Prophalangopsidae + Tettigoniidae) + (Gryllacrididae (Stenopelmatidae + Anostostomatidae))) was supported by the two amino-acid transcriptome datasets $D_{aa,trans,complete}$, $D_{aa,trans,strict}$ and the strict combined (transcriptomes and mitochondrial) nucleotide dataset $D_{nt,trans+mito,strict}$ (see Supplementary Figures S3A,B,F), T1 (G1, G2 – G3, G4). Consequently, Gryllidea, which includes Myrmecophilidae, Gryllotalpidae, Gryllidae, Prophalangopsidae, is placed as sistergroup to all remaining Ensifera. In contrast, the two nucleotide transcriptome datasets $D_{nt,trans,complete}$, $D_{nt,trans,strict}$ and the complete combined (transcriptomes and mitochondrial) nucleotide dataset $D_{nt,trans+mito,complete}$ (see Supplementary Figures S3C,D,E) do not support this placement of Rhaphidophoridae. Instead Gryllidea is placed as sistergroup to (Schizodactylidae ((Prophalangopsidae + Tettigoniidae) + (Gryllacrididae (Stenopelmatidae + Anostostomatidae))) and Rhaphidophoridae is placed as sistergroup to all remaining Ensifera, T3 (G1, G4 - G2, G3).

$D_{aa,trans,complete}$ (T1 preferred in ML tree): 1,521,250 aa, 1,649 metapartitions

G1: Rhaphidophoridae (3), G2: Schizodactylidae, Prophalangopsidae, Tettigoniidae, Gryllacrididae, Stenopelmatidae, Anostostomatidae (9), G3: Gryllidea (4), G4: Caelifera (34)

unique quartets: 3,672

Topology	original (support in %)	permutation I (support in %)	permutation II (support in %)	permutation III (support in %)
T1	89.8	24.5	18.6	30.5
T2	0.2	42.9	43.7	31.4
T3	9.7	26.9	32.5	31.8
T1T2	0.1	1.8	1.4	1.5
T1T3	0.2	1.4	1.2	2.4
T2T3	0	2.5	2.5	2.1
T*	0	0.1	0.1	0.2

$D_{aa,trans,strict}$ (T1 preferred in ML tree): 436,488 aa, 102 metapartitions

G1: Rhaphidophoridae (3), G2: Schizodactylidae, Prophalangopsidae, Tettigoniidae, Gryllacrididae, Stenopelmatidae, Anostostomatidae (9), G3: Gryllidea (4), G4: Caelifera (34)

unique quartets: 3,672

Topology	original (support in %)	permutation I (support in %)	permutation II (support in %)	permutation III (support in %)
T1	91.1	28.1	21.4	30.4
T2	0.1	23.8	38.0	25.8
T3	8.5	32.9	22.0	23.0
T1T2	0	4.3	5.4	7.0
T1T3	0.4	5.7	4.3	6.2
T2T3	0	5.0	7.3	6.2
T*	0	1.1	1.6	1.4

$D_{nt,trans+mito,strict}$ (T1 preferred in ML tree): 457,380 nt, 125 metapartitions
G1: Rhaphidophoridae (6), G2: Schizodactylidae, Prophalangopsidae, Tettigoniidae, Gryllacrididae, Stenopelmatidae, Anostostomatidae (51), G3: Gryllidea (15), G4: Caelifera (161)

unique quartets: 825,930

Topology	original (support in %)	permutation I (support in %)	permutation II (support in %)	permutation III (support in %)
T1	61.0	17.7	15.1	26.7
T2	7.8	21.1	14.8	27.3
T3	26.6	20.4	17.6	27.5
T1T2	1.4	10.1	13.0	5.8
T1T3	2.4	9.5	12.5	5.5
T2T3	0.6	10.2	11.4	5.7
T*	0.3	11.0	15.5	1.5

$D_{nt,trans,complete}$ (T3 preferred in ML tree): 1,502,359 nt, 1,593 metapartitions
G1: Rhaphidophoridae (3), G2: Schizodactylidae, Prophalangopsidae, Tettigoniidae, Gryllacrididae, Stenopelmatidae, Anostostomatidae (9), G3: Gryllidea (4), G4: Caelifera (34)

unique quartets: 3,672

Topology	original (support in %)	permutation I (support in %)	permutation II (support in %)	permutation III (support in %)
T1	84.0	13.4	14.0	33.1
T2	0.2	48.2	47.8	29.6
T3	15.3	33.9	32.7	33.5
T1T2	0	1.4	1.6	1.5
T1T3	0.5	0.6	1.0	1.3
T2T3	0	2.4	2.8	0.9
T*	0	0.1	0.1	0.1

$D_{nt,trans,strict}$ (T3 preferred in ML tree): 436,488 nt, 102 metapartitions
G1: Rhaphidophoridae (3), G2: Schizodactylidae, Prophalangopsidae, Tettigoniidae, Gryllacrididae, Stenopelmatidae, Anostostomatidae (9), G3: Gryllidea (4), G4: Caelifera (34)

unique quartets: 3,672

Topology	original (support in %)	permutation I (support in %)	permutation II (support in %)	permutation III (support in %)
T1	83.5	17.8	21.5	27.2
T2	0.1	33.9	30.6	25.2
T3	15.1	29.2	29.2	27.3
T1T2	0	5.7	6.2	6.8
T1T3	1.3	4.4	4.4	6.3
T2T3	0.1	7.6	6.7	5.4
T*	0	1.5	1.3	1.9

$D_{nt,trans+mito,complete}$ (T3 preferred in ML tree): 1,523,251 nt, 1,616 metapartitions
G1: Rhaphidophoridae (6), G2: Schizodactylidae, Prophalangopsidae, Tettigoniidae, Gryllacrididae, Stenopelmatidae, Anostostomatidae (51), G3: Gryllidea (15), G4: Caelifera (161)

unique quartets: 825,930

Topology	original (support in %)	permutation I (support in %)	permutation II (support in %)	permutation III (support in %)
T1	65.4	20.5	14.7	32.9
T2	7.4	19.8	15.4	29.6
T3	22.4	23.6	17.7	32.2
T1T2	1.5	8.8	13.0	1.7
T1T3	2.1	9.8	12.5	1.7
T2T3	0.6	9.1	11.5	1.7
T*	0.5	8.4	15.3	0.1

All six original (non-permuted) dataset favor a sistergroup relationship of Rhaphidophoridae to (Schizodactylidae ((Prophalangopsidae + Tettigoniidae) + (Gryllacrididae (Stenopelmatidae + Anostostomatidae))), T1 (G1, G2 – G3, G4). This is comparable to the ML tree reconstruction of three datasets, $D_{aa,trans,complete}$, $D_{aa,trans,strict}$, $D_{nt,trans+mito,strict}$. The permutation tests further show that this support is not biased by confounding signal in all six datasets, such as i) among-lineage heterogeneity (heterogeneous composition across amino acid sequences / non-stationary substitution processes) that violate globally stationary, reversible and homogeneous (SRH) conditions [126,127] (permutation I) and ii) non-random distribution of missing data (permutation II) or iii) a mixture of both (permutation III). In contrast, the ML tree reconstruction of the other three datasets ($D_{nt,trans,complete}$, $D_{nt,trans,strict}$, $D_{nt,trans+mito,complete}$) and the associated inferred support for an alternative sistergroup relationship: Gryllidea sistergroup to (Schizodactylidae ((Prophalangopsidae + Tettigoniidae) + (Gryllacrididae (Stenopelmatidae + Anostostomatidae))), T3 (G1, G4 - G2, G3), might be biased. Permutation analyses imply that confounding signal is present and overrule genuine phylogenetic signal. Therefore, we consider the placement of Rhaphidophoridae as sistergroup to the clade (Schizodactylidae ((Prophalangopsidae + Tettigoniidae) + (Gryllacrididae (Stenopelmatidae + Anostostomatidae))) as robust and not biased.

b) Position of Gryllotalpidae within Gryllidea

A sistergroup relationship of Gryllotalpidae to Grylloidea (Gryllidae and Phalangopsidae) is supported only by the two amino-acid transcriptome datasets $D_{aa,trans,complete}$, $D_{aa,trans,strict}$ (see Supplementary Figures S3A,B), T1 (G1, G2 – G3, G4). In contrast, the two nucleotide transcriptome datasets $D_{nt,trans,complete}$, $D_{nt,trans,strict}$ as well as the two combined (transcriptomes and mitochondrial) nucleotide dataset $D_{nt,trans+mito,complete}$, $D_{nt,trans+mito,strict}$ (see Supplementary Figures S3C-F) do not support this placement of Gryllotalpidae. Instead Myrmecophilidae is placed as sistergroup to Grylloidea (Gryllidae and Phalangopsidae) and Gryllotalpidae is placed as sistergroup to all remaining Gryllidea, T3 (G1, G4 - G2, G3).

$D_{aa,trans,complete}$ (T1 preferred in ML tree): 1,486,473 aa, 1,536 metapartitions

G1: Gryllotalpidae (1), G2: Grylloidea (2), G3: Myrmecophilidae (1), G4: remaining Ensifera (12)

unique quartets: 24

Topology	original (support in %)	permutation I (support in %)	permutation II (support in %)	permutation III (support in %)
T1	58.3	29.2	16.7	25.0
T2	8.3	58.3	58.3	29.2
T3	33.3	8.3	16.7	29.2
T1T2	0	4.2	8.3	4.2
T1T3	0	0	0	4.2
T2T3	0	0	0	8.3
T*	0	0	0	0

$D_{aa,trans,strict}$ (T1 preferred in ML tree): 436,488 aa, 102 metapartitions

G1: Gryllotalpidae (1), G2: Grylloidea (2), G3: Myrmecophilidae (1), G4: remaining Ensifera (12)

unique quartets: 24

Topology	original (support in %)	permutation I (support in %)	permutation II (support in %)	permutation III (support in %)
T1	54.2	12.5	50.0	33.3
T2	8.3	25.0	16.7	29.2
T3	37.5	41.7	12.5	25.0
T1T2	0	0	12.5	0
T1T3	0	8.3	4.2	12.5
T2T3	0	12.5	4.2	0
T*	0	0	0	0

$D_{nt,trans,complete}$ (T3 preferred in ML tree): 1,459,666 nt, 1,450 metapartitions
G1: Gryllotalpidae (1), G2: Grylloidea (2), G3: Myrmecophilidae (1), G4: remaining Ensifera excluding Rhaphidophoridae (9)

unique quartets: 18

Topology	original (support in %)	permutation I (support in %)	permutation II (support in %)	permutation III (support in %)
T1	38.9	38.9	44.4	38.9
T2	0	22.2	50.0	27.8
T3	55.6	33.3	0	16.7
T1T2	0	0	5.6	0
T1T3	5.6	5.6	0	16.7
T2T3	0	0	0	0
T*	0	0	0	0

$D_{nt,trans,strict}$ (T3 preferred in ML tree): 436,488 nt, 102 metapartitions
G1: Gryllotalpidae (1), G2: Grylloidea (2), G3: Myrmecophilidae (1), G4: remaining Ensifera excluding Rhaphidophoridae (9)

unique quartets: 18

Topology	original (support in %)	permutation I (support in %)	permutation II (support in %)	permutation III (support in %)
T1	61.1	16.7	55.6	50.0
T2	0	5.6	16.7	22.2
T3	33.3	55.6	11.1	16.7
T1T2	0	5.6	0	11.1
T1T3	5.6	11.1	11.1	0
T2T3	0	0	5.6	0
T*	0	5.6	0	0

$D_{nt,trans+mito,complete}$ (T3 preferred in ML tree): 1,480,558 nt, 1,473 metapartitions
G1: Gryllotalpidae (1), G2: Grylloidea (9), G3: Myrmecophilidae (2), G4: remaining Ensifera excluding Rhaphidophoridae (57)

unique quartets: 4,104

Topology	original (support in %)	permutation I (support in %)	permutation II (support in %)	permutation III (support in %)
T1	3.9	21.2	14.7	29.3
T2	63.5	23.3	15.5	29.7
T3	29.6	25.6	23.7	35.8
T1T2	0.9	8.0	12.2	1.8
T1T3	0.3	8.1	11.5	1.5
T2T3	1.7	8.8	11.6	1.8
T*	0	5.0	10.6	0.1

$D_{nt,trans+mito,strict}$ (T3 preferred in ML tree): 457,380 nt, 125 metapartitions
G1: Gryllotalpidae (1), G2: Grylloidea (9), G3: Myrmecophilidae (2), G4: remaining Ensifera excluding Rhaphidophoridae (57)

unique quartets: 4,104

Topology	original (support in %)	permutation I (support in %)	permutation II (support in %)	permutation III (support in %)
T1	7.1	23.8	16.2	26.9
T2	53.2	16.4	18.1	22.5
T3	36.1	25.7	15.6	34.5
T1T2	0.7	8.4	13.5	4.0
T1T3	0.6	10.7	11.3	5.0
T2T3	2.2	8.7	12.5	5.7
T*	0.1	6.3	12.9	1.5

All six original (non-permuted) dataset favor a different position of Gryllotalpidae within Gryllidea. The three datasets $D_{aa,trans,complete}$, $D_{aa,trans,strict}$ and $D_{nt,trans,strict}$ favor a sistergroup relationship of Gryllotalpidae to Grylloidea (Gryllidae and Phalangopsidae), T1 (G1, G2 – G3, G4). This is comparable to the ML tree reconstruction of the two amino-acid transcriptome datasets $D_{aa,trans,complete}$, $D_{aa,trans,strict}$. However, permutation tests further show that only in dataset $D_{aa,trans,complete}$ this support is not biased by confounding signal, such as i) among-lineage heterogeneity (heterogeneous composition across amino acid sequences / non-stationary substitution processes) that violate globally stationary, reversible and homogeneous (SRH) conditions (permutation I) and ii) non-random distribution of missing data (permutation II) or iii) a mixture of both (permutation III). In contrast, in dataset $D_{aa,trans,strict}$ and $D_{nt,trans,strict}$ possible impact from confounding signal for the support of Gryllotalpidae sistergroup to Grylloidea in the original (non-permuted) dataset (54.2% and 61.1%, respectively) might be caused by non-random distribution of missing data, permutation II (50.0% and 55.6% respectively), and randomized distribution of (missing) data in dataset $D_{nt,trans,strict}$, permutation III (50.0%). Dataset $D_{nt,trans,complete}$ favor a sistergroup relationship of Myrmecophilidae to Grylloidea, T3 (G1, G4 - G2, G3), which is also comparable to the ML reconstruction of this dataset. Permutation tests show this support is not biased by confounding signal. The two combined transcriptome mitochondrial datasets $D_{nt,trans+mito,complete}$ and $D_{nt,trans+mito,strict}$ favor a sistergroup relationship of Gryllotalpidae to Myrmecophilidae, T2 (G1, G3 – G2, G4). This relationship is not inferred by any ML tree reconstruction but the permutation tests imply that this support in the original (non-permuted) dataset (63.5% and 53.2%, respectively) is not biased by confounding signal. Please note the small number of evaluated quartets for the transcriptome only datasets (18 and 24 quartets, respectively) and the small taxon sampling for Gryllotalpidae (=1) and Myrmecophilidae (=1 or =2) in all datasets. Therefore, the results should be taken with caution and we consider the position of Gryllotalpidae within Gryllidea as not strongly supported by our datasets. Our taxon sampling is not sufficient to make unambiguous conclusions about this relationship within Gryllidea.

c) Position of Pamphagidae within Caelifera

A sistergroup relationship of Pamphagidae to (Romaleidae + Acrididae) is supported by the two strict transcriptome datasets $D_{aa,trans,strict}$, $D_{nt,trans,strict}$ as well as the two combined (transcriptomes and mitochondrial) nucleotide dataset $D_{nt,trans+mito,complete}$, $D_{nt,trans+mito,strict}$ (see Supplementary Figures S3B,D,E,F), T1 (G1, G2 – G3, G4). In contrast, the two complete transcriptome datasets $D_{aa,trans,complete}$, $D_{nt,trans,complete}$ (see Supplementary Figures S3A,C) do not support this placement of Pamphagidae. Instead Pyrgomorphidae is placed as sistergroup to (Romaleidae + Acrididae), and Pamphagidae as sistergroup to this whole clade (Pyrgomorphidae + (Romaleidae + Acrididae)), T3 (G1, G4 - G2, G3).

$D_{aa,trans,strict}$ (T1 preferred in ML tree): 436,488 aa, 102 metapartitions

G1: Pamphagidae (1), G2: Romaleidae+Acrididae (12), G3: Pyrgomorphidae (15), G4: remaining Caelifera (6)

unique quartets: 1,080

Topology	original (support in %)	permutation I (support in %)	permutation II (support in %)	permutation III (support in %)
T1	56.6	40.7	33.3	31.6
T2	18.8	18.6	24.4	28.8
T3	24.0	24.9	24.6	19.9
T1T2	0.2	4.2	5.6	8.0
T1T3	0.4	6.9	6.0	4.1
T2T3	0.1	4.0	4.7	6.3
T*	0	0.7	1.3	1.4

$D_{nt,trans,strict}$ (T1 preferred in ML tree): 436,488 nt, 102 metapartitions

G1: Pamphagidae (1), G2: Romaleidae+Acrididae (12), G3: Pyrgomorphidae (15), G4: remaining Caelifera (6)

unique quartets: 1,080

Topology	original (support in %)	permutation I (support in %)	permutation II (support in %)	permutation III (support in %)
T1	51.7	36.9	41.4	30.7
T2	23.6	19.0	15.6	21.5
T3	21.7	28.1	21.4	28.1
T1T2	1.7	4.8	7.3	6.7
T1T3	0.8	6.5	8.2	5.9
T2T3	0.6	3.9	4.2	4.5
T*	0	0.8	1.9	2.5

$D_{nt,trans+mito,complete}$ (T1 preferred in ML tree): 1,495,171 nt, 1,534 metapartitions

G1: Pamphagidae (11), G2: Romaleidae+Acrididae (99), G3: Pyrgomorphidae (29), G4: remaining Caelifera (22)

unique quartets: 694,782

Topology	original (support in %)	permutation I (support in %)	permutation II (support in %)	permutation III (support in %)
T1	79.0	23.8	18.1	32.1
T2	8.0	20.6	16.4	31.2
T3	10.1	22.2	15.8	31.5
T1T2	1.2	8.8	12.1	1.7
T1T3	1.0	9.4	11.4	1.7
T2T3	0.4	9.3	13.9	1.7
T*	0.1	6.0	12.3	0.1

$D_{nt,trans+mito,strict}$ (T1 preferred in ML tree): 1,495,171 nt, 1,534 metapartitions
G1: Pamphagidae (11), G2: Romaleidae+Acrididae (99), G3: Pyrgomorphidae (29), G4: remaining Caelifera (22)

unique quartets: 694,782

Topology	original (support in %)	permutation I (support in %)	permutation II (support in %)	permutation III (support in %)
T1	77.7	21.3	17.1	28.8
T2	7.9	19.0	16.8	26.2
T3	11.5	24.5	15.0	26.7
T1T2	1.3	8.2	12.5	5.5
T1T3	1.2	10.1	11.8	5.8
T2T3	0.4	10.2	13.8	5.5
T*	0.1	6.7	12.9	1.4

$D_{aa,trans,complete}$ (T3 preferred in ML tree): 1,506,115 aa, 1,611 metapartitions
G1: Pamphagidae (1), G2: Romaleidae+Acrididae (12), G3: Pyrgomorphidae (15), G4: remaining Caelifera (6)

unique quartets: 1,080

Topology	original (support in %)	permutation I (support in %)	permutation II (support in %)	permutation III (support in %)
T1	69.5	42.4	37.2	24.8
T2	8.8	18.4	18.9	24.1
T3	21.6	34.2	37.0	46.1
T1T2	0.1	1.1	1.4	0.8
T1T3	0	3.0	3.3	1.8
T2T3	0	0.9	1.8	2.3
T*	0	0	0.4	0.1

$D_{nt,trans,complete}$ (T3 preferred in ML tree): 1,474,279 nt, 1,511 metapartitions
G1: Pamphagidae (1), G2: Romaleidae+Acrididae (12), G3: Pyrgomorphidae (15), G4: remaining Caelifera (6)

unique quartets: 1,080

Topology	original (support in %)	permutation I (support in %)	permutation II (support in %)	permutation III (support in %)
T1	57.3	49.8	46.9	23.1
T2	15.5	17.3	16.9	29.8
T3	25.5	27.5	32.1	41.8
T1T2	0.9	1.3	1.1	1.4
T1T3	0.3	3.1	1.9	1.5
T2T3	0.5	0.7	1.9	2.4
T*	0	0.2	0.1	0.1

All six original (non-permuted) dataset favor a sistergroup relationship of Pamphagidae to (Romaleidae + Acrididae), T1 (G1, G2 – G3, G4). This is comparable to the ML tree reconstruction of four datasets, $D_{aa,trans,strict}$, $D_{nt,trans,strict}$, $D_{nt,trans+mito,complete}$, $D_{nt,trans+mito,strict}$. However, permutation tests further show that only in two datasets ($D_{nt,trans+mito,complete}$, $D_{nt,trans+mito,strict}$) this support is not biased by confounding signal, such as i) among-lineage heterogeneity (heterogeneous composition across amino acid sequences / non-stationary substitution processes) that violate globally stationary, reversible and homogeneous (SRH) conditions (permutation I) and ii) non-random distribution of missing data (permutation II) or iii) a mixture of both (permutation III). In contrast, in dataset $D_{aa,trans,strict}$ and $D_{aa,trans,complete}$ the support for a sistergroup relationship of Pamphagidae to (Romaleidae +

Acrididae) might be biased. Permutation I (40.7% and 42.4%, respectively) revealed that among-lineage heterogeneity and non-randomly distribution of missing data might likely explain the support for Pamphagidae sistergroup to (Romaleidae + Acrididae) in the original (non-permuted) dataset (56.6% and 69.5% respectively). In dataset $D_{nt,trans,strict}$ and $D_{nt,trans,complete}$ possible impact from confounding signal for the support of Pamphagidae sistergroup to (Romaleidae + Acrididae) in the original (non-permuted) dataset (51.7% and 57.3%, respectively) might rather be caused by non-random distribution of missing data, permutation II (41.4% and 46.9% respectively). Please note the small taxon sampling for Pamphagidae (=1) in some of the datasets, namely $D_{aa,trans,complete}$, $D_{nt,trans,complete}$, $D_{aa,trans,strict}$, $D_{nt,trans,strict}$. Therefore, the results should be taken with caution and we consider the position of Pamphagidae as sistergroup to (Romaleidae + Acrididae) as not strongly supported by our datasets. Our taxon sampling, especially for $D_{aa,trans,complete}$, $D_{nt,trans,complete}$, $D_{aa,trans,strict}$, $D_{nt,trans,strict,}$ is not sufficient to make unambiguous conclusions about this relationship within Caelifera. However, based on morphological data and previous molecular phylogeny, Pamphagidae is clearly a member of Acridoidea.

Divergence time estimation

We selected 11 fossils (see Section 2.1; Supplementary Figures S4A-B, Supplementary Table 6) to calibrate our divergence-time analysis. All the calibrations, including the root age, were set to soft maximum bound at 412 million years ago (MYA) using uniform priors. We selected the oldest age of Rhynie Chert[128] as the maximum root age because it is a diverse fossil deposit of many well-preserved plants and animals, but lacks winged-insects, and predate all known winged-insect fossils. Each calibration point option was as follows, offset 0.1, scale parameter 1 and left tail probability 0.025.

We used a modified version of the strict transcriptome only dataset ($D_{aa,trans,strict}$) for divergence date inference. This modification, in terms of a reduction of the dataset containing only sites with unambiguous data for at least 80% of the 60 taxa (i.e. "reduced $D_{aa,trans,strict}$"), was necessary to overcome computational limitations when estimating node ages resulting from the large size of the dataset. Previous studies have shown that results of dating analysis are robust to missing data patterns and this dataset reduction (see Evangelista et al.[129]). In addition, to further reduce computational effort, we chose an unpartitioned dating analysis.

We ran divergence time analyses of the unpartitioned dataset using MCMCTree implemented in the software package PAML v4.9[130]. We set the model LG (aaRatefile = lg.dat) + G with 5 rate categories, empirically estimated base frequencies (model = 2) and allowed rates to be inferred from individual sites (RateAncestor = 1). We conducted Hessian matrix calculations according to the above specifications with CODEML as implemented in PAML using empirical +F base frequencies estimated from the respective dataset. Model parameters were specified as follows: chronograms were estimated under the correlated independent rates clock model as done by Evangelista et al.[129]. MCMC chains ran for 1,000,000 generations (sfreq = 50) while discarding a burn-in of 100,000 generations. A total of four independent runs were done at the University of Memphis HPC cluster and using Texas A&M HPC cluster. The effective sample size was checked with the Tracer v1.7.1[131] (ESS > 200). For each dataset, posterior mean time estimates, as well as lower and upper confidence intervals (CI), from all four independent runs were plotted against each other to check for MCMC chain convergence. All four runs converged (see Supplementary Figure 5). From the four replicates of each dataset, we choose posterior means and CI of one randomly selected run, since all four replicates delivered effectively identical results. The inferred dates and 95% confidence intervals (CI) from all dated trees can be found on Dryad (https://doi.org/10.5061/dryad.qjq2bvqc6).

When we used all 11 fossil calibration points, the resulting estimates for those 11 nodes were considerably older than the actual calibration points. We initially suspected whether certain calibration points were pulling all the estimates to the older ages. So, we performed a sensitivity analysis by excluding different calibration points to test if the resulting estimates would change. The result of this sensitivity analysis is Supplementary Table 7. The sensitivity analysis showed that there was little impact of excluding certain calibration points and the resulting estimates were always older than the original fossil calibrations. Therefore, we decided to use the divergence times estimated from including all 11 fossil calibration points to infer temporal patterns of divergence.

Discussion on recovered topology and divergence time estimates

Our analyses confirmed the monophyly of Orthoptera and its two suborders, Ensifera and Caelifera. Moreover, we recovered a comparatively ancient age for crown-Orthoptera, at ~355 million years ago (Mya) [95% credibility interval (CI), 393.8 to 320.0 million years (My)], which is ~63 My earlier than a previous estimate for this group. Our estimate is also ~30 Myr older than that obtained in a recent study focusing on Blattodea, in which Orthoptera represented an outgroup[129]. This estimate is older than the oldest fossil record, an ~307 million-years-old earliest definitive stem-Orthoptera[129,132,133] (see SI S2). However, it shall also be considered that identifying stem-Ensifera and stem-Caelifera from fossils is not straightforward. The former is believed to be characterised by their sword-like ovipositor (hence the name), but the acquisition of this trait likely predated that of Ensifera as currently defined[134,135]. In other words, Caelifera likely diverged from a stem-orthopteran possessing a sword-like ovipositor. As for Caelifera, the earliest putative stem-member is about 250 million-years-old[134,135]. However, the earlier

Permian representatives of Orthoptera, the diversified 'oedischioid complex', might well have included stem-Caelifera that we fail to identify as such. In summary, it is not unlikely that actual Early Permian crown-Orthoptera have already been collected, which would imply an earlier age for the crown-group, consistent with our estimate.
We estimated crown-Ensifera to have appeared during the Late Carboniferous (308 Mya; CI, 348.0 to 267.4 Mya), which is consistent with the known fossil record, with the earliest stem-Ensifera being 272 million-years-old (see SI S2). Our analyses recovered two monophyletic infraorders within this group, Gryllidea and Tettigoniidea, the former consisting of Grylloidea, Gryllotalpidae, and Myrmecophilidae, and the latter consisting of the remaining families. While Gryllidea appears to be an ancient group, crown-Gryllidea is comparatively recent, with an estimated origin of 200 My (CI, 247.5 to 154.1 My). However, the group has numerous candidate stem-relatives during the Triassic, including species belonging to the genera *Gryllavus* and *Protogryllus* in which the tegmina possessed a stridulatory file[136]. One family in this lineage, Myrmecophilidae, is wingless and earless, and evolved in association with ants[137]. Given that ants are thought to have originated 140 Mya[138], it can be reasonably assumed that the group secondarily lost the ability to use acoustic signalling relatively recently, as a consequence of this association.
We estimated that crown-Tettigoniidea originated in the Permian (268 Mya; CI, 308.1 to 227.7 Ma) and diverged into its major extant lineages throughout the Mesozoic. We found Rhaphidophoridae to have diverged first, in the Late Permian. Members of this family are wingless, leaving us to speculate on which fossils might represent their stem-relatives. Schizodactylidae, a relict group endemic to southern Africa and the Middle East, was recovered as sister group to the remaining Tettigoniidea, and originated at the end of the Triassic. There is a significant gap of ~100 My between this estimate and the earliest fossil representative of this group, '*Schizodactylus*' *groeningae* (see SI S2). However, extant Schizodactylidae are atypical in many respects, including a peculiar coiling of their wing apparatus at rest[139], making it difficult to identify their less derived relatives. Fossils of stem-relatives have likely already been collected, cannot yet be identified as such. This would explain the observed temporal gap.
Gryllacrididae, Stenopelmatidae and Anostostomatidae formed a monophyletic group, the superfamily Stenopelmatoidea, consistent with recent studies[81,140-142]. We estimate that this group arose at the onset of the Cretaceous (145.7 Mya; CI, 181.4 to 112.9 Mya), contradicting the identification of putative Triassic stem-Stenopelmatoidea[140]. Because the wing venation of Prophalangopsidae closely resembles extinct fossil ensiferans[134,135], the phylogenetic placement of this family has been debated, but our study recovered it as sister to Tettigoniidae, which is consistent with a morphology-based phylogeny[143]. This finding is contrary to previous molecular studies, which found Prophalangopsidae to be sister to Stenopelmatoidea, but with low support values[81,141]. Prophalangopsidae and Tettigoniidae are the only groups within Tettigoniidea to possess both well-developed stridulatory structures on tegmina and tibial tympana with crista acustica[143-145].
We estimated that Caelifera originated in the Carboniferous (320 Ma; CI, 359.5 to 282 Ma), but most modern grasshopper diversity arose in the Cenozoic. We found Caelifera to consist of two monophyletic infraorders, Tridactylidea and Acrididea. Extant Tridactylidea consists of three small families, including sandgropers (Cylindrachetidae), mud crickets (Ripipterygidae), and pygmy mole crickets (Tridactylidae). There is a prolonged gap more than 200 My between the estimated divergence date and the first known fossil occurrence. However, as for the several cases discussed above, extant representatives are highly derived, impeding identification of early fossil relatives. The more diverse Acrididea originated in the Late Permian (263 Ma; CI, 301.5 to 224.6 Ma) and split into two monophyletic groups, pygmy grasshoppers (Tetrigidae, sole member of Tetrigoidea) and the superfamily group Acridomorpha (grasshopper-like insects). The earliest fossil pygmy grasshoppers are known from the early Cretaceous[146], but it is possible that the lineage originated much earlier. Our analysis placed the origin of Acridomorpha at the Late Triassic (203 Ma; CI, 239.6 to 171.2 Ma), and the earliest diverging lineage within this group includes monkey grasshoppers (Eumastacidae, Thericleidae, Chorotypidae, Episactidae) and jumping sticks (Proscopiidae), and originated in the Jurassic (162.9 Ma; CI, 201.6 to 123.7 Ma). Jumping sticks were previously considered to belong to a separate superfamily[141], but our analyses placed them within the superfamily Eumastacoidea, which suggests that they are highly modified monkey grasshoppers, which has been previously suggested based on male genitalia[147]. Within the remaining Acridomorpha, four relict families with a high level of endemism were found near the base of the phylogeny. Tanaoceridae (3 spp.), endemic to xeric habitats in Lower California and Northern Mexico, diverged the first. Central Mexican endemic Xyronotidae (3 spp.), Southeast Asian endemic Trigonopterygidae (17 spp.), and South African endemic Pneumoridae (17 spp.) formed a monophyletic group in our analysis. These families are morphologically and ecologically divergent from each other and likely originated in the Jurassic. We recovered a sister relationship between gaudy grasshoppers (Pyrgomorphidae, sole member of Pyrgomorphoidea) and common grasshoppers (Acridoidea, for which we included 9 families), which originated in the early Cretaceous (139.2 Ma; CI, 167 to 115.4 Ma). Early diverging lineages within Acridoidea, including Pamphagodidae, Pamphagidae, Pyrgacrididae, Lentulidae, and Lithidiidae, are all endemic to the Old World, while later diverging lineages, including Tristiridae, Romaleidae, and Ommexechidae, are endemic to South America. This biogeographical pattern corresponds well with the split between South America and Africa in the early Cretaceous. Acrididae, which represents the most species-rich clade within Caelifera with more than 6,700 described species, is most closely related to the South American

endemic families, and the earliest diverging lineages within Acrididae are all endemic to South America. This is consistent with a previous finding that Acrididae originated in South America in the Paleogene (60.57 Ma; CI, 75.1 to 48.5 Ma) and quickly spread around the world[12].

Supplementary Methods: Fossil selection

Selected fossil calibrations

Preliminary remarks: We followed a previously established rationale[129] regarding phylogenetic inference, as follows. We selected fossils complying with two cases. First, the fossil can be assigned to a particular group based on a strict synapomorphy (i.e. it belongs to 'Class 1') of that group (i.e. it applies at the 'ultimate level'). The second case implies, at the ultimate level, a derived character state found homoplastic in a broader context ('Class 2'). Selection is validated only if a 'Class 1' character state applying at an earlier node allows other occurrences of the homoplastic character state to be discarded. As an example, displaying a dorsal fin alone is not informative for the systematic placement of a vertebrate. However, in the context of an animal possessing a placenta, a dorsal fin then unambiguously indicates a cetacean. In this example 'dosal fin present' is a Class 2 character state applying at the ultimate level, while 'placenta present' is a Class 1 character state applying at the contextual level. Exceptions to this rationale and other standard criteria[148] compose the extended selection.

Main selection, outgroups

Stem-Isoptera / crown-Blattodea

Valditermes brenanae Jarzembowski, 1981

Comment: Rationale for the selection of this species for that node was provided elsewhere[129] (age, 130.3 My).

Stem-Dictyoptera / crown-(Dictyoptera+(Eukinolabia+Xenonomia))

Qilianiblatta namurensis Zhang, Schneider & Hong, 2013

Comment: Rationale for the selection of this species for that node was provided elsewhere[129] (age, 306.9 My; see 'Locality ages' section).

Stem-Mantophasmatodea / crown-Xenonomia

Juramantophasma sinica Huang, Nel, Zompro & Waller, 2008

Comment: Rationale for the selection of this species for that node was provided elsewhere[129] (age, 158.1 My).

Stem-Embioptera / crown-Eukinolabia

Alexarasnia rossica Gorochov, 2011

Comment: Rationale for the selection of this species for that node was provided elsewhere[129] (age, 254.1 My).

Stem-Dermaptera / crown-(Zoraptera+Dermaptera)

Protelytron permianum Tillyard, 1913

Comment: Rationale for the selection of this species for that node was provided elsewhere[129] (age, 271.8 My).

Main selection, ingroup

Preliminary remarks: Wing venation homologies for Orthoptera have been largely debated and continue to be so. One aspect of the debate relates to conjectures of wing venation homologies of Orthoptera compared to other insects[149]. In short, the 'traditional view' professes that MP has a short oblique portion fusing with a branch of CuA near the wing base[134,135,145,150,151] while, under the 'alternative view', the same pattern is interpreted as CuA (emerging from M+CuA) fusing with a branch of CuP[152,153]. This has implications in the identification of stem-Orthoptera because, under the traditional view, the same pattern is believed to occur in other insect orders. Under the alternative view, the assumed pattern is exclusive of Orthoptera. However, this aspect had no implications on the current selection, because the earliest stem-Orthoptera, whichever view is followed, is of similar age as that of the earliest stem-Dictyoptera (viz. *Qilianiblatta namurensis*), which calibrates a node supposedly more recent. This was discussed elsewhere [see Evangelista et al.[129], case of *Osnogerarus trecwithiensis*].

The second aspect regards wing venation homologies within Orthoptera and, more particularly, in extant Ensifera lineages and their immediate stem-groups. In this debate, one school professes that the stridulatory file is homologous across extant ensiferans[136,145,150,151,154,155], while a variety of proposals assume convergent origins, albeit under different homology conjectures and evolutionary scenarios[143,156,157]. These discrepancies are more problematic. Where necessary and possible, we discuss all options and adopted the conservative one. We generally follow wing venation homologies proposed by Béthoux[136] and Chivers et al.[158], and the associated terminology.

Stem-Ensifera / crown-Orthoptera

Raphogla rubra Béthoux, Nel, Lapeyrie, Gand & Galtier, 2002

Comment: Rationale for the selection of this species for that node was provided elsewhere[129] (age, 271.8 My).

Stem-Gryllidea / crown-Ensifera

Preliminary remarks: Many authors considered Grylloidea and Gryllotalpoidea to be very closely related. Fossil-orientated publications traditionally regarded both groups to have derived from a 'protogryllid' assemblage[134-136,145,151,154] which wing venation compares best with that of extant Gryllidae (e.g. the case of *Gryllavus madygenicus*, see 'Excluded fossil calibrations' section). In other words, the wing venation of Gryllotalpoidae is regarded as derived from a 'grylloid-like groundplan'.) Assuming so, it is not evident to distinguish a stem-Grylloidae from a stem-Gryllotalpoidae, or a stem-Gryllidea. The fossil species selected below falls in this area of uncertainty and is therefore conservatively considered a stem-Gryllidea.
Note that the view that the wing venation of Gryllotalpoidae derived from a 'grylloid-like groundplan' was recently challenged by Desutter-Grandcolas et al.[157], who assumed that the two groups possess more fundamentally different wing venation patterns. However, the lack of interpretation on wing venation except for wing bases makes it difficult to appreciate the characteristics of the various groups, in the more distal area, under the proposal of these authors. Nevertheless, a fossil specimen displaying a wing venation pattern herein considered plesiomorphic with respect to that of the species selected below was illustrated by these authors. The fossil being considered a Grylloidea, it then follows that the fossil species selected below would likely be considered a stem-Gryllidea by these authors.

Protogryllus dobbertinensis (Geinitz, 1880)

Original description: Geinitz, F.E. (1880) Der Jura in Mecklenburg und seine Versteinerungen. *Zeitschrift der deutschen geologischen Gesellschaft*, **32**, 510–535 + pl. 22.
Further descriptive accounts: Ansorge (1996)[159] described several additional specimens of this species.
Locality: Dobbertin (179.7 My).

CR1 (single/multiple OTUs with museum numbers)**:** Yes (see further descriptive account).
CR2 (apomorphy-based or phylogenetic analysis)**:** Yes (see below).
CR3 (agreement of morphology and molecular data)**:** Yes (see below).
CR4 (detailed locality and stratigraphy data provided)**:** Yes (original description and further descriptive account).
CR5 (radioisotopic age or numeric age references given): Yes (see 'Locality ages' section).

Phylogenetic justification & discussion:
The placement of *Protogryllus dobbertinensis* as stem-Gryllidea (hence crown-Ensifera) is based on the following character states:

Ultimate level:

Class 1: (1) in male forewing, occurrence of the column.

Class 2: (2) in male forewing, in the antero-distal area, occurrence of folds forming a fan; (3) in male forewing, reduction of the pre-mirror area

Contextual level:

Class 1: none found / considered.

Class 2: none found / considered.

We voluntarily restricted the list of relevant character states to those that do not require extensive discussion. For example, the relevance of the large mirror area is difficult to appreciate under the obtained topology and, concurrently, under competing conjectures on wing venation homologies.
Under the homology scheme favoured herein[136,158] the occurrence of the column (1) is an apomorphy of Grylloidea and Gryllotalpoidea. We ignore whether this is the case under the proposal by Desutter-Grandcolas et al.[157].
The occurrence of an antero-distal fan (2; also termed 'median fan'[150]) in *Protogryllus dobbertinensis* can be deduced from published photographs[159]. According to Ragge[150] this structure occurs in Grylloidae but is absent in Gryllotalpoidae. However, our observations (OB, 2019) revealed that it is present in the latter. It has also been indicated that a fan occupying the same position, and functioning in the same way, occurs in Schizodactylidae[150]. Given the obtained topology, the 'Grylloidea & Gryllotalpoidea fan' and the 'Schizodactylidae fan' were acquired convergently. The reduction of the pre-mirror area (3) occurred convergently in Tettigoniidae.
Additional relevant character states ('in male forewing, Larunda area free of cross-veins'; 'in male forewing, origin of RP in a distal position or absent') would require alterations of the proposal favoured herein[136]) which do not need to be detailed here, given the compelling evidence on the affinities of this species.

Stem-(Tettigonioidea+Hagloidea) / crown-((Tettigonioidea+Hagloidea)+Stenopelmatoidea)

Preliminary remarks: We failed to identify well-ascertained stem- or crown-members of Stenopelmatoidea (see 'Excluded fossil calibrations' section), therefore we scrutinized stem-(Hagloidea+Tettigonioidea) only (the case of *Permotettigonia galllica* was dealt with elsewhere[129]).

Pseudaboilus wealdensis Gorochov, Jarzembowski & Coram, 2006

Original description: Gorochov, A.V., Jarzembowski, E.A. & Coram, R.A. (2006) Grasshoppers and crickets (Insecta: Orthoptera) from the Lower Cretaceous of southern England. *Cretaceous Research*, **27**, 641-662.
Further descriptive accounts: A photograph of the paratype is reproduced in Gorochov et al., but a photograph of the holotype was published prior to the formal description of the species[160].
Locality: Auclaye Brickworks (ca. 127.5 My).

CR1 (single/multiple OTUs with museum numbers)**:** Yes (see original description).
CR2 (apomorphy-based or phylogenetic analysis)**:** Yes (see below).
CR3 (agreement of morphology and molecular data)**:** Yes (see below).
CR4 (detailed locality and stratigraphy data provided)**:** Yes (original description and further descriptive account).
CR5 (radioisotopic age or numeric age references given): Yes (see 'Locality ages' section).

Phylogenetic justification & discussion:
The placement of *Pseudaboilus wealdensis* as stem-(Tettigonioidea+Hagloidea) (hence crown-((Tettigonioidea+Hagloidea)+Stenopelmatoidea)) is based on the following character states:

Ultimate level:

Class 1: (1) 'string + neck + pre-mirror + h1 stridulatory syndrome'.
Class 2: none found / considered.

Contextual level:

Class 1: none found / considered.
Class 2: none found / considered.

Pseudaboilus wealdensis was assigned by Gorochov et al.[161] to the sub-family Termitidiinae, which best-known species remained *Termitidium ignotum* until the description of new material by these authors. The forewing venation of *Pseudaboilus wealdensis* (Supplementary Figure 6) is straightforward to interpret when compared to that in *Cyphoderris* and Tettigonioidea[158]. The three taxa share the 'string' specialized cross-vein, and the neck and h1 areas free of cross-veins. Moreover, the pre-mirror area is free of cross-veins in both *Pseudaboilus wealdensis* and *Cyphoderris* (this area collapsed in Tettigonioidea). This set of traits is obviously related to the use of the corresponding areas as a single resonator, hence the formulation of character state (1).
A notable difference between *Pseudaboilus wealdensis* on one hand, and *Cyphoderris* and Tettigonioidea on the other, is the occurrence of large, cross-vein free mirror. However, such mirror is lacking in extant Hagloidea other than *Cyphoderris*, such as *Tarragoilus*[162]. Given the obtained topology, the mirror could have equally been gained twice (in *Cyphoderris* and Tettigonioidea) or gained in Tettigonioidea+Hagloidea and secondarily lost in *Tarragoilus*. Also, many extant Hagloidea lack the character state (1)[162]. It either indicates that (i) it was lost in the corresponding taxa, (ii) that the placement of *Tarragoilus* in our analysis is inaccurate, or (iii) that the character state (1) was acquired twice, in *Cyphoderris* and in Tettigonioidea. In any case, *Pseudaboilus wealdensis* is (i) a stem-(Tettigonioidea+Hagloidea), (ii) a stem-(Tettigonioidea+*Cyphoderris*), or (iii) a stem-*Cyphoderris* or a stem-Tettigonioidea. It is then conservative to consider it a stem-(Tettigonioidea+Hagloidea).

Stem-Gryllotalpoidea / crown-(Gryllotalpoidea+Grylloidea)

Cratotetraspinus fossorius (Martins-Neto, 1995)

Original description: Martins-Neto, R.G. (1995) Complementos ao estudo sobre os Ensifera (Insecta, Orthopteroida) da Formação Santana, Cretáceo inferior do Nordeste do Brasil. *Revista brasileira de Entomologia*, **39**, 321–345.
Further descriptive accounts: The holotype of the species is probably lost. Another, well-preserved specimen was illustrated by Heads & Martins-Neto[163].
Locality: Crato (112.6 My).

CR1 (single/multiple OTUs with museum numbers)**:** Holotype probably lost, see further descriptive account.
CR2 (apomorphy-based or phylogenetic analysis)**:** Yes (see below).
CR3 (agreement of morphology and molecular data)**:** Yes (see below).
CR4 (detailed locality and stratigraphy data provided)**:** Yes (original description and further descriptive account).
CR5 (radioisotopic age or numeric age references given): Yes (see 'Locality ages' section).

Phylogenetic justification & discussion:
The placement of *Cratotetraspinus fossorius* as stem-Gryllotalpoidea [hence crown-(Gryllotalpoidea+Grylloidea)] is based on the following character states:

Ultimate level:

Class 1: none found / considered.
Class 2: (1) in foreleg, occurrence of a tibial process bearing at least two dactyls.

Contextual level:

Class 1: none found / considered.

Class 2: none found / considered.

Plesiomorphy: (2) antennae long.

The holotype of the species is probably lost. Illustrations in the original description[164] demonstrate the occurrence of the character state (1). The specimen SMNK PAL 5477, documented in Heads & Martins-Neto[163] and presumably belonging to the same species (but see below), also displays this state. Note that occurrence of long antennae, although a plesiomorphy, allows affinities with crown-Caelifera sharing the character state (1) to be ruled out. Finally, it should be acknowledged that the specific assignment of the specimen SMNK PAL 5477 is not totally evident, given that the holotype was exposed in a lateral position, while the specimen SMNK PAL 5477 is exposed dorsally.

Extended selection, ingroup

Stem-Tridactylidae / crown-(Tridactylidae+Ripipterygidae)

Cratodactylus ferreirai Martins-Neto, 1990

Original description: Martins-Neto, R.G. (1990) Um novo gênero e duas nov expécies de Tridactylidae (Insecta, Orthopteridea na Formação Santana (Cretáceo Inferior do Nordeste do Brasil). *Anais da Academia Brasileira de Ciências*, **62**, 51–59.

Further descriptive accounts: Drawings in the original description were reproduced in various publications, including Heads & Martins-Neto[163].

Locality: Crato (112.6 My).

CR1 (single/multiple OTUs with museum numbers)**:** Yes, but material probably lost.

CR2 (apomorphy-based or phylogenetic analysis)**:** Yes (see below).

CR3 (agreement of morphology and molecular data)**:** Yes (see below).

CR4 (detailed locality and stratigraphy data provided)**:** Yes (original description and further descriptive account).

CR5 (radioisotopic age or numeric age references given): Yes (see 'Locality ages' section).

Phylogenetic justification & discussion:

The placement of *Cratodactylus ferreirai* as stem-Tridactylidae (hence crown-(Tridactylidae+Ripipterygidae)) is based on the following character states:

Ultimate level:

Class 1: none found / considered.

Class 2: (1) foreleg short, with tibia significantly expanded and bearing dactylar processes.

Contextual level:

Class 1: (2) antennae short; (3) hind leg tarsus one-segmented; (4) hind leg metatarsus long with teeth-like apex.

Class 2: none found / considered.

The species is known from specimens in both lateral and dorsal orientation[165]. Within Orthoptera, the character state (2) (visible in specimens preserved in dorsal orientation) indicates relationships with Caelifera (the state 'long antennae' is a plesiomorphy within Orthoptera, it occurs in all stem-Orthoptera in which the character is documented[134,135]). Within Caelifera, the character state (3) (visible in the holotype, preserved in lateral orientation) clearly indicates relationships with the Tridactyloidea[166]. The character state (4), herein regarded as a derived condition, indicates relationships with Tridactylidae+Ripipterygidae. Note, however, that the metatarsus is fully reduced (and, conversely, apical spurs elongated) within Tridactylinae[167], but this is regarded as an apomorphic condition [and, therefore, does not preclude *Cratodactylus ferreirai* from being a stem-(Tridactylidae+Ripipterygidae)]. Its occurrence in the holotype of *Cratodactylus ferreirai* can be guessed based on published data. Ultimately, in this context, possessing the character state (1), documented in *Cratodactylus ferreirai* thanks to specimens preserved in dorsal orientation, indicates relationships with Tridactylidae (Ripiterygidae also possess 'fossorial forelegs', but tibiae are not significantly expanded in representatives of this family).

The current location of the collection containing the material of *Cratodactylus ferreirai* is unknown. Therefore, this case fails to fulfil **CR1** and was then relegated to the extended selection.

Stem-Gomphocerinae+Acridinae+Oedipodinae / crown-((Gomphocerinae+Acridinae+Oedipodinae)+Catantopinae))

Tyrbula russelli Scudder, 1885 in Schimper et al., 1885

Original description: Schimper, W.P., Schenk, A. & Scudder, S.H. (1885) Handbuch der Palaeontologie I. Abtheilung. Palaeozoologie. II. Band. Mollusca und Arthropoda. *Handbuch der Palaeontologie* (ed. by A. Zittel), pp. 1–893. Verlag von R. Oldenbourg, Munich & Leipzig, Germany.

Further descriptive accounts: The species was redescribed by Scudder (1890)[168]. Photographs of the holotype can be accessed from the online database of the Museum of Comparative Zoology.

Locality: Florissant (33.9 My).

CR1 (single/multiple OTUs with museum numbers)**:** Yes (see further descriptive accounts).
CR2 (apomorphy-based or phylogenetic analysis)**:** No (but see below).
CR3 (agreement of morphology and molecular data)**:** Yes (see below).
CR4 (detailed locality and stratigraphy data provided)**:** Yes (original description and further descriptive account).
CR5 (radioisotopic age or numeric age references given): Yes (see 'Locality ages' section).

Phylogenetic justification & discussion:
The placement of *Tyrbula russelli* as Gomphocerinae+Acridinae+Oedipodinae (hence crown-((Gomphocerinae+Acridinae+Oedipodinae)+Catantopinae)) is based on the following character states:

Ultimate level:

Class 1: none found / considered.

Class 2: (1) clubbed antennae; (2) slanted face.

Contextual level:

Class 1: none found / considered.

Class 2: none found / considered.

Clubbed antennae are relatively rare among Acrididae. Although they occur in several lineages, they are most prevalent in Gomphocerinae. The slanted face observed in *Tyrbula russelli* is also indicative of its relationship to Gomphocerinae, whose common name is slanted-faced grasshopper. Within Acrididae, prominently slanted face is found in Gomphocerinae and Acridinae. Gomphocerinae, Acridinae, and Oedipodinae form a monophyletic group, and they are the only acridid group without prosternal process. While the general habitus of the fossil specimen is highly indicative of a typical gomphocerine, the prosternal process is not well-preserved enough to clearly see this apomorphy. This case fails to fulfil **CR2** and was then relegated to the extended selection.

Excluded fossil calibrations

Eolocustopsis primitiva Riek, 1976

Preliminary remarks: The species was used as temporal calibration point by Song et al.[141] as stem-Caelifera (hence crown-Orthoptera).

Original description: Riek, E.F. (1976) New Upper Permian insects from Natal, South Africa. *Annals of the Natal Museum*, **22**, 755–789.
Further descriptive accounts: None.
Locality: Beaufort group (251.9 My[169]).

Discussion: Despite its incompleteness, the known specimen can be assigned with some degree of confidence to the 'Locustopseidae' (a set of stem-Caelifera otherwise known in the Triassic and Jurassic) owing to the sharp bending of CuA+CuPaα. It can also be assessed that it lacks the free oblique section of CuA (between its divergence from M+CuA and its fusion with CuPaα) known in Caelifera.

However, the species is younger than *Raphogla rubra*[169], herein selected as the earliest stem-Ensifera (see 'Selected fossil calibrations' section). Both allowing the calibration of the same node (namely, the Ensifera-Caelifera split), *Eolocustopsis primitiva* was not further considered.

Gryllacrimima perfecta Sharov, 1968

Preliminary remarks: The species was used as temporal calibration point by Vandergast et al.[142] as stem-Stenopelmatoidea [hence crown-(Stenopelmatoidea+(Hagloidea+Tettigonioidea))].

Original description: Sharov, A.G. (1968) Filogeniya orthopteroidnykh nasekomykh. *Trudy Paleontologicheskogo instituta, Akademiya Nauk SSSR*, **118**, 1–216.
Further descriptive accounts: Sharov's[134] account has an English translation[135] including reproductions of the 1968 figures. Gorochov[170] described a number of closely related species from the same locality, further considered synonyms of *Gryllacrimima perfecta* by Béthoux[140], who provided photographs and new drawings.
Locality: Madygen (226.4 My).

Discussion: Systematic affinities of this species, and of closely related ones from sub-contemporaneous localities, have been debated[134,135,171]. Béthoux[140] considered the species a close relative of Stenopelmatoidea (therein assigned to the taxon '*Agryllacris*', encompassing crown-Stenopelmatoidea and some stem-relatives). The fact the Gryllacrididae, Stenopelmatidae and Anostomatidae are recovered as a monophylum (viz. Stenopelmatoidea) in our analysis provides support to the '*Agryllacris*' hypothesis, resting on the character state 'in forewing, CuA+CuPaα with two branches only'.

However, this character state is not unique to the taxon: it can be found within Caelifera, some members of the fossil families Elcanidae and Bintoniellidae, etc. It is, however, believed to have been acquired convergently in these groups, but this assertion would require a formal cladistic analysis including numerous fossils. Another point is that it cannot be ruled out that state was present in stem-Schizodactylidae and stem-Rhaphidophoridae

(the latter being wingless). As a consequence, the placement of *Gryllacrimima perfecta* as stem-Stenopelmatoidea cannot be considered well-ascertained.

Gryllavus madygenicus Sharov, 1968

Original description: Sharov, A.G. (1968) Filogeniya orthopteroidnykh nasekomykh. *Trudy Paleontologicheskogo instituta, Akademiya Nauk SSSR*, **118**, 1–216.
Further descriptive accounts: Sharov's[134] account has an English translation[135] including reproductions of the 1968 figures. Gorochov[172] described a number of species, recovered from the same locality as *Gryllavus madygenicus*, which likely are junior synonyms of this species. These include *Paragryllavus curvatus* and *Zagryllavus elongatus*. The holotypes of both species display the character state (1) mentioned below. Photographs of the holotype of *Paragryllavus curvatus* were reproduced in Béthoux[136], including a photograph of the stridulatory file.
Locality: Madygen (226.4 My[169]).

Discussion: In the original description[134] the species was regarded as a Gryllidae, a taxonomic assignment to be understood nowadays as stem-Gryllidea [we quote, 1971 version, p. 79: "The continuity of the venation of Gryllidae and Gryllotalpidae was recognized by all authors; there is no doubt that the mole crickets developed from the Gryllidae"]. However, the character state listed as diagnostic of the 'Gryllidae' [viz. (1) in forewing, occurrence of a longitudinal fold; (2) in forewing, reduced number of RP branches; and (3) in forewing, CuA+CuPaα reaching wing apex) also occur in the Stenopelmatoidea[140]. [134]) also relied on the organisation of RP, M, and CuA+CuPaα in the antero-distal part of the forewing (see his fig. 29). Indeed, in *Gryllavus madygenicus*, and also in several other species recovered from the same locality[172], in the area between R/RP and MA, in vicinity of the origin of RP, the sigmoid crossveins fuse and form an oblique pseudo-vein. The name 'Larunda area' was given to the area basal to this pseudo-vein[158], and the occurrence of such area was suggested in *Cyphoderris* spp.[158]. Relying on the obtained topology, the state is then homoplastic (putatively lost in Tettigoniidae and Stenopelmatoidea). Therefore, it cannot be excluded that *Gryllavus madygenicus* is a stem-Ensifera (instead of a stem-Gryllidea). If so, given that *Raphogla rubra* applies to the same node and is more ancient, *Gryllavus madygenicus* was not further considered.

Protogryllus sp.

Discussion: According to Song et al.[141], the authors used a Triassic undescribed species of *Protogryllus* as stem-Grylloidea. They referred to Heads & Leuzinger[173], in which the position of the corresponding family (viz. Protogryllidae) is discussed, but no species of *Protogryllus* described. A list of species assigned to this genus was provided elsewhere[174], in which the only enlisted Triassic species is *Protogryllus stormbergensis* (Haughton, 1924)[175]. The available data consist of a drawing in the original description. This drawing suggests that the preserved wing is incomplete and was possibly, at least in part, disrupted and/or creased. In summary, the corresponding material would require a proper revision before being considered for temporal calibration. We failed to obtain new photographs of the material.

'Schizodactylus' groeningae Martins-Neto, 2007

Original description: Heads, S. & Leuzinger, L. (2011) On the placement of the Cretaceous orthopteran *Brauckmannia groeningae* from Brazil, with notes on the relationships of Schizodactylidae (Orthoptera, Ensifera). *Zookeys*, **77**, 17–30.
Further descriptive accounts: Heads & Leuzinger[173] described a specimen additional to the holotype (which current location is unknown).
Locality: Crato (112.6 My[169]).

Discussion: The holotype of the species is probably lost. The original description is deficient in several respects[173], but that of an additional specimen, likely conspecific, by Heads & Leuzinger[173] demonstrates the occurrence of Schizodactylidae at Crato, owing to the occurrence of (1) coiled wings and (2) hind leg basitarsus with enlarged lateral plates. Extant representatives of this family are the genera *Schizodactylus* and *Comicus*, which are winged and wingless, respectively. The placement of the fossil species in the former, suggested by Heads & Leuzinger[173], is not substantiated, the possession of wings being a plesiomorphy. Therefore, it cannot be excluded that *'Schizodactylus' groeningae* is a stem-Schizodactylidae. Assuming so, and considering the obtained topology, the fossil would apply as crown-(Schizodactylidae+(Stenopelmatoidea+Tettignioidea)). However, *Pseudaboilus wealdensis*, which calibrates the Stenopelmatoidea/Tettignioidea split, is older than *'Schizodactylus' groeningae* (see 'Selected fossil calibrations' section). It then follows that the latter is not useful for temporal calibration.

Termitium ignotum Westwood, 1854

Original description: Westwood, J.O. (1854) Contribution to fossil entomology. *Quarterly Journal of the Geological Association of London*, **10**, 378–396.
Further descriptive accounts: Zeuner[145] and Gorochov et al.[161] redescribed the original material.
Locality: Durlston Bay (ca. 140 My).

Discussion: The species is known based on a single left forewing assigned by Gorochov et al.[161] to the same sub-family as *Pseudaboilus wealdensis* (namely, the Termitidiinae), a species we consider a stem-(Tettigonioidea+Hagloidea) (see 'Selected fossil calibrations' section). Given that *Termitidium ignotum* is slightly more ancient than *Pseudaboilus wealdensis*, we scrutinized this case.

According to recent photographs obtained from the NHM staff, the preservation of the material of *Termitidium ignotum* is average. As a consequence it is not evident whether the apparent absence of cross-veins in the neck and harp areas indicates (1) an original lack or (2) poor preservation. Given that the lack of cross-veins in particular areas related to sound-production allow the identification of a stem-(Tettigonioidea+Hagloidea) (see 'Selected fossil calibrations' section), we refrained from using this fossil as calibration point. Note that the corresponding sub-family (viz. Termitidiinae) is not supported by any obvious autapomorphy which would have demonstrated close affinities of *Termitidium ignotum* and *Pseudaboilus wealdensis*.

Locality ages

This section complements that in Evangelista et al.[129].

Xiaheyan (306.9 Ma)

Geographic location: Xiaheyan, Ningxia, China.
Geological settings: Yanghugou Formation.
Stratigraphic data: latest Bashkirian to middle Moscovian.
Age justification: Multiple proxies were used to reassess the age of insect-bearing layers at that locality[176].The Moscovian ended 306.9 Mya[177].

Dobbertin (179.7 My)

Geographic location: Mecklenburg-Vorpommern, Germany.
Geological settings: Green Series.
Stratigraphic data: Lower Toarcian, Lower Jurassic.
Age justification: See Wolfe et al.[169] and references therein.

Auclaye Brickworks (ca. 127.5 My)

Geographic location: Surrey, England.
Geological settings: Upper Weald Clay, Weald Clay Group.
Stratigraphic data: Barremian, Lower Cretaceous.
Age justification: The locality is located at the very base of the Upper Weald Clay[178,179], itself correlated with the Barremian, which ranges between ca. 125.0 and 129.4 MYA. Although the dynamics of sediment accumulation in the Upper Weald Clay cannot be ascertained, 127.5 My can yet be reasonably assumed as minimum age for the Auclaye Brickworks outcrop.

Crato (112.6 My)

Geographic location: Pernambuco, Piauí and **Ceará** states, Brazil.
Geological settings: Crato Formation.
Stratigraphic data: Aptian, Lower Cretaceous.
Age justification: See Wolfe et al.[169] and references therein.

Florissant (33.9 My)

Geographic location: Colorado, USA.
Geological settings: Florissant Formation.
Stratigraphic data: Upper Eocene, Paleogene.
Age justification: Multiple proxies consistently indicate that the fossiliferous layers of the Florissant Formation are of Late Eocene in age[180]. The Eocene ended 33.9 MYA[177].

Supplementary Methods: Character evolution

Ancestral character state reconstruction

To trace the evolution of hearing and sound producing organs along the phylogeny, we first conducted a thorough literature data and physical examination of the specimens to describe the presence/absence of these organs. For hearing organs, we coded whether tympanum was absent, present on thorax (Mantidae), on fore tibiae (Ensifera), or on abdomen (Caelifera). We also included atympanate hearing found in Pneumoridae as one of the states. For sound producing organs, we used a specific naming convention in which the first named structure has the stridulatory file and the second named structure has the scraper. For example, abdominal-femoral stridulation would have the stridulatory files on the abdomen and the scraper on the inner side of hind femora. The possible combinations used were: absent, tegmino-pronotal, tegmino-femoral, tegmino-alary, tegmino-tegminal, abdomino-alary, abdomino-femoral, Krauss's organ-femoral, and femoro-tegminal stridulation. Additionally, we included another type of sound producing, only found in Acrididae, known as crepitation, which produces sound by snapping wings when grasshoppers fold and unfold. The complete list of characters used for this analysis is presented in Supplementary Table 8.

We performed ancestral character state reconstruction of hearing and sound producing organs in a maximum likelihood framework using the topology resulting from the $D_{nt,trans+mito,strict}$. We fitted a continuous-time Markov chain (Mk) single-rate (ER) model to our data to infer character evolution using the R package phytools[181]. The resulting graphs were exported as EPS files and modified in Adobe Illustrator CS2019. All of the raw analyses as well as the transition matrices resulting from each analysis is presented below.

Ancestral character state reconstruction on hearing organs

```
Log-likelihood: -114.9824

Parameter estimates:
 rate index estimate std-err
          1   0.0048   8e-04

Scaled likelihoods at the root:
     abdomen       absent  chordotonal   fore tibia       thorax
0.0009538649 0.9956795892 0.0009339935 0.0014984514 0.0009341011

Transition matrix

Q =
                 abdomen       absent  chordotonal   fore tibia
thorax
abdomen     -0.019331714  0.004832929  0.004832929  0.004832929
0.004832929
absent       0.004832929 -0.019331714  0.004832929  0.004832929
0.004832929
chordotonal  0.004832929  0.004832929 -0.019331714  0.004832929
0.004832929
fore tibia   0.004832929  0.004832929  0.004832929 -0.019331714
0.004832929
thorax       0.004832929  0.004832929  0.004832929  0.004832929 -
0.019331714
```

Ancestral character state reconstruction on sound-producing organs

```
Log-likelihood: -141.9744

Parameter estimates:
 rate index estimate std-err
          1   0.0019   3e-04

Scaled likelihoods at the root:
        abdomino-alary stridulation       abdomino-femoral stridulation
                       0.0002851212                        0.0005723422
```

```
                          absent                                 crepitation
                    0.9930378939                                0.0002850788
       femoro-tegminal stridulation Krauss's organ-femoral stridulation
                    0.0002850788                                0.0002850996
   mandibulo-maxillary stridulation          tegmino-alary stridulation
                    0.0004221453                                0.0005262316
       tegmino-femoral stridulation       tegmino-pronotal stridulation
                    0.0002850788                                0.0002851072
      tegmino-tegminal stridulation         tegmino-tibial stridulation
                    0.0034457438                                0.0002850788
```

Transition matrix

Q =

	abdomino-alary	abdomino-femoral	absent	crepitation	femoro-tegminal	Krauss's organ-femoral	mandibulo-maxillary	tegmino-alary	tegmino-femoral	tegmino-pronotal	tegmino-tegminal	tegmino-tibial
abdomino-alary	-0.021170507	0.001924592	0.001924592	0.001924592	0.001924592	0.001924592	0.001924592	0.001924592	0.001924592	0.001924592	0.001924592	0.001924592
abdomino-femoral	0.001924592	-0.021170507	0.001924592	0.001924592	0.001924592	0.001924592	0.001924592	0.001924592	0.001924592	0.001924592	0.001924592	0.001924592
absent	0.001924592	0.001924592	-0.021170507	0.001924592	0.001924592	0.001924592	0.001924592	0.001924592	0.001924592	0.001924592	0.001924592	0.001924592
crepitation	0.001924592	0.001924592	0.001924592	-0.021170507	0.001924592	0.001924592	0.001924592	0.001924592	0.001924592	0.001924592	0.001924592	0.001924592
femoro-tegminal	0.001924592	0.001924592	0.001924592	0.001924592	-0.021170507	0.001924592	0.001924592	0.001924592	0.001924592	0.001924592	0.001924592	0.001924592
Krauss's organ-femoral	0.001924592	0.001924592	0.001924592	0.001924592	0.001924592	0.001924592	0.001924592	0.001924592	0.001924592	0.001924592	0.001924592	0.001924592
mandibulo-maxillary	0.001924592	0.001924592	0.001924592	0.001924592	0.001924592	0.001924592	-0.021170507	0.001924592	0.001924592	0.001924592	0.001924592	0.001924592
tegmino-alary	0.001924592	0.001924592	0.001924592	0.001924592	0.001924592	0.001924592	0.001924592	-0.021170507	0.001924592	0.001924592	0.001924592	0.001924592
tegmino-femoral	0.001924592	0.001924592	0.001924592	0.001924592	0.001924592	0.001924592	0.001924592	0.001924592	-0.021170507	0.001924592	0.001924592	0.001924592
tegmino-pronotal	0.001924592	0.001924592	0.001924592	0.001924592	0.001924592	0.001924592	0.001924592	0.001924592	0.001924592	-0.021170507	0.001924592	0.001924592
tegmino-tegminal	0.001924592	0.001924592	0.001924592	0.001924592	0.001924592	0.001924592	0.001924592	0.001924592	0.001924592	0.001924592	-0.021170507	0.001924592
tegmino-tibial	0.001924592	0.001924592	0.001924592	0.001924592	0.001924592	-0.021170507	0.001924592	0.001924592	0.001924592	0.001924592	0.001924592	-0.021170507

Our analyses showed that there was a high probability that the common ancestor of Ensifera and Caelifera did not hear, but tympanal hearing evolved independently in the two suborders in different body parts (Figure 3). Within Ensifera, tibial tympana likely evolved three times, once in the common ancestor of the infraorder Gryllidea, once in the common ancestor of Anostostomatidae, and once in the common ancestor of the superfamilies Hagloidea and Tettigonioidea. Based on the current taxon sampling, the secondary loss of hearing occurred in the common ancestor of Myrmecophilidae and some members of Anostostomatidae. Within Caelifera, the ability to hear through abdominal tympana likely evolved three times, once in the common ancestor of Pyrgomorphidae, once in the common ancestor of Pamphagidae, and once in the common ancestor of Romaleidae, Ommexechidae, and Acrididae. Prior to the evolution of abdominal tympana, atympanate hearing independently evolved in Pneumoridae. The precursor sensory organs for both tympanate and atympante caeliferans are the pleural chordotonal organs in the abdomen[182], which are likely to be a phylogenetically conserved trait within Caelifera, although detailed neuroanatomical studies on the lower caeliferans are currently lacking. The secondary loss of abdominal tympana occurred multiple times, especially in those species that lost wings. While our analyses suggested that tympanal hearing evolved multiple times both in Ensifera and Caelifera, our ancestral character state reconstruction did recover a small probability that tibial tympana evolved in the common ancestor of Ensifera and abdominal tympana evolved in the common ancestor of Pyrgomorphidae and Acridoidea (Figure 3). Therefore, we could not completely rule out the possibility that the presence of tympana was the ground plan for each group. The ability to produce sound using stridulatory apparatus evolved multiple times throughout the diversification of Orthoptera. Our analyses suggested that the common ancestor of Orthoptera did not have the ability to produce sound by stridulation. Tegmino-tegminal stridulation likely evolved in the common ancestor of Ensifera, which was secondarily lost in Myrmecophilidae and Rhaphidophoridae and became silent. The common ancestor of the superfamilies Schizodactyloidea and Stenopelmatoidea also lost tegmino-tegminal stridulation, but instead evolved abdomino-femoral stridulation, which was lost in Stenopelmatidae. Although tegmino-tegminal stridulation is a shared trait for all singing ensiferans, the specific mechanics of stridulation differed among the lineages. Within Caelifera, the paired structures that are highly indicative of stridulation evolved multiple times in various lineages, but sound production from these structures has been documented only from a small number of groups that use acoustic signalling for mating. The ability to produce sound thus needs to be confirmed for these understudied caeliferan lineages. With this caveat, our analysis showed that the superfamily Tridactyloidea was the earliest lineage to evolve stridulating mechanisms in Caelifera. Cylindrachetidae uses mandibulo-maxillary stridulation, which is found in both sexes as well as in all nymphal instars[183]. Tridactylidae has a tegmino-alary stridulatory mechanism, but their function is completely unknown. Ripipterygidae does not have any known stridulatory mechanism. Our analysis also showed that Tanaoceridae and the common ancestor of Xyronotidae, Pneumoridae, and Trigonopterygidae were the first caeliferan lineages to evolve acoustic signalling in the context of sexual communication. These families are ancient relic groups, represented by only a small

number of extant species with restricted distributions, and they all use abdomino-femoral stridulation, which is only found in males[184]. This mechanism was lost in Trigonopterygidae. Sound production in Tanaoceridae and Xyronotidae has never been documented, but it is likely that it resembles that in Pneumoridae. Interestingly, these insects are also atympanate and it is possible to hypothesise that hearing in Tanaoceridae and Xyronotidae would be similar to that in Pneumoridae. Pamphagidae is the first tympanate caeliferan lineage to evolve sound production by means of Krauss's organ-femoral stridulation. The Krauss's organ is a specialised plate located on the lower anterior corners of the second abdominal tergite, which is unique to the family and present in both males and females[185]. The sound produced by this mechanism is species-specific and likely used for sexual communication. Pamphagids are unique among caeliferans in that they are known to use a number of different mechanisms for producing sound in addition to the Krauss's organ-femoral stridulation, involving forewings, hind wings, middle tibiae, and thorax[186]. However, due to our limited taxon sampling, we could not trace of the evolution of these other mechanisms. Romaleidae has tegmino-alary stridulation, which is used as defensive signalling when advertising aposematic coloration, but it is not clear how widespread this mechanism is within the family. Ommexechidae has a tegmino-femoral stridulatory mechanism, but its sound production has not been characterized. Within Acrididae, which is the most diverse caeliferan family, several types of sound producing mechanisms evolved, such as tegmino-tibial stridulation found in some members of the subfamilies Oxyinae, Spathosterninae, and Tropidopolinae, mandibulo-mandibular stridulation found in some members of the subfamily Eyprepocnemidinae. The most prominent and well-known sound producing mechanism in Acrididae involves thickened veins on forewings and thickened cuticles on inner side of hind femora, in which male grasshoppers move hind femora up and down to rub against the forewings to produce species-specific songs. Our analysis found that the interaction between these two structures likely evolved in the common ancestor of the subfamilies Acridinae, Gomphocerinae, and Oedipodinae. In Gomphocerinae, this mechanism is manifested as femoro-tegminal stridulation in which a row of stridulatory pegs in the inner side of hind femora rubs against the thick veins in the forewings. In Oedipodinae, the same mechanics is used, but it is manifested as tegmino-femoral stridulation in which a row of stridulatory files on the intercalary veins in the forewing rubs against the scrapers in the hind femora. In Oedipodinae and in some members of Acridinae, crepitation mechanism also evolved which can be used in the context of sexual communication.

Pagel's evolutionary correlation analysis

We performed Pagel's (1994) binary character correlation test using the R package phytools[181] based on the dataset used in the ancestral character state reconstruction to test whether hearing and sound production coevolved within Orthoptera. We pruned the phylogenetic tree to create Orthoptera-only, Ensifera-only, and Caelifera-only datasets to compare and contrast lineage-specific patterns. We recoded different types of tympanal and stridulatory mechanisms as simple presence-absence binary characters for both hearing and sound production to understand the general coevolutionary dynamics of these two traits. The input data for the analysis is found in Supplementary Table 9. For each dataset, we fitted four models of coevolution between hearing and sound production and compared the results using Akaike Information Criterion (AIC): (i) hearing and sound production evolve independently; (ii) the evolution of hearing depends on the evolution of sound production; (iii) the evolution of sound production depends on the evolution of hearing; and (iv) hearing and sound production evolve interdependently. All of the raw analyses as well as the rate matrices resulting from each analysis is presented below. The files used for this analysis can be found in Supplementary Archive 3.

Pagel's binary character correlation test

Orthoptera Only

```
Independent model rate matrix:
                absent|absent absent|present present|absent present|present
absent|absent      -7.4535389       4.020000     3.4335391        0.000000
absent|present      0.8483676      -4.281907     0.0000000        3.433539
present|absent      2.8531078       0.000000    -6.8731077        4.020000
present|present     0.0000000       2.853108     0.8483676       -3.701475

Dependent (x & y) model rate matrix:
                absent|absent absent|present present|absent present|present
absent|absent       -3.928338        2.78899      1.139348        0.000000
absent|present       0.000000       -6.00267      0.000000        6.002670
```

present|absent	11.242519	0.00000	-16.773280	5.530761
present|present	0.000000	0.00000	0.930195	-0.930195

Dependent (x only) model rate matrix:

	absent|absent	absent|present	present|absent	present|present
absent|absent	-4.8649282	3.962071	0.9028576	0.0000000
absent|present	0.8701667	-7.645818	0.0000000	6.7756513
present|absent	11.1690268	0.000000	-15.1310975	3.9620706
present|present	0.0000000	0.000000	0.8701667	-0.8701667

Dependent (y only) model rate matrix:

	absent|absent	absent|present	present|absent	present|present
absent|absent	-3.357217	0.000000	3.357217	0.000000
absent|present	8.592614	-11.949831	0.000000	3.357217
present|absent	2.780511	0.000000	-8.086986	5.306474
present|present	0.000000	2.780511	0.000000	-2.780511

Model fit:

	log-likelihood	AIC
independent	-149.7756	307.5513
dependent x&y	-133.9778	283.9555
**dependent x**	**-134.7691**	**281.5383**
dependent y	-140.7378	293.4756

Weighted AIC

independent	**dependent x**	dependent y	dependent x&y
0.0000	**0.7685**	0.0020	0.2295

Ensifera Only

Independent model rate matrix:

	absent|absent	absent|present	present|absent	present|present
absent|absent	-10.15337	5.286277	4.867097	0.000000
absent|present	0.00000	-4.867097	0.000000	4.867097
present|absent	0.00000	0.000000	-5.286277	5.286277
present|present	0.00000	0.000000	0.000000	0.000000

Dependent (x & y) model rate matrix:

	absent|absent	absent|present	present|absent	present|present
absent|absent	0.000000	0.0000	0.00000	0.000000
absent|present	2.936842	-10.9532	0.00000	8.016360
present|absent	7.232539	0.0000	-14.53057	7.298035
present|present	0.000000	0.0000	0.00000	0.000000

Dependent (x only) model rate matrix:

	absent|absent	absent|present	present|absent	present|present
absent|absent	-5.098704	5.098704	0.000	0.000000
absent|present	0.000000	-11.214776	0.000	11.214776
present|absent	1282.373815	0.000000	-1287.473	5.098704
present|present	0.000000	0.000000	0.000	0.000000

Dependent (y only) model rate matrix:

	absent|absent	absent|present	present|absent	present|present
absent|absent	-6.711044	1.843806	4.867237e+00	0.000000e+00
absent|present	0.000000	-4.867237	0.000000e+00	4.867237e+00
present|absent	0.000000	0.000000	-1.537948e+07	1.537948e+07
present|present	0.000000	0.000000	0.000000e+00	0.000000e+00

Model fit:

log-likelihood AIC

independent	-26.00927	60.01855
dependent x&y	-22.16580	60.33161
dependent x	-22.07688	56.15375
**dependent y**	**-22.04348**	**56.08696**

Weighted AIC

independent	dependent x	**dependent y**	dependent x&y
0.0629	0.4343	**0.4490**	0.0538

Caelifera Only

Independent model rate matrix:

	absent|absent	absent|present	present|absent	present|present
absent|absent	-5.212128	4.025318	1.186810	0.000000
absent|present	6.504865	-7.691675	0.000000	1.186810
present|absent	8.129888	0.000000	-12.155206	4.025318
present|present	0.000000	8.129888	6.504865	-14.634754

Dependent (x & y) model rate matrix:

	absent|absent	absent|present	present|absent	present|present
absent|absent	-3.506272	1.855495	1.650777	0.000000
absent|present	5.225679	-5.225679	0.000000	0.000000
present|absent	11.037315	0.000000	-16.707106	5.669791
present|present	0.000000	0.000000	4.612623	-4.612623

Dependent (x only) model rate matrix:

	absent|absent	absent|present	present|absent	present|present
absent|absent	-3.579341	3.579341	0.000000	0.000000
absent|present	8.980685	-13.047435	0.000000	4.066750
present|absent	10.531643	0.000000	-14.110984	3.579341
present|present	0.000000	0.000000	8.980685	-8.980685

Dependent (y only) model rate matrix:

	absent|absent	absent|present	present|absent	present|present
absent|absent	-1.852239	0.8326746	1.019565	0.000000
absent|present	11.504510	-12.5240746	0.000000	1.019565
present|absent	8.422244	0.0000000	-14.474417	6.052173
present|present	0.000000	8.4222435	0.000000	-8.422244

Model fit:

	log-likelihood	AIC
independent	-111.6349	231.2698
dependent x&y	-106.8660	229.7321
**dependent x**	**-107.7410**	**227.4820**
dependent y	-107.9419	227.8838

Weighted AIC

independent	**dependent x**	dependent y	dependent x&y
0.0656	**0.4361**	0.3567	0.1416

Diversification analysis

For the diversification analysis, we used the program BAMM and the R package BAMMtools[187]. Because BAMM required a comprehensive time-calibrated ultrametric tree, we performed a divergence time estimate analysis using the 249-taxa $D_{nt,trans+mito,strict}$ with the same 11 fossil calibration points using MCMCTree as described in Section 1.8. See also Supplementary Figure 4B for overview of the fossil calibration points. Because of the computational limitation, we had to reduce the dataset containing only sites with unambiguous data for at least 80% of the 249 taxa. Because $D_{nt,trans+mito,strict}$ contained both transcriptome and mitochondrial genome data, of which only 60 taxa had complete data, the reduction method effectively created a dataset consisting of mitochondrial genome data only. The divergence time estimates for major nodes using this dataset were older than those using the reduced $D_{aa,trans,strict}$, but the relatively branch lengths and time estimates were consistent

across all major lineages. We think that the differences in divergence times could be potentially due to the differences in the datasets.
Our taxon sampling for Orthoptera contained less than 1% of overall species diversity, and our taxon sampling was not proportional to the number of known species for each lineage. For example, we included many Pyrgomorphidae species, although it is a relatively small family. To accurately represent species diversity and to account for incomplete taxon sampling, we specified sampling fraction for each family based on the number of described species recorded in the Orthoptera Species File[188]. We set priors using setBAMMpriors function in BAMMtools before the analysis. The priors used for the analysis were: expectedNumberOfShifts = 1.0; lambdaInitPrior = 17.0512659943593; lambdaShiftPrior = 0.00279913753644403; muInitPrior = 17.0512659943593; lambdaIsTimeVariablePrior = 0. We used "speciationextinction" as a model for the diversification analysis in BAMM, and ran for 10 million generations. Convergence assessment, analysis of rate shifts, and calculation of clade-specific rates were performed using BAMMtools. The files used for this analysis can be found in Supplementary Archive 3.

Trait-dependent diversification analysis

To test whether the evolution of hearing and sound production has affected speciation and extinction rates, we fitted models of trait-dependent diversification using the R package *hisse*[189]. Because it has been shown that the presence of unmeasured factors (or hidden states) could impact estimation of diversification rates for any observed trait when analysed under the framework of BiSSE (Binary State Speciation and Extinction) methods[190], we adopted a multimodel inference method, implemented in HiSSE (Hidden State Speciation and Extinction)[189]. We first pruned the time-calibrated ultrametric tree to only include Orthoptera (239 terminals), and used the binary character datasets for hearing and sound producing organs previously used for the Pagel's test. Because the presence of these organs in does not necessarily indicate the presence of acoustic communication, we created an additional dataset to code acoustic communication as a binary character to test if its evolution affected the diversification rate. The input data for the analysis is found in Supplementary Table 9. We fitted 24 different models, used in Beaulieu and O'Meara[189], to both the hearing dataset and the sound production dataset for Orthoptera. These models included four models corresponding to BiSSE models, four models corresponding to trait-independent models (described as CID models), and 16 models corresponding to different HiSSE models that assumed a hidden state associated with both the observed states. Detailed descriptions of these models are shown below. For all cases, we included the sampling fraction for the observed states in the models by calculating the proportion of the known 0's (i.e. absence) represented and the proportion of the known 1's (i.e. presence) represented in our tree. The resulting models were compared using AIC. All analyses were carried out in *hisse*. The files used for this analysis can be found in Supplementary Archive 3.

24 Models compared in HiSSE taken from Beaulieu and O'Meara[189]

**Model**
BiSSE: all free
BiSSE: ε0=ε1
BiSSE: q's equal
BiSSE: ε0=ε1, q's equal
CID-2: q's equal
CID-2: ε's, q's equal
CID-4: q's equal
CID-4: ε's equal, q's equal
HiSSE: q's equal
HiSSE: ε's equal, q's equal
HiSSE: τ0A=τ1A=τ0B, ε0A=ε1A=ε0B, q's equal
HiSSE: τ0A=τ1A=τ0B, ε's equal, q's equal
HiSSE: q0B1B=0, q1B0B=0, all other q's equal
HiSSE: ε's equal, q0B1B=0, q1B0B=0, all other q's equal
HiSSE: τ0A=τ1A=τ0B, ε0A=ε1A=ε0B, q0B1B=0,q1B0B=0, all other q's equal
HiSSE: τ0A=τ1A=τ0B, ε's equal, q0B1B=0, q1B0B=0, all other q's equal
HiSSE: τ0A=τ0B, ε0A=ε0B, q's equal
HiSSE: τ0A=τ0B, ε's equal, q's equal
HiSSE: τ0A=τ0B, ε0A=ε0B, q0B1B=0, q1B0B=0, all other q's equal
HiSSE: τ0A=τ0B, ε's equal, q0B1B=0, q1B0B=0, all other q's equal
HiSSE: τ0A=τ1A, ε0A=ε1A, q's equal
HiSSE: τ0A=τ1A, ε's equal, q's equal
HiSSE: τ0A=τ1A, ε0A=ε1A, q0B1B=0, q1B0B=0, all other q's equal
HiSSE: τ0A=τ1A, ε's equal, q0B1B=0, q1B0B=0, all other q's equal

Supplementary Figures

Supplementary Figure 1: Heat maps show pairwise Bowker's tests visualizing among-lineage heterogeneity as implemented in SymTest 2.0.47. P-values > 0.05 coloured in white indicate sequence pairs that fully match SRH conditions. SymTest was run for the complete transcriptome dataset on nucleotide level. Heat map of the nucleotide dataset including A) 1st codon position only B) 2nd codon position only, C) 3rd codon position only, D) 1st and 2nd codon position, and E) all codon positions. The nucleotide dataset only including the 2nd codon position (B) show less model violation compared to the other nucleotide datasets and were thus used for further downstream analyses.

A)

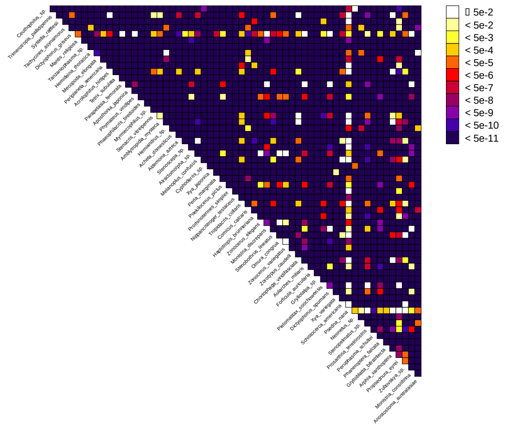

B)

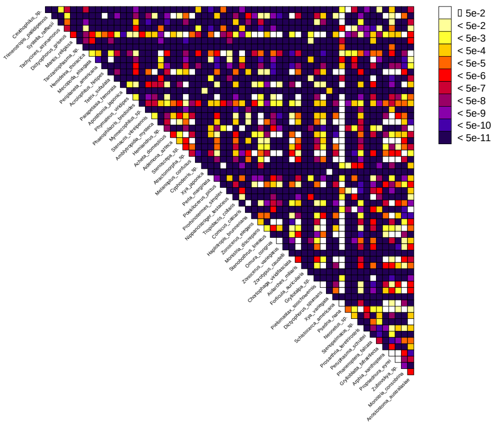

C)

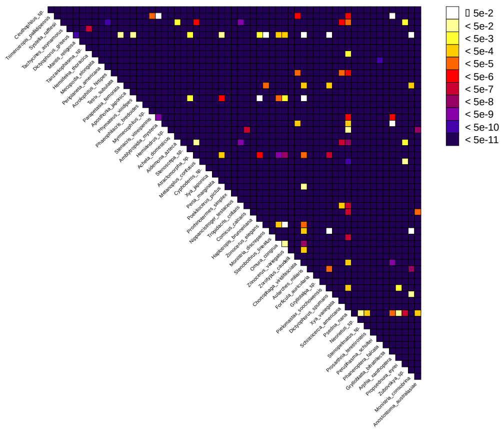

D)

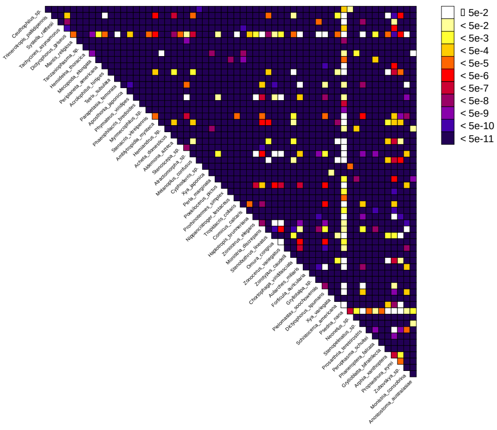

E)

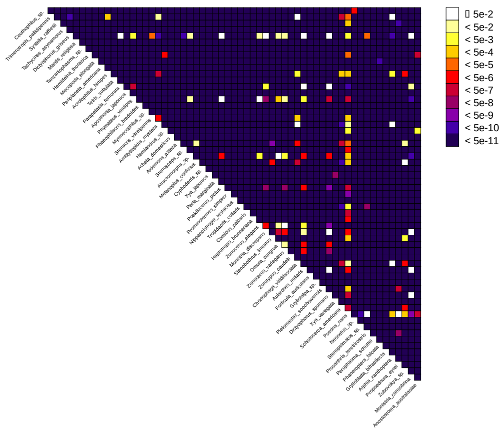

Supplementary Figure 2: Heat maps show species-pairwise site coverage as inferred with AliStat. A) Complete amino-acid transcriptome only datasets $D_{aa,trans,complete}$, B) complete nucleotide transcriptome dataset with the 2nd codon positions only $D_{nt,trans,complete}$, C) strict amino-acid transcriptome only datasets $D_{aa,trans,strict}$, D) strict nucleotide transcriptome dataset with the 2nd codon positions only $D_{nt,trans,strict}$, E) complete combined transcriptome and mitochondrial dataset $D_{nt,trans+mito,complete}$, F) strict combined transcriptome and mitochondrial dataset $D_{nt,trans+mito,strict}$. Low shared site coverage colored in shades of red; high shared site coverage in shades of green. Pairs of sequences, and completeness scores are provided in the text.

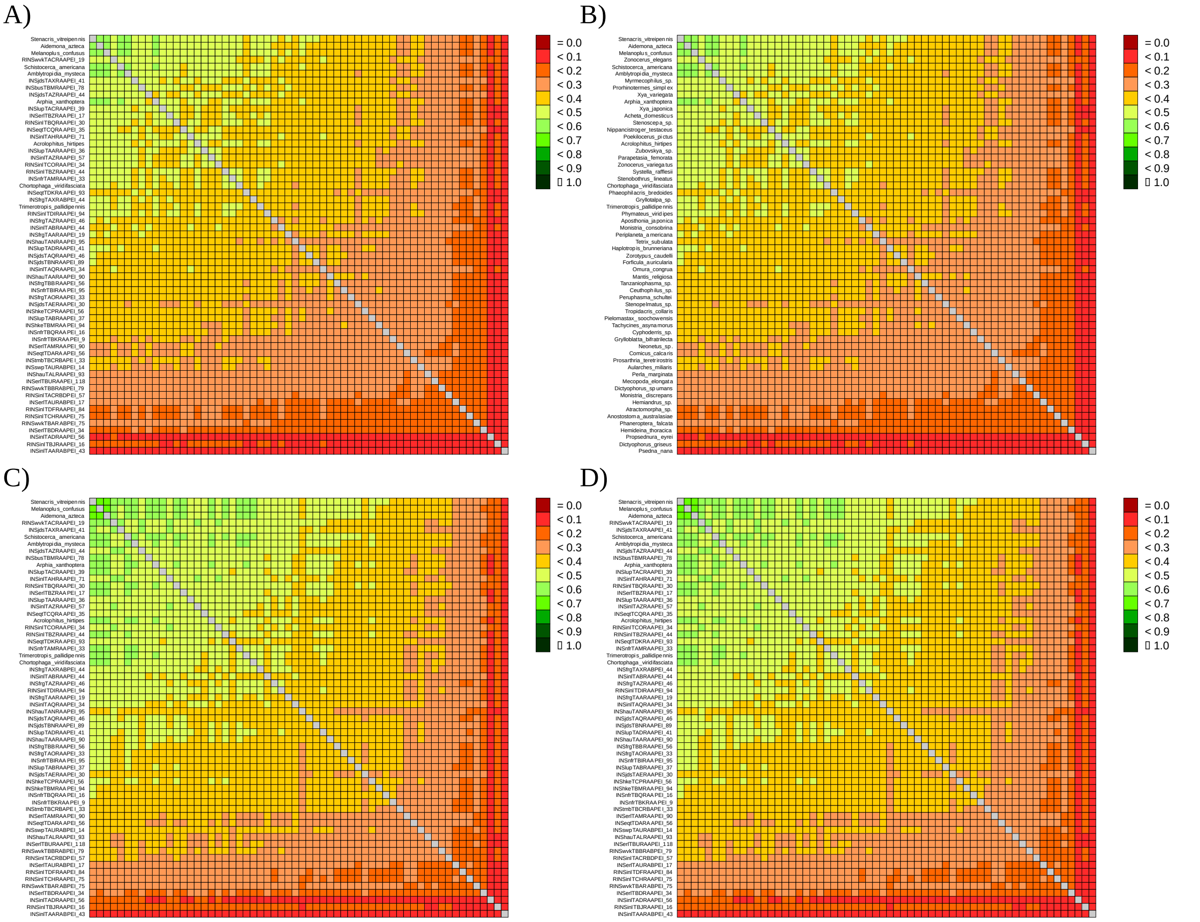

E)

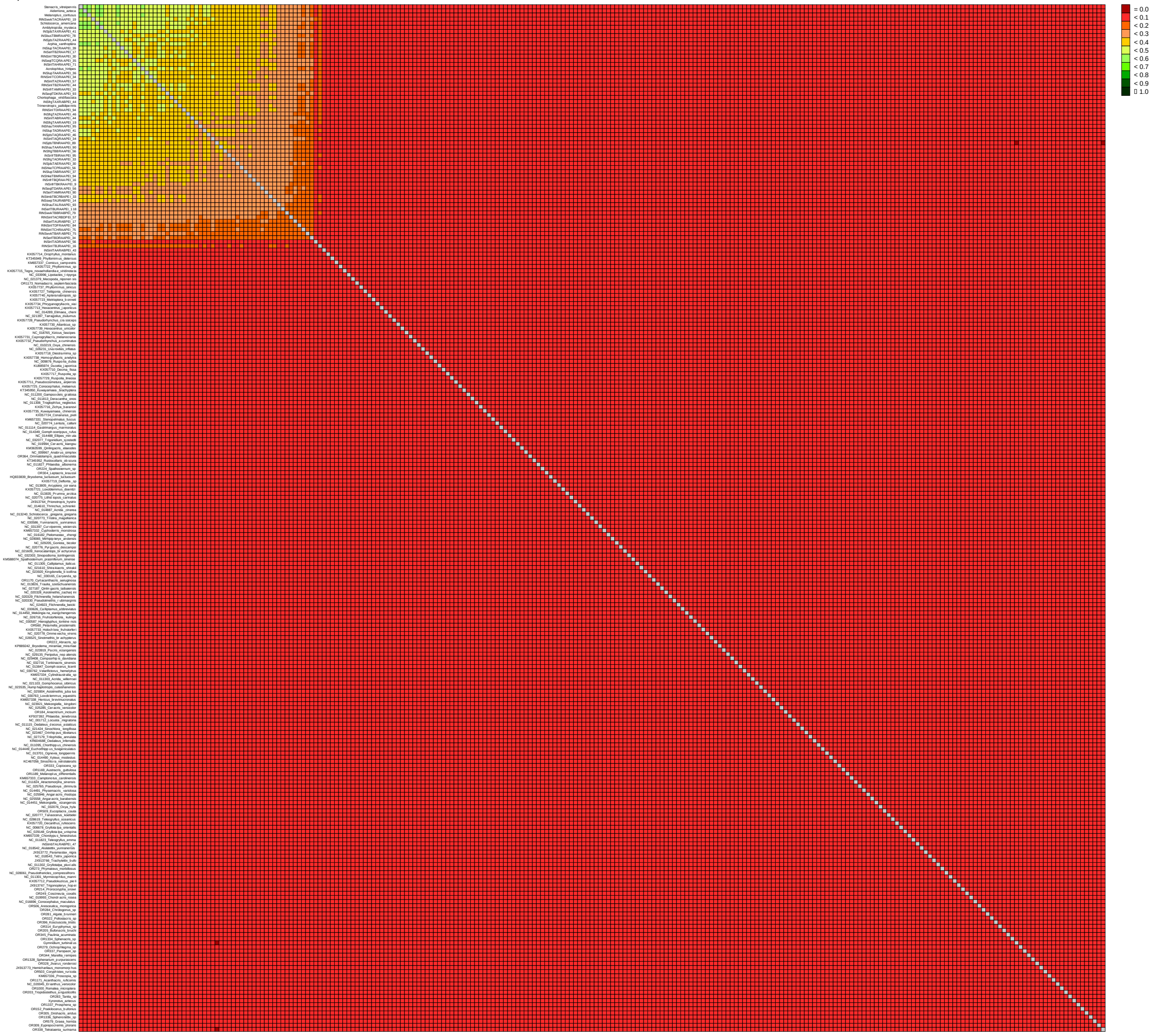

F)

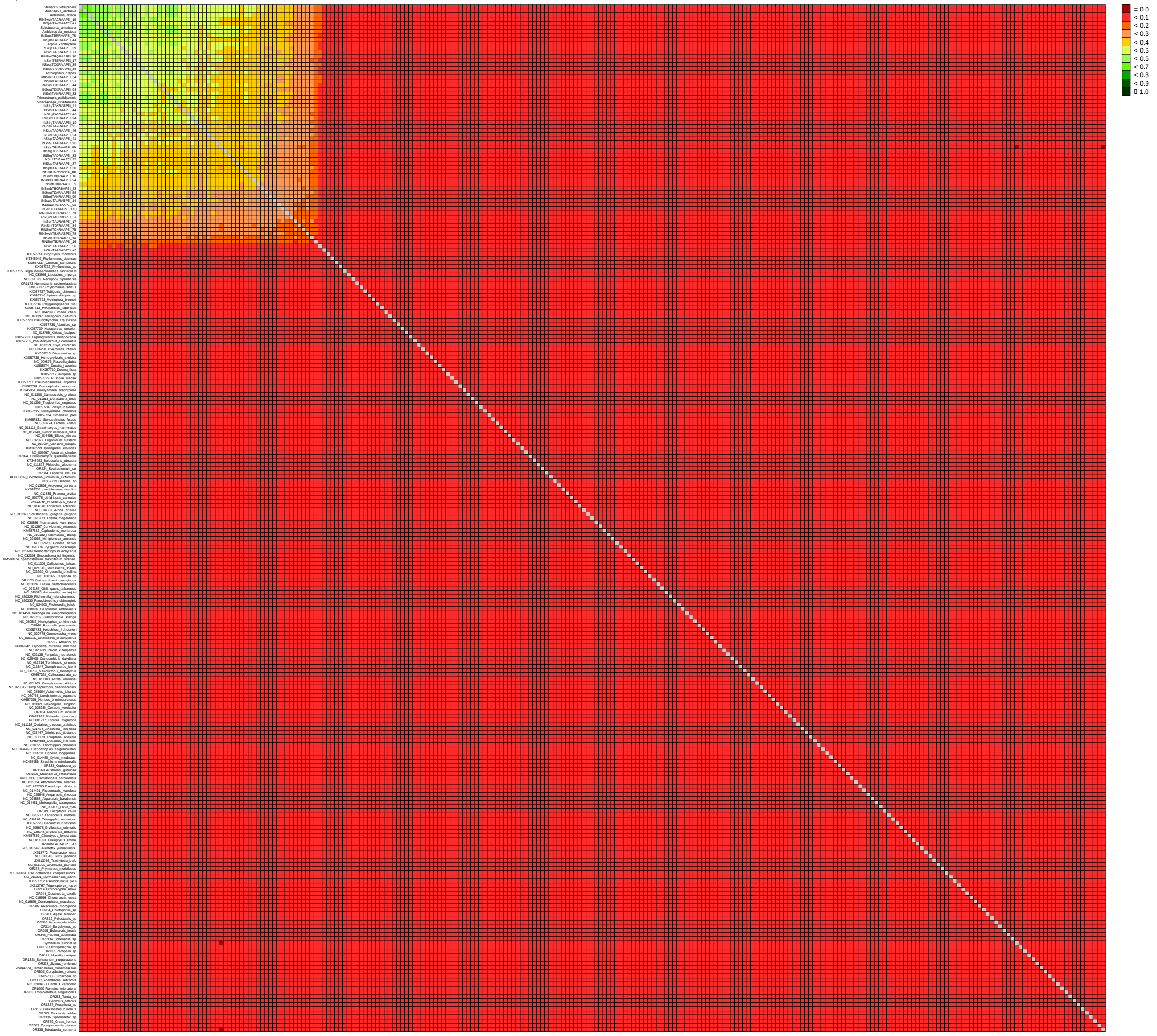

Supplementary Figure 3: Best ML tree (phylogram) inferred from the six datasets. A) $D_{aa,trans,complete}$ B) $D_{aa,trans,strict}$ C) $D_{nt,trans,complete}$ D) $D_{nt,trans,strict}$ E) $D_{nt,trans+mito,complete}$ F) $D_{nt,trans+mito,strict}$

A)

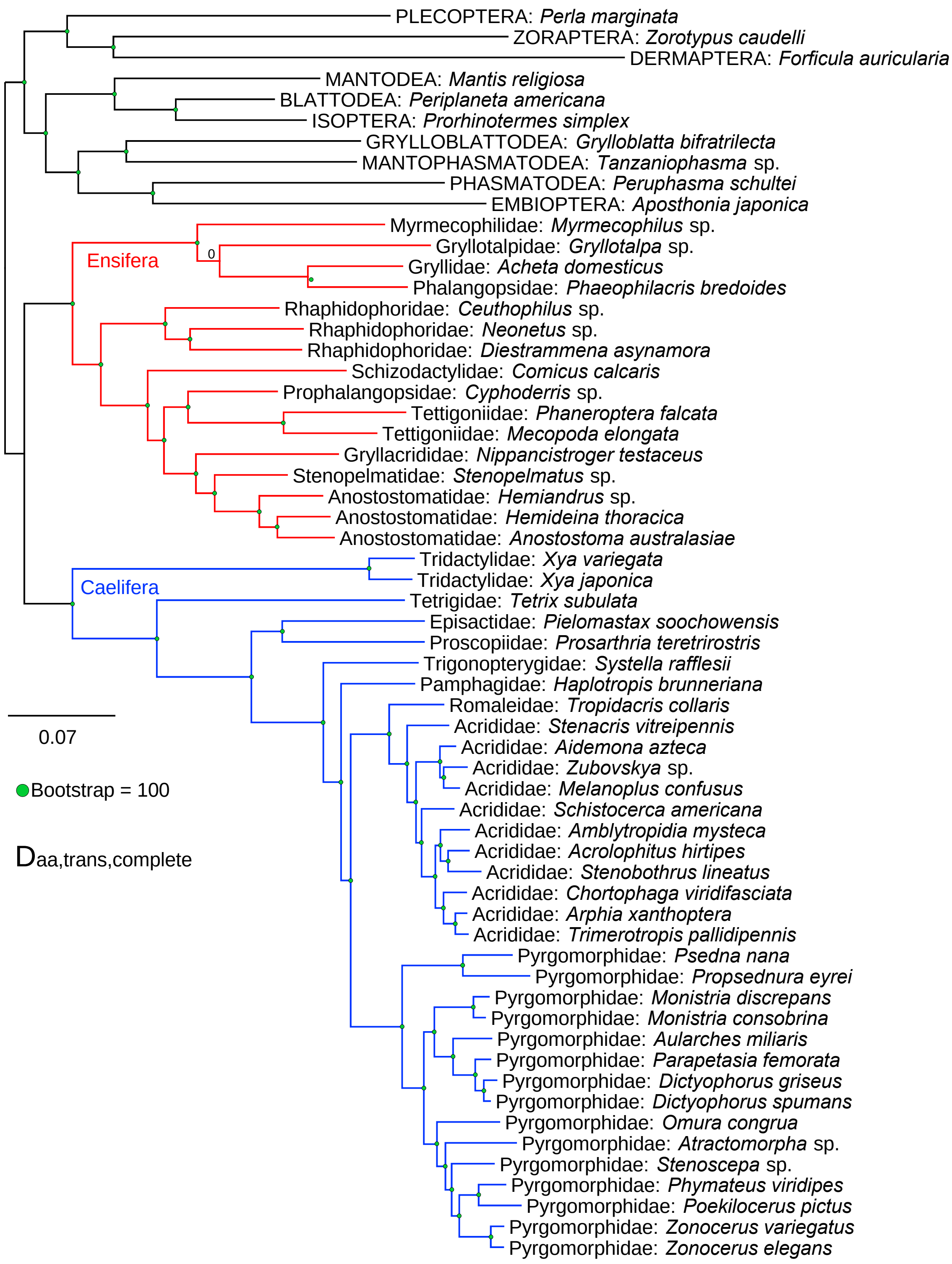

B)

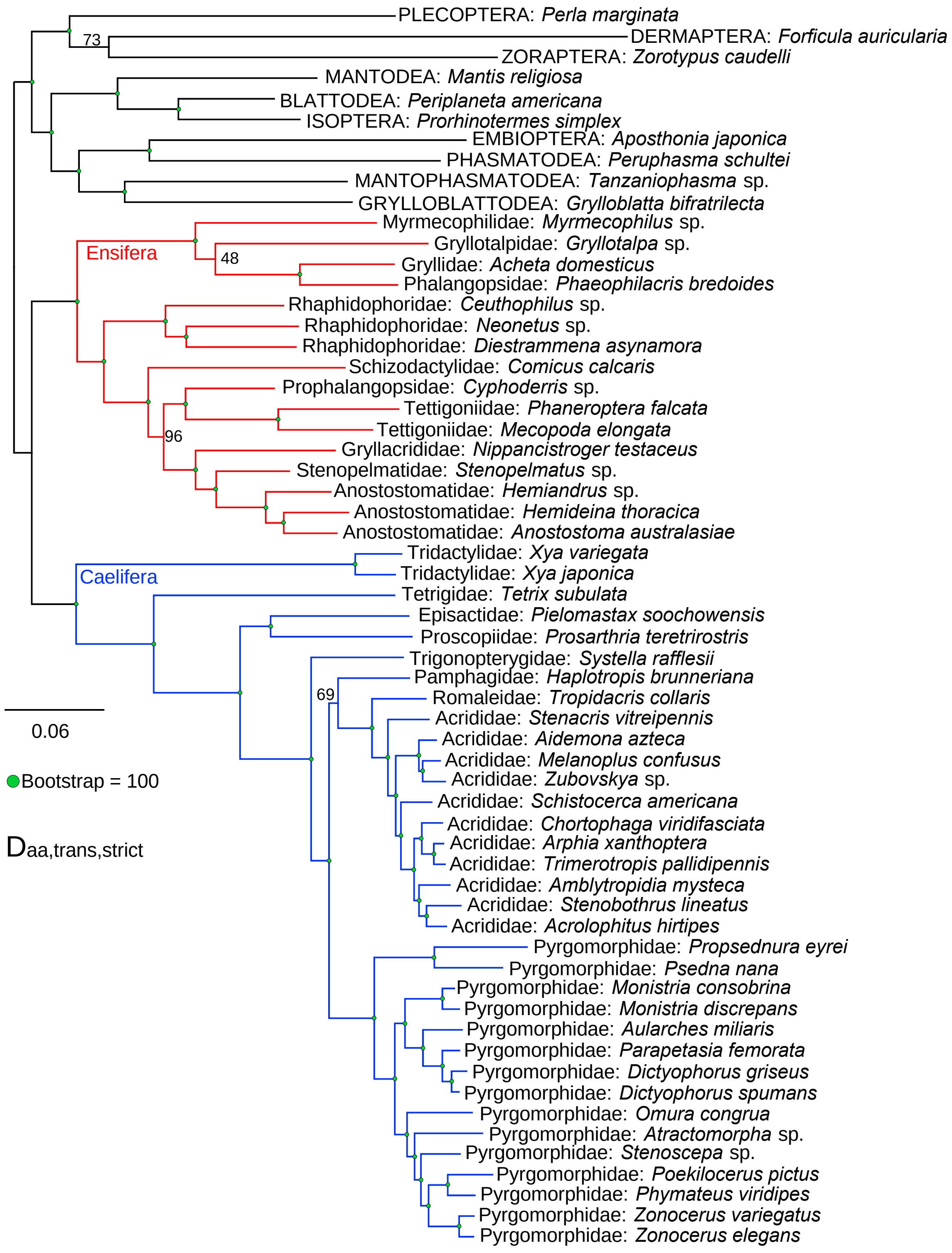

PLECOPTERA: *Perla marginata*
73
DERMAPTERA: *Forficula auricularia*
ZORAPTERA: *Zorotypus caudelli*
MANTODEA: *Mantis religiosa*
BLATTODEA: *Periplaneta americana*
ISOPTERA: *Prorhinotermes simplex*
EMBIOPTERA: *Aposthonia japonica*
PHASMATODEA: *Peruphasma schultei*
MANTOPHASMATODEA: *Tanzaniophasma* sp.
GRYLLOBLATTODEA: *Grylloblatta bifratrilecta*
Ensifera
Myrmecophilidae: *Myrmecophilus* sp.
48
Gryllotalpidae: *Gryllotalpa* sp.
Gryllidae: *Acheta domesticus*
Phalangopsidae: *Phaeophilacris bredoides*
Rhaphidophoridae: *Ceuthophilus* sp.
Rhaphidophoridae: *Neonetus* sp.
Rhaphidophoridae: *Diestrammena asynamora*
Schizodactylidae: *Comicus calcaris*
Prophalangopsidae: *Cyphoderris* sp.
Tettigoniidae: *Phaneroptera falcata*
96
Tettigoniidae: *Mecopoda elongata*
Gryllacrididae: *Nippancistroger testaceus*
Stenopelmatidae: *Stenopelmatus* sp.
Anostostomatidae: *Hemiandrus* sp.
Anostostomatidae: *Hemideina thoracica*
Anostostomatidae: *Anostostoma australasiae*
Caelifera
Tridactylidae: *Xya variegata*
Tridactylidae: *Xya japonica*
Tetrigidae: *Tetrix subulata*
Episactidae: *Pielomastax soochowensis*
Proscopiidae: *Prosarthria teretrirostris*
Trigonopterygidae: *Systella rafflesii*
Pamphagidae: *Haplotropis brunneriana*
69
Romaleidae: *Tropidacris collaris*
Acrididae: *Stenacris vitreipennis*
Acrididae: *Aidemona azteca*
Acrididae: *Melanoplus confusus*
Acrididae: *Zubovskya* sp.
Acrididae: *Schistocerca americana*
Acrididae: *Chortophaga viridifasciata*
Acrididae: *Arphia xanthoptera*
Acrididae: *Trimerotropis pallidipennis*
Acrididae: *Amblytropidia mysteca*
Acrididae: *Stenobothrus lineatus*
Acrididae: *Acrolophitus hirtipes*
Pyrgomorphidae: *Propsednura eyrei*
Pyrgomorphidae: *Psedna nana*
Pyrgomorphidae: *Monistria consobrina*
Pyrgomorphidae: *Monistria discrepans*
Pyrgomorphidae: *Aularches miliaris*
Pyrgomorphidae: *Parapetasia femorata*
Pyrgomorphidae: *Dictyophorus griseus*
Pyrgomorphidae: *Dictyophorus spumans*
Pyrgomorphidae: *Omura congrua*
Pyrgomorphidae: *Atractomorpha* sp.
Pyrgomorphidae: *Stenoscepa* sp.
Pyrgomorphidae: *Poekilocerus pictus*
Pyrgomorphidae: *Phymateus viridipes*
Pyrgomorphidae: *Zonocerus variegatus*
Pyrgomorphidae: *Zonocerus elegans*
0.06
Bootstrap = 100
Daa,trans,strict

C)

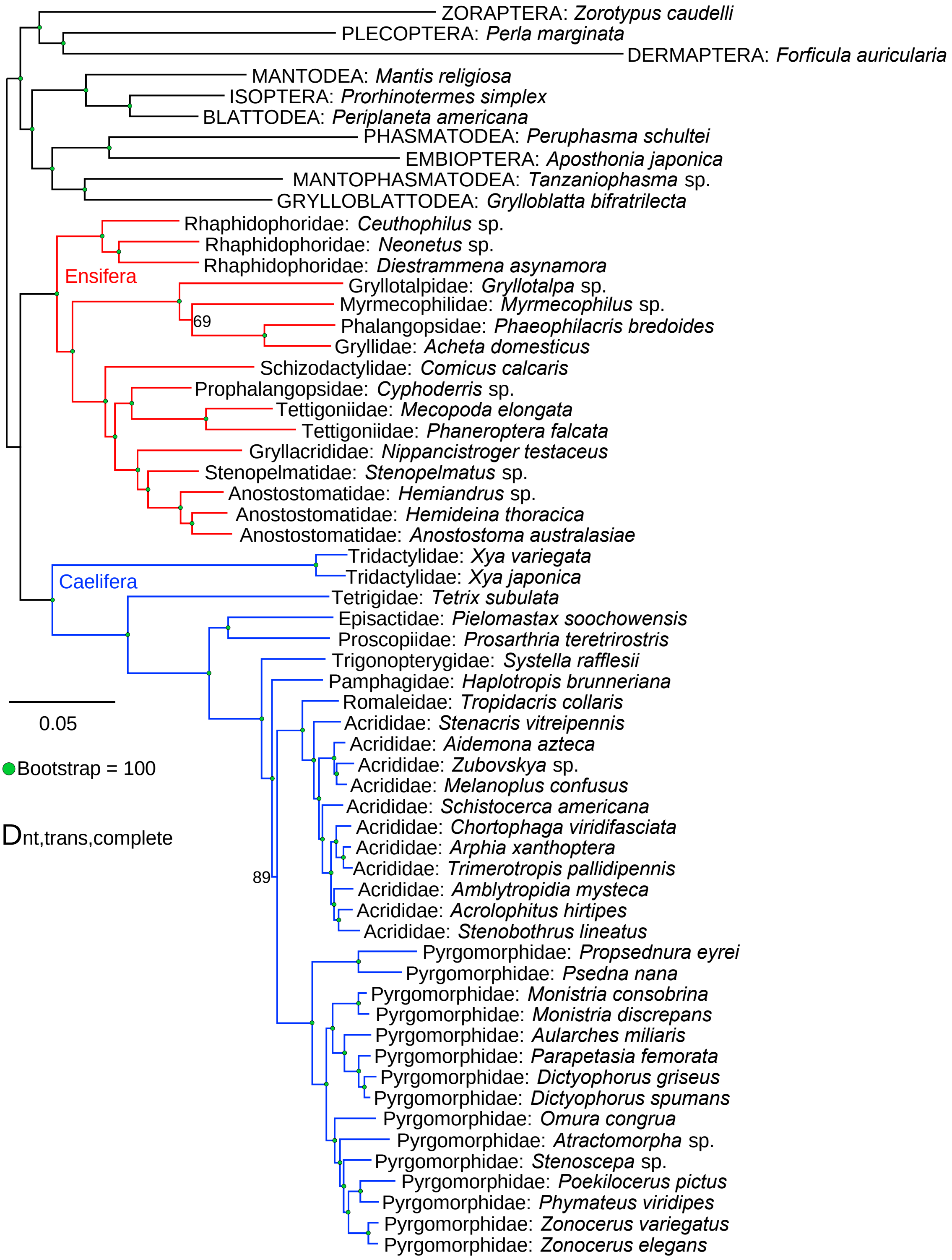

ZORAPTERA: *Zorotypus caudelli*
PLECOPTERA: *Perla marginata*
DERMAPTERA: *Forficula auricularia*
MANTODEA: *Mantis religiosa*
ISOPTERA: *Prorhinotermes simplex*
BLATTODEA: *Periplaneta americana*
PHASMATODEA: *Peruphasma schultei*
EMBIOPTERA: *Aposthonia japonica*
MANTOPHASMATODEA: *Tanzaniophasma* sp.
GRYLLOBLATTODEA: *Grylloblatta bifratrilecta*
Rhaphidophoridae: *Ceuthophilus* sp.
Rhaphidophoridae: *Neonetus* sp.
Rhaphidophoridae: *Diestrammena asynamora*
Ensifera
Gryllotalpidae: *Gryllotalpa* sp.
Myrmecophilidae: *Myrmecophilus* sp.
69
Phalangopsidae: *Phaeophilacris bredoides*
Gryllidae: *Acheta domesticus*
Schizodactylidae: *Comicus calcaris*
Prophalangopsidae: *Cyphoderris* sp.
Tettigoniidae: *Mecopoda elongata*
Tettigoniidae: *Phaneroptera falcata*
Gryllacrididae: *Nippancistroger testaceus*
Stenopelmatidae: *Stenopelmatus* sp.
Anostostomatidae: *Hemiandrus* sp.
Anostostomatidae: *Hemideina thoracica*
Anostostomatidae: *Anostostoma australasiae*
Tridactylidae: *Xya variegata*
Caelifera
Tridactylidae: *Xya japonica*
Tetrigidae: *Tetrix subulata*
Episactidae: *Pielomastax soochowensis*
Proscopiidae: *Prosarthria teretrirostris*
Trigonopterygidae: *Systella rafflesii*
Pamphagidae: *Haplotropis brunneriana*
Romaleidae: *Tropidacris collaris*
Acrididae: *Stenacris vitreipennis*
Acrididae: *Aidemona azteca*
Acrididae: *Zubovskya* sp.
Acrididae: *Melanoplus confusus*
Acrididae: *Schistocerca americana*
Acrididae: *Chortophaga viridifasciata*
Acrididae: *Arphia xanthoptera*
Acrididae: *Trimerotropis pallidipennis*
89
Acrididae: *Amblytropidia mysteca*
Acrididae: *Acrolophitus hirtipes*
Acrididae: *Stenobothrus lineatus*
Pyrgomorphidae: *Propsednura eyrei*
Pyrgomorphidae: *Psedna nana*
Pyrgomorphidae: *Monistria consobrina*
Pyrgomorphidae: *Monistria discrepans*
Pyrgomorphidae: *Aularches miliaris*
Pyrgomorphidae: *Parapetasia femorata*
Pyrgomorphidae: *Dictyophorus griseus*
Pyrgomorphidae: *Dictyophorus spumans*
Pyrgomorphidae: *Omura congrua*
Pyrgomorphidae: *Atractomorpha* sp.
Pyrgomorphidae: *Stenoscepa* sp.
Pyrgomorphidae: *Poekilocerus pictus*
Pyrgomorphidae: *Phymateus viridipes*
Pyrgomorphidae: *Zonocerus variegatus*
Pyrgomorphidae: *Zonocerus elegans*
0.05
Bootstrap = 100
Dnt,trans,complete

D)

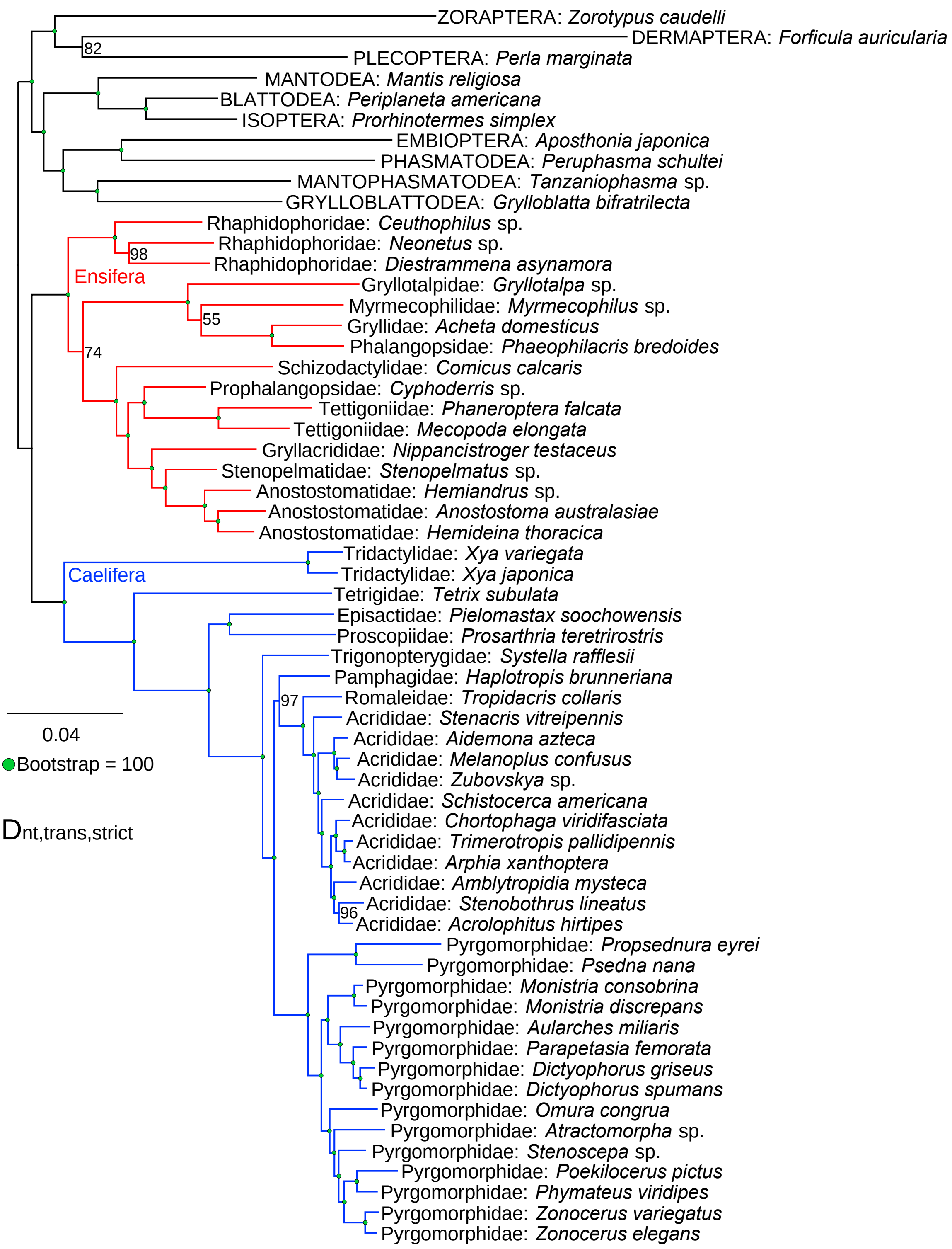

ZORAPTERA: *Zorotypus caudelli*
DERMAPTERA: *Forficula auricularia*
82
PLECOPTERA: *Perla marginata*
MANTODEA: *Mantis religiosa*
BLATTODEA: *Periplaneta americana*
ISOPTERA: *Prorhinotermes simplex*
EMBIOPTERA: *Aposthonia japonica*
PHASMATODEA: *Peruphasma schultei*
MANTOPHASMATODEA: *Tanzaniophasma* sp.
GRYLLOBLATTODEA: *Grylloblatta bifratrilecta*
Rhaphidophoridae: *Ceuthophilus* sp.
Rhaphidophoridae: *Neonetus* sp.
98
Rhaphidophoridae: *Diestrammena asynamora*
Ensifera
Gryllotalpidae: *Gryllotalpa* sp.
Myrmecophilidae: *Myrmecophilus* sp.
55
Gryllidae: *Acheta domesticus*
Phalangopsidae: *Phaeophilacris bredoides*
74
Schizodactylidae: *Comicus calcaris*
Prophalangopsidae: *Cyphoderris* sp.
Tettigoniidae: *Phaneroptera falcata*
Tettigoniidae: *Mecopoda elongata*
Gryllacrididae: *Nippancistroger testaceus*
Stenopelmatidae: *Stenopelmatus* sp.
Anostostomatidae: *Hemiandrus* sp.
Anostostomatidae: *Anostostoma australasiae*
Anostostomatidae: *Hemideina thoracica*
Tridactylidae: *Xya variegata*
Caelifera
Tridactylidae: *Xya japonica*
Tetrigidae: *Tetrix subulata*
Episactidae: *Pielomastax soochowensis*
Proscopiidae: *Prosarthria teretrirostris*
Trigonopterygidae: *Systella rafflesii*
Pamphagidae: *Haplotropis brunneriana*
97
Romaleidae: *Tropidacris collaris*
Acrididae: *Stenacris vitreipennis*
Acrididae: *Aidemona azteca*
Acrididae: *Melanoplus confusus*
Acrididae: *Zubovskya* sp.
Acrididae: *Schistocerca americana*
Acrididae: *Chortophaga viridifasciata*
Acrididae: *Trimerotropis pallidipennis*
Acrididae: *Arphia xanthoptera*
Acrididae: *Amblytropidia mysteca*
Acrididae: *Stenobothrus lineatus*
96
Acrididae: *Acrolophitus hirtipes*
Pyrgomorphidae: *Propsednura eyrei*
Pyrgomorphidae: *Psedna nana*
Pyrgomorphidae: *Monistria consobrina*
Pyrgomorphidae: *Monistria discrepans*
Pyrgomorphidae: *Aularches miliaris*
Pyrgomorphidae: *Parapetasia femorata*
Pyrgomorphidae: *Dictyophorus griseus*
Pyrgomorphidae: *Dictyophorus spumans*
Pyrgomorphidae: *Omura congrua*
Pyrgomorphidae: *Atractomorpha* sp.
Pyrgomorphidae: *Stenoscepa* sp.
Pyrgomorphidae: *Poekilocerus pictus*
Pyrgomorphidae: *Phymateus viridipes*
Pyrgomorphidae: *Zonocerus variegatus*
Pyrgomorphidae: *Zonocerus elegans*
0.04
Bootstrap = 100
Dnt,trans,strict

E)

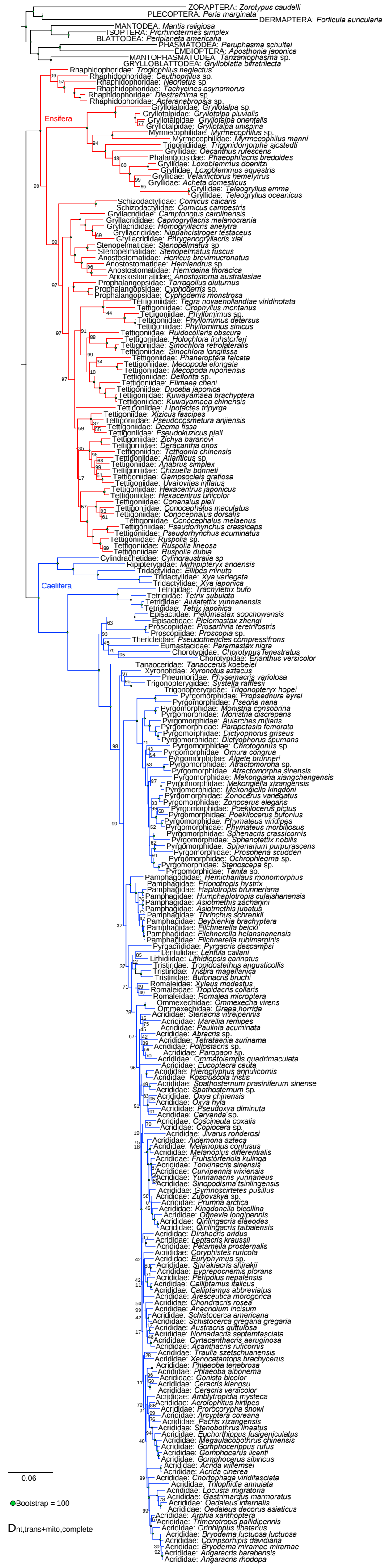

ZORAPTERA: Zorotypus caudelli
PLECOPTERA: Perla marginata
DERMAPTERA: Forficula auricularia
MANTODEA: Mantis religiosa
ISOPTERA: Prorhinotermes simplex
BLATTODEA: Periplaneta americana
PHASMATODEA: Peruphasma schultei
EMBIOPTERA: Aposthonia japonica
MANTOPHASMATODEA: Tanzaniophasma sp.
GRYLLOBLATTODEA: Grylloblatta bifratrilecta
Ensifera
Rhaphidophoridae: Troglophilus neglectus
Rhaphidophoridae: Ceuthophilus sp.
Rhaphidophoridae: Neonetus sp.
Rhaphidophoridae: Tachycines asynamorus
Rhaphidophoridae: Diestrammena sp.
Rhaphidophoridae: Apteranabropsis sp.
Gryllotalpidae: Gryllotalpa sp.
Gryllotalpidae: Gryllotalpa pluvialis
Gryllotalpidae: Gryllotalpa orientalis
Gryllotalpidae: Gryllotalpa unispina
Myrmecophilidae: Myrmecophilus sp.
Myrmecophilidae: Myrmecophilus manni
Trigonidiidae: Trigonidomorpha sjostedti
Gryllidae: Oecanthus rufescens
Phalangopsidae: Phaeophilacris bredoides
Gryllidae: Loxoblemmus doenitzi
Gryllidae: Loxoblemmus equestris
Gryllidae: Velarifictorus hemelytrus
Gryllidae: Acheta domesticus
Gryllidae: Teleogryllus emma
Gryllidae: Teleogryllus oceanicus
Schizodactylidae: Comicus calcaris
Schizodactylidae: Comicus campestris
Gryllacrididae: Camptonotus carolinensis
Gryllacrididae: Capnogryllacris melanocrania
Gryllacrididae: Homogryllacris anelytra
Gryllacrididae: Nippancistroger testaceus
Gryllacrididae: Phryganogryllacris xiai
Stenopelmatidae: Stenopelmatus sp.
Stenopelmatidae: Stenopelmatus fuscus
Anostostomatidae: Henicus brevimucronatus
Anostostomatidae: Hemiandrus sp.
Anostostomatidae: Hemideina thoracica
Anostostomatidae: Anostostoma australasiae
Prophalangopsidae: Tarragoilus diuturnus
Prophalangopsidae: Cyphoderris sp.
Prophalangopsidae: Cyphoderris monstrosa
Tettigoniidae: Tegra novaehollandiae viridinotata
Tettigoniidae: Orophyllus montanus
Tettigoniidae: Phyllomimus sp.
Tettigoniidae: Phyllomimus detersus
Tettigoniidae: Phyllomimus sinicus
Tettigoniidae: Ruidocollaris obscura
Tettigoniidae: Holochlora fruhstorferi
Tettigoniidae: Sinochlora retrolateralis
Tettigoniidae: Sinochlora longifissa
Tettigoniidae: Phaneroptera falcata
Tettigoniidae: Mecopoda elongata
Tettigoniidae: Mecopoda niponensis
Tettigoniidae: Deflorita sp.
Tettigoniidae: Elimaea cheni
Tettigoniidae: Ducetia japonica
Tettigoniidae: Kuwayamaea brachyptera
Tettigoniidae: Kuwayamaea chinensis
Tettigoniidae: Lipotactes tripyrga
Tettigoniidae: Xizicus fascipes
Tettigoniidae: Pseudocosmetura anjiensis
Tettigoniidae: Decma fissa
Tettigoniidae: Pseudokuzicus pieli
Tettigoniidae: Zichya baranovi
Tettigoniidae: Deracantha onos
Tettigoniidae: Tettigonia chinensis
Tettigoniidae: Atlanticus sp.
Tettigoniidae: Anabrus simplex
Tettigoniidae: Chizuella bonneti
Tettigoniidae: Gampsocleis gratiosa
Tettigoniidae: Uvarovites inflatus
Tettigoniidae: Hexacentrus japonicus
Tettigoniidae: Hexacentrus unicolor
Tettigoniidae: Conanalus pieli
Tettigoniidae: Conocephalus maculatus
Tettigoniidae: Conocephalus dorsalis
Tettigoniidae: Conocephalus melaenus
Tettigoniidae: Pseudorhynchus crassiceps
Tettigoniidae: Pseudorhynchus acuminatus
Tettigoniidae: Ruspolia sp.
Tettigoniidae: Ruspolia lineosa
Tettigoniidae: Ruspolia dubia
Caelifera
Cylindrachetidae: Cylindraustralia sp
Ripipterygidae: Mirhipipteryx andensis
Tridactylidae: Ellipes minuta
Tridactylidae: Xya variegata
Tridactylidae: Xya japonica
Tetrigidae: Trachytettix bufo
Tetrigidae: Tetrix subulata
Tetrigidae: Alulatettix yunnanensis
Tetrigidae: Tetrix japonica
Episactidae: Pielomastax soochowensis
Episactidae: Pielomastax zhengi
Proscopiidae: Prosarthria teretrirostris
Proscopiidae: Proscopia sp.
Thericleidae: Pseudothericles compressifrons
Eumastacidae: Paramastax nigra
Chorotypidae: Chorotypus fenestratus
Chorotypidae: Erianthus versicolor
Tanaoceridae: Tanaocerus koebelei
Xyronotidae: Xyronotus aztecus
Pneumoridae: Physemacris variolosa
Trigonopterygidae: Systella rafflesii
Trigonopterygidae: Trigonopteryx hopei
Pyrgomorphidae: Propsednura eyrei
Pyrgomorphidae: Psedna nana
Pyrgomorphidae: Monistria consobrina
Pyrgomorphidae: Monistria discrepans
Pyrgomorphidae: Aularches miliaris
Pyrgomorphidae: Parapetasia femorata
Pyrgomorphidae: Dictyophorus griseus
Pyrgomorphidae: Dictyophorus spumans
Pyrgomorphidae: Chlorizeina sp.
Pyrgomorphidae: Omura congrua
Pyrgomorphidae: Algete brunneri
Pyrgomorphidae: Atractomorpha sp.
Pyrgomorphidae: Atractomorpha sinensis
Pyrgomorphidae: Mekongiana xiangchengensis
Pyrgomorphidae: Mekongiella xizangensis
Pyrgomorphidae: Mekongiella kingdoni
Pyrgomorphidae: Zonocerus variegatus
Pyrgomorphidae: Zonocerus elegans
Pyrgomorphidae: Poekilocerus pictus
Pyrgomorphidae: Poekilocerus bufonius
Pyrgomorphidae: Phymateus viridipes
Pyrgomorphidae: Phymateus morbillosus
Pyrgomorphidae: Sphenacris crassicornis
Pyrgomorphidae: Sphenotettix nobilis
Pyrgomorphidae: Sphenarium purpurascens
Pyrgomorphidae: Prosphena scudderi
Pyrgomorphidae: Ochrophlegma sp.
Pyrgomorphidae: Stenoscepa sp.
Pyrgomorphidae: Tanita sp.
Pamphagodidae: Hemicharilaus monomorphus
Pamphagidae: Prionotropis hystrix
Pamphagidae: Haplotropis brunneriana
Pamphagidae: Humphaplotropis culaishanensis
Pamphagidae: Asiotmethis zacharjini
Pamphagidae: Asiotmethis jubatus
Pamphagidae: Thrinchus schrenkii
Pamphagidae: Beybienkia brachyptera
Pamphagidae: Filchnerella beicki
Pamphagidae: Filchnerella helanshanensis
Pamphagidae: Filchnerella rubimarginis
Pyrgacrididae: Pyrgacris descampsi
Lentulidae: Lentula callani
Lithidiidae: Lithidiopsis carinatus
Tristiridae: Tropidostethus angusticollis
Tristiridae: Tristira magellanica
Tristiridae: Bufonacris bruchi
Romaleidae: Xyleus modestus
Romaleidae: Tropidacris collaris
Romaleidae: Romalea microptera
Ommexechidae: Ommexecha virens
Ommexechidae: Graea horrida
Acrididae: Stenacris vitreipennis
Acrididae: Marellia remipes
Acrididae: Paulinia acuminata
Acrididae: Abracris sp.
Acrididae: Tetrataenia surinama
Acrididae: Pollostacris sp.
Acrididae: Paropaon sp.
Acrididae: Ommatolampis quadrimaculata
Acrididae: Eucoptacra caulta
Acrididae: Hieroglyphus annulicornis
Acrididae: Kosciuscola tristis
Acrididae: Spathosternum prasiniferum sinense
Acrididae: Spathosternum sp.
Acrididae: Oxya chinensis
Acrididae: Oxya hyla
Acrididae: Pseudoxya diminuta
Acrididae: Caryanda sp.
Acrididae: Coscineuta coxalis
Acrididae: Copiocera sp.
Acrididae: Jivarus ronderosi
Acrididae: Aidemona azteca
Acrididae: Melanoplus confusus
Acrididae: Melanoplus differentialis
Acrididae: Fruhstorferiola kulinga
Acrididae: Tonkinacris sinensis
Acrididae: Curvipennis wixiensis
Acrididae: Yunnanacris yunnaneus
Acrididae: Sinopodisma tsinlingensis
Acrididae: Gymnoscirtetes pusillus
Acrididae: Zubovskya sp.
Acrididae: Prumna arctica
Acrididae: Kingdonella bicollina
Acrididae: Ognevia longipennis
Acrididae: Qinlingacris elaeodes
Acrididae: Qinlingacris taibaiensis
Acrididae: Dirshacris aridus
Acrididae: Leptacris kraussii
Acrididae: Petamella prosternalis
Acrididae: Coryphistes ruricola
Acrididae: Euryphymus sp.
Acrididae: Shirakiacris shirakii
Acrididae: Eyprepocnemis plorans
Acrididae: Peripolus nepalensis
Acrididae: Calliptamus italicus
Acrididae: Calliptamus abbreviatus
Acrididae: Aresceutica morogorica
Acrididae: Chondracris rosea
Acrididae: Anacridium incisum
Acrididae: Schistocerca americana
Acrididae: Schistocerca gregaria gregaria
Acrididae: Austracris guttulosa
Acrididae: Nomadacris septemfasciata
Acrididae: Cyrtacanthacris aeruginosa
Acrididae: Acanthacris ruficornis
Acrididae: Traulia szetschuanensis
Acrididae: Xenocatantops brachycerus
Acrididae: Phlaeoba tenebrosa
Acrididae: Phlaeoba albonema
Acrididae: Gonista bicolor
Acrididae: Ceracris kiangsu
Acrididae: Ceracris versicolor
Acrididae: Amblytropidia mysteca
Acrididae: Acrolophitus hirtipes
Acrididae: Prorocorypha snowi
Acrididae: Arcyptera coreana
Acrididae: Pacris xizangensis
Acrididae: Stenobothrus lineatus
Acrididae: Euchorthippus fusigeniculatus
Acrididae: Megaulacobothrus chinensis
Acrididae: Gomphoceripus rufus
Acrididae: Gomphocerus licenti
Acrididae: Gomphocerus sibiricus
Acrididae: Acrida willemsei
Acrididae: Acrida cinerea
Acrididae: Chortophaga viridifasciata
Acrididae: Trilophidia annulata
Acrididae: Locusta migratoria
Acrididae: Gastrimargus marmoratus
Acrididae: Oedaleus infernalis
Acrididae: Oedaleus decorus asiaticus
Acrididae: Arphia xanthoptera
Acrididae: Trimerotropis pallidipennis
Acrididae: Orinhippus tibetanus
Acrididae: Bryodema luctuosa luctuosa
Acrididae: Compsorhipis davidiana
Acrididae: Bryodema miramae miramae
Acrididae: Angaracris barabensis
Acrididae: Angaracris rhodopa
0.06
Bootstrap = 100
Dnt,trans+mito,complete

F)

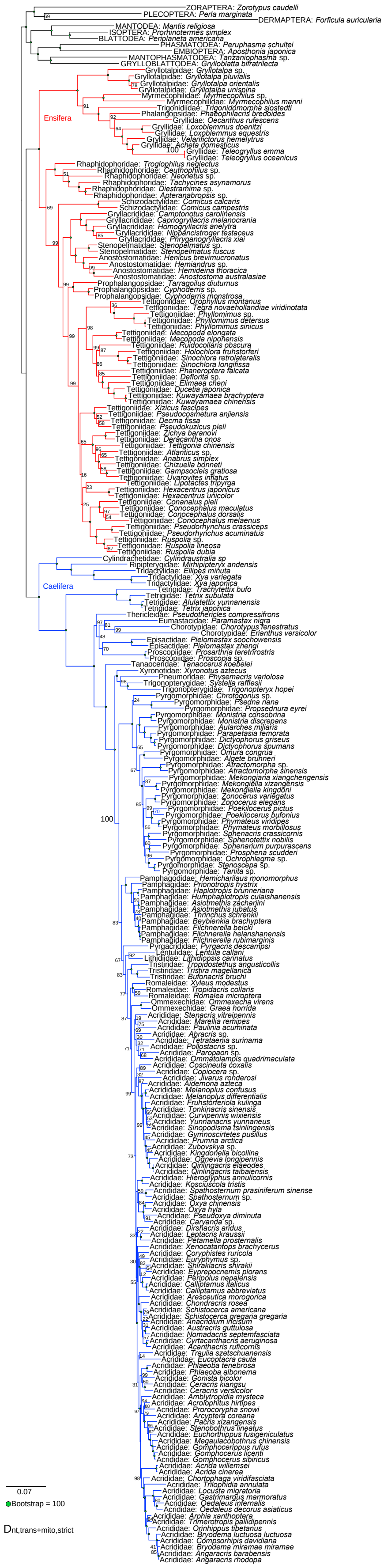

Supplementary Figure 4: Fossil calibrations used for estimating divergence dates of Orthoptera. Numbered circles represent fossils used for calibration and their approximate placement on the phylogenetic tree. Ranges (coloured boxes connected by dashed arrows) include minimum and maximum ages for fossils with the corresponding colour. Further details are given in Supplementary Table 7. A) Fossil calibration points for dataset $D_{aa,trans,strict}$ B) Fossil calibration points for dataset $D_{nt,trans+mito,strict}$.

A)

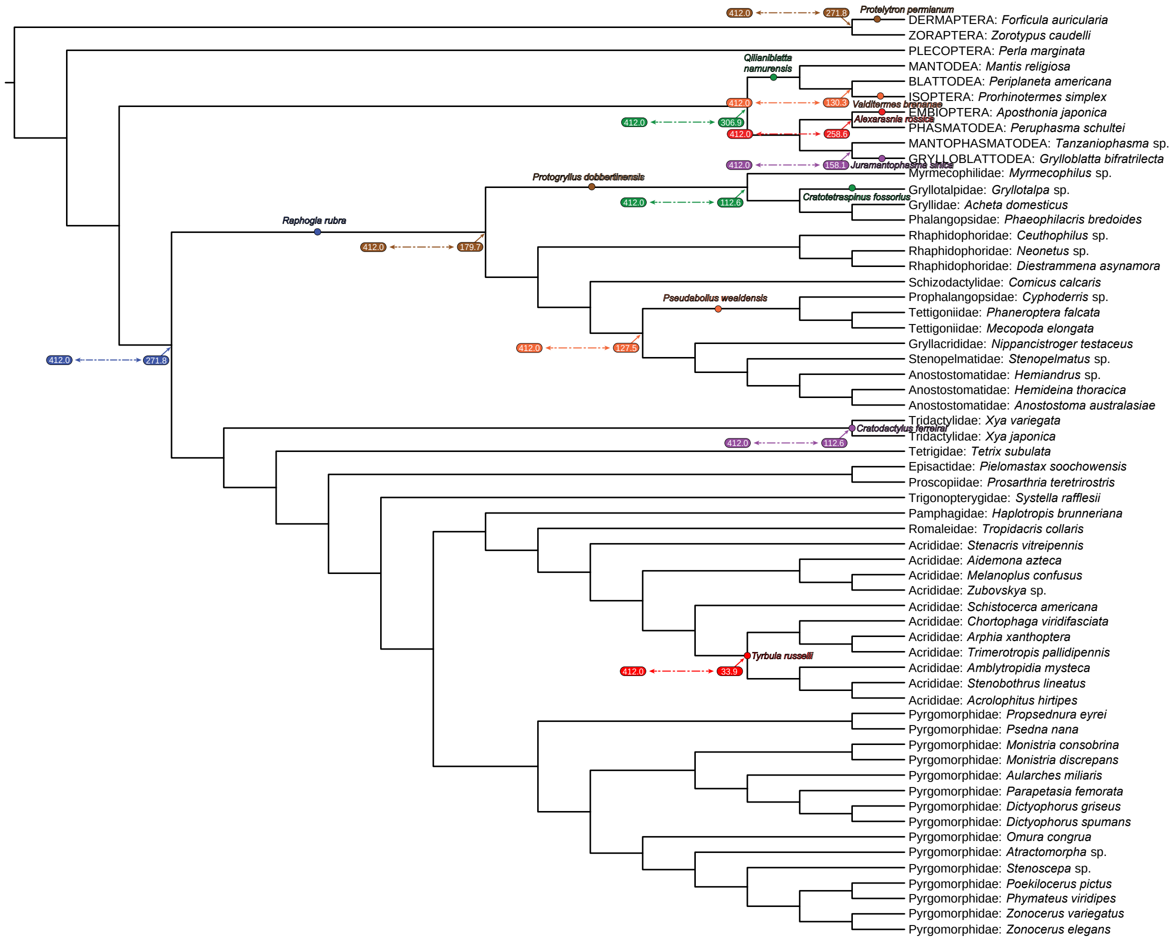

B)

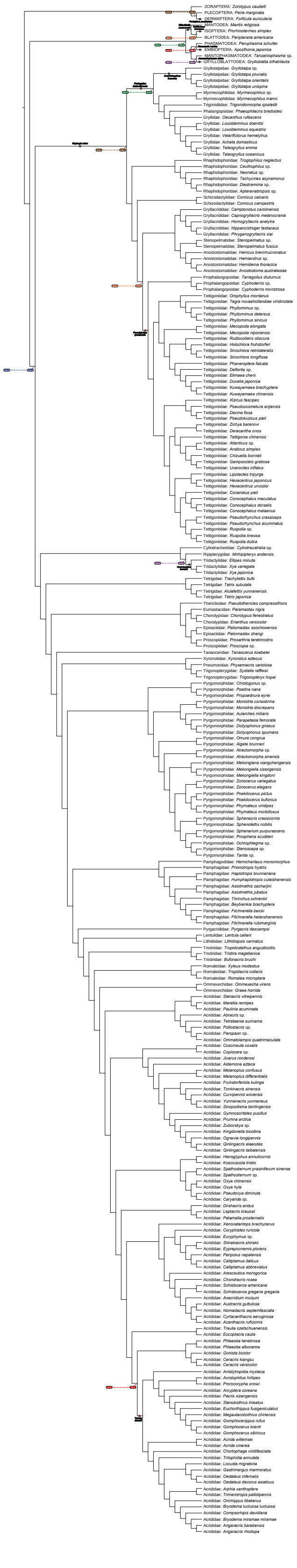

Supplementary Figure 5: Pairwise comparison of posterior mean node age estimates and upper and lower confidence intervals (CI) of four independent runs of the reduced strict transcriptome only dataset ($D_{aa,trans,strict}$). Runs were performed with the independent-rates clock model and identical settings expect for the seed. Black dots: posterior mean ages; +: lower 95% equal-tail CI; triangles: 95% upper equal-tail CI.

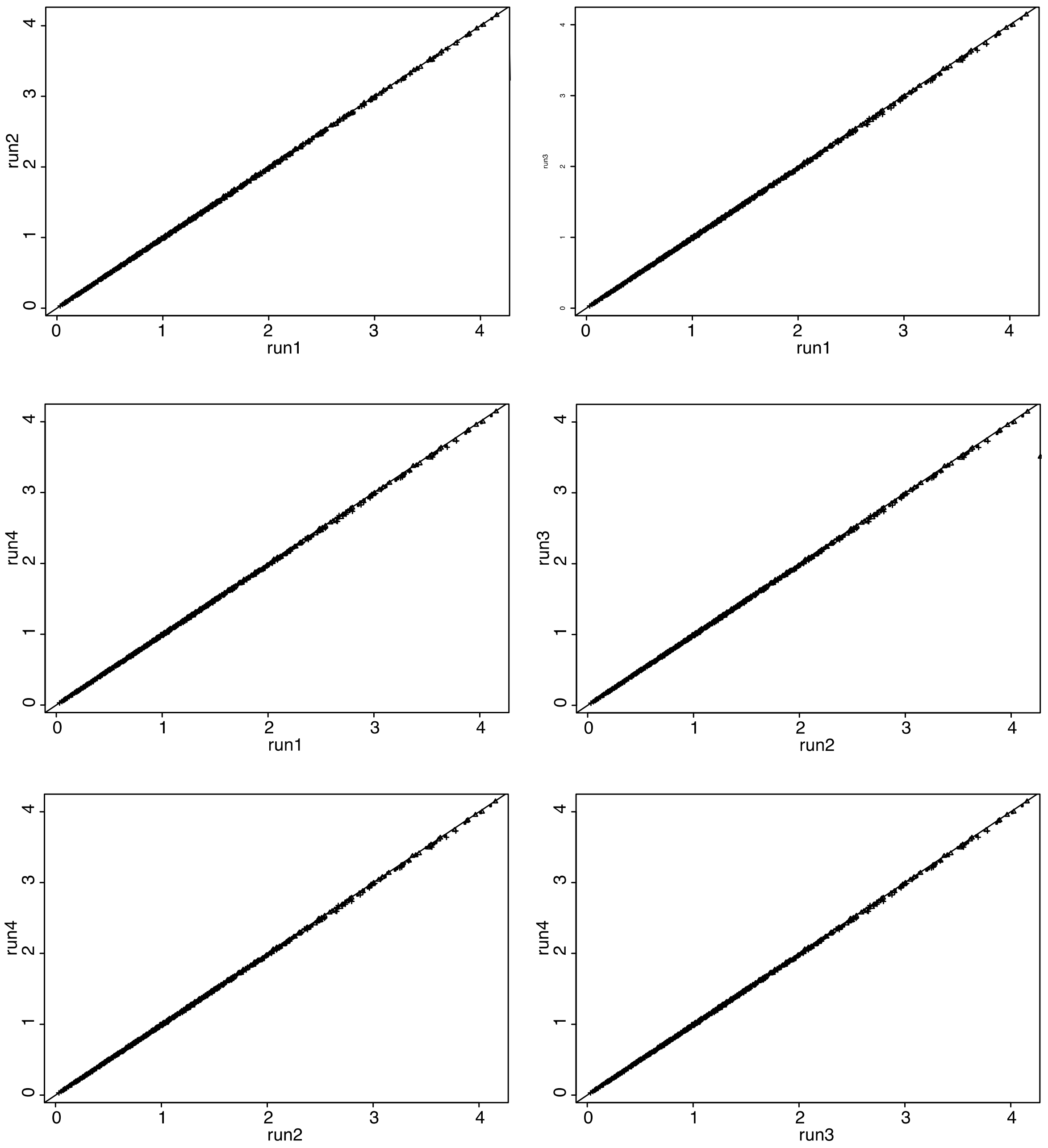

Supplementary Figure 6: Interpretation of P*seudaboilus wealdensis* Gorochov, Jarzembowski & Coram, 2006 following terminology and color-coding adopted in Chivers et al. (2017)[158] (paratype BMB 018617, Booth Museum of Natural History, Brighton, UK; left forewing, negative imprint, flipped horizontally; original photograph reproduced with permission from the author, E. Jarzembowski, and from the editorial board of *Cretaceous Research*). A. Wing venation homologies. B. Area homologies.

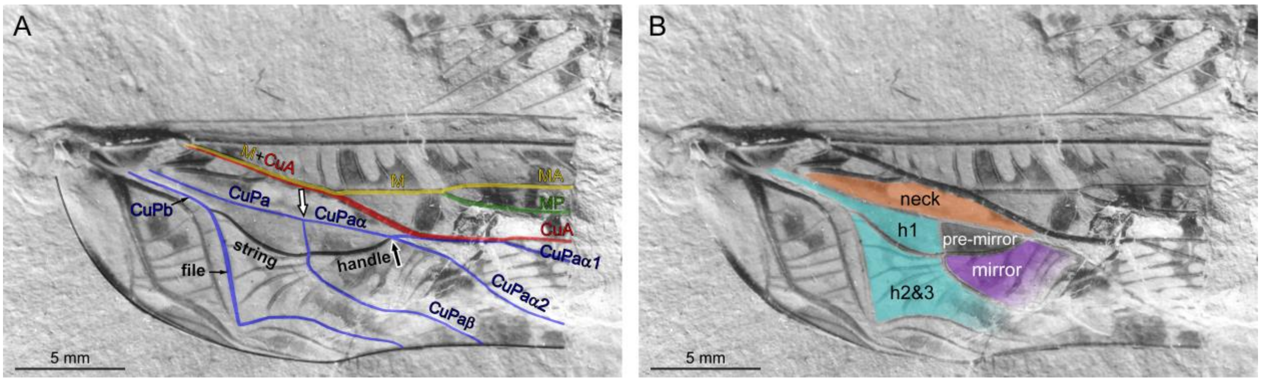

Supplementary Note: Archive files and descriptions provided via the Digital Repository DRYAD

Files can be found here: https://doi.org/10.5061/dryad.qjq2bvqc6

Supplementary Archive 1.

This archive contains files relevant for the ortholog prediction using Orthograph. [Supplementary_Archive_1.zip: 229KB, md5sum:19c6d308e3691de43a45859eee677584]

This archive includes a small readme and a table with all orthologous sequence groups (OGs) as used in this study from the OrthoDB v7 database. The official gene sets can be downloaded from OrthoDB v7 (ftp://cegg.unige.ch/OrthoDB7/FASTA/).

Supplementary Archive 2.

This archive contains all files relevant to the phylogenetic analyses. [Supplementary_Archive_2.zip: 60.29MB, md5sum:18605ae178b597a0a377baab88804301]

This archive includes six supermatrices (PHYLIP format) and respective partition files (NEXUS format) including selected models (*partitions.nex, assigned models to each partition are listed at the bottom). The partitioning is based on protein domains annotation for the transcriptomic partitions and on 1st, 2nd, and 3rd nucleotide position for the mitochondrial genome partitions.

- $D_{aa,trans,complete}$: complete amino-acid transcriptome only datasets + corresponding partition file including selected models (see Supplementary Figure S3A)
- $D_{aa,trans,strict}$: strict amino-acid transcriptome only datasets + corresponding partition file including selected models (see Supplementary Figure S3B)
- $D_{nt,trans,complete}$: complete nucleotide transcriptome dataset with the 2nd codon positions only decisive supermatrix on nucleotide level including only 2nd codon positions + corresponding partition file including selected models (see Supplementary Figure S3C)
- $D_{nt,trans,strict}$: strict nucleotide transcriptome dataset with the 2nd codon positions only + corresponding partition file including selected models (see Supplementary Figure S3D)
- $D_{nt,trans+mito,complete}$: complete combined transcriptome and mitochondrial dataset on nucleotide level + corresponding partition file including selected models (see Supplementary Figure S3E)
- $D_{nt,trans+mito,strict}$: strict combined transcriptome and mitochondrial dataset on nucleotide level + corresponding partition file including selected models (see Supplementary Figure S3F)

Supplementary Archive 3.

This archive contains all files relevant to the divergence time estimations. [Supplementary_Archive_3.zip: 2.76 MB, md5sum:c4dac19d5a3585e86e71d20c699e700a]

- Orthoptera_calibrations.tre: tree with minimum and maximum prior age calibrations (NEWICK format as required for MCMCTree), see Supplementary Figure S4A.
- Orthoptera_mcmctree.ctl: MCMCTree control file (example filenames) for the main analyses after generation of the Hessian matrix. Note that for all four runs parameters were kept identical.
- C80_D_aa_trans_strict.phy: reduced strict amino-acid transcriptome only datasets (i.e. "reduced $D_{aa,trans,strict}$") (PHYLIP format). See Supplementary Information S1.8. For resulting divergence dates see Figure 1.
- Subdirectory "dated_trees": Chronograms (NEWICK format) inferred from the reduced strict amino-acid transcriptome only datasets "reduced $D_{aa,trans,strict}$", 4 replicates;). One randomly selected the chronogram was arbitrarily chosen as the representative for dates reported in our study (also see Supplementary Information S1.8.). Files: FigTree_C80_run1.tre; FigTree_C80_run2.tre; FigTree_C80_run3.tre; FigTree_C80_run4.tre

Supplementary Archive 4.

This archive contains all files relevant to the phylogenetic comparative methods, including Pagel's test, BAMM, and HiSSE. [Supplementary_Archive_4.zip: 11.1MB, md5sum:d87742e982f3b278b34575f5183cf040]

- Subdirectory "Pagel_Files" contains the following files: Page.R (R code used for running the analysis), character_binary.csv (binary trait data), Orthoptera_only_ML.tre (pruned tree for Orthoptera), Ensifera_ML.tre (pruned tree for Ensifera), Caelifera_ML.tre (pruned tree for Caelifera)
- Subdirectory "BAMM_Files" contains the following files: Orthoptera_bigtimetree.tre (ultrametric tree used for the analysis), sample_probs.txt (information regarding sampling frequency), diversification.txt (configuration file), output files (run_info.txt, mcmc_out.txt, event_data.txt, chain_swap.txt)
- Subdirectory "HiSSE_Files" contains the following files: R codes for running the analyses (HiSSE Hearing Organ.R, HiSSE Sound Producing Organ.R, HiSSE Acoustic Communication.R), Orthoptera_only_bigtimetree.tre (pruned ultrametric tree), data files for binary traits (character_binary_hearing.csv, character_binary_sound.csv, character_binary_acoustic.csv), result files (HiSSE Hearing Organ Result.txt, HiSSE Sound Producing Organ Result.txt, HiSSE Acoustic Communication Result.txt), HiSSE Result.txt (summary and AIC), Character reconstructions of states and net diversification rates mapped on to the best fitting trees (Hearing HiSSE.tif, Sound Production CID4.tif, Acoustic CID4.tif)

Supplementary References

1 Wipfler, B. *et al.* Evolutionary history of Polyneoptera and its implications for our understanding of early winged insects. *Proc Natl Acad Sci U S A* **116**, 3024-3029, doi:10.1073/pnas.1817794116 (2019).

2 Misof, B. *et al.* Phylogenomics resolves the timing and pattern of insect evolution. *Science* **346**, 763-767, doi:10.1126/science.1257570 (2014).

3 Peters, R. S. *et al.* Evolutionary History of the Hymenoptera. *Curr Biol* **27**, 1013-1018, doi:10.1016/j.cub.2017.01.027 (2017).

4 Xie, Y. *et al.* SOAPdenovo-Trans: de novo transcriptome assembly with short RNA-Seq reads. *Bioinformatics* **30**, 1660-1666, doi:10.1093/bioinformatics/btu077 (2014).

5 Mayer, C. *et al.* BaitFisher: A Software Package for Multispecies Target DNA Enrichment Probe Design. *Mol Biol Evol* **33**, 1875-1886, doi:10.1093/molbev/msw056 (2016).

6 Afgan, E. *et al.* The Galaxy platform for accessible, reproducible and collaborative biomedical analyses: 2018 update. *Nucleic Acids Research* **46**, W537-W544, doi:10.1093/nar/gky379 (2018).

7 Grabherr, M. G. *et al.* Full-length transcriptome assembly from RNA-Seq data without a reference genome. *Nat Biotechnol* **29**, 644-652, doi:10.1038/nbt.1883 (2011).

8 Langmead, B., Trapnell, C., Pop, M. & Salzberg, S. L. Ultrafast and memory-efficient alignment of short DNA sequences to the human genome. *Genome Biol* **10**, R25, doi:10.1186/gb-2009-10-3-r25 (2009).

9 Langmead, B. & Salzberg, S. L. Fast gapped-read alignment with Bowtie 2. *Nat Methods* **9**, 357-359, doi:10.1038/nmeth.1923 (2012).

10 Li, H. *et al.* The Sequence Alignment/Map format and SAMtools. *Bioinformatics* **25**, 2078-2079, doi:10.1093/bioinformatics/btp352 (2009).

11 Li, H. A statistical framework for SNP calling, mutation discovery, association mapping and population genetical parameter estimation from sequencing data. *Bioinformatics* **27**, 2987-2993, doi:10.1093/bioinformatics/btr509 (2011).

12 Song, H., Marino-Perez, R., Woller, D. A. & Cigliano, M. M. Evolution, Diversification, and Biogeography of Grasshoppers (Orthoptera: Acrididae). *Insect Syst Diver* **2**, 3; 1-25, doi:10.1093/isd/ixy008 (2018).

13 Chang, H. H. & Huang, Y. The complete mitochondrial genome of the *Hieroglyphus tonkinensis* (Orthoptera: Acrididae). *Mitochondrial DNA B* **1**, 534-535, doi:10.1080/23802359.2016.1197067 (2016).

14 Chen, Z. N. & Xu, S. Q. The complete mitochondrial DNA genome sequence of a terrestrial grasshopper, *Curvipennis wixiensis* (Acrididae: Podismini). *Conserv Genet Resour* **9**, 115-118, doi:10.1007/s12686-016-0634-8 (2017).

15 Ding, F.-M., Shi, H.-W. & Huang, Y. Complete Mitochondrial Genome and Secondary Structures of lrRNA and srRNA of *Atractomorpha sinensis* (Orthoptera, Pyrgomorphidae). *Zoological Research* **28**, 580-588 (2007).

16 Erler, S., Ferenz, H.-J., Moritz, R. F. A. & Kaatz, H.-H. Analysis of the mitochondrial genome of *Schistocerca gregaria gregaria* (Orthoptera: Acrididae). *Biological Journal of the Linnean Society* **99**, 296-305, doi:10.1111/j.1095-8312.2009.01365.x (2010).

17 Fenn, J. D., Cameron, S. L. & Whiting, M. F. The complete mitochondrial genome sequence of the Mormon cricket (*Anabrus simplex*: Tettigoniidae: Orthoptera) and an analysis of control region variability. *Insect Mol Biol* **16**, 239-252, doi:10.1111/j.1365-2583.2006.00721.x (2007).

18 Fenn, J. D., Song, H., Cameron, S. L. & Whiting, M. F. A preliminary mitochondrial genome phylogeny of Orthoptera (Insecta) and approaches to maximizing phylogenetic signal found within mitochondrial genome data. *Mol Phylogenet Evol* **49**, 59-68, doi:10.1016/j.ympev.2008.07.004 (2008).

19 Flook, P. K., Rowell, C. H. & Gellissen, G. The sequence, organization, and evolution of the *Locusta migratoria* mitochondrial genome. *J Mol Evol* **41**, 928-941, doi:10.1007/bf00173173 (1995).

20 Gao, J., Cheng, C. H. & Huang, Y. Sequence analysis of complete mitochondrial DNA of *Gomphocerus licenti*. *Zoological Research* **6**, 603-612 (2009).

21 Guan, B., Guo, H. F. & Zhou, Z. J. Illumina next-generation sequencing reveals the mitochondrial genome of *Ducetia japonica* (Orthoptera: Tettigoniidae). *Mitochondrial DNA B* **1**, 361-362, doi:10.1080/23802359.2016.1168717 (2016).

22 Guan, D. L. & Xu, S. Q. Complete mitochondrial genome of the geophilous grasshopper *Trilophidia annulata* (Acrididae: Oedipodinae: Trilophidia). *Mitochondrial DNA A* **27**, 3143-3144, doi:10.3109/19401736.2015.1007304 (2016).

23 Guo, C. N., Zhang, Q. & Huang, Y. The complete mitochondrial genome of the *Oedaleus infernalis sauss* (Orthoptera: Oedipodidae). *Mitochondrial DNA A* **28**, 89-90, doi:10.3109/19401736.2015.1110812 (2017).

24 Han, H., Zhou, X. & Pang, B. The complete mitochondrial genome of *Angaracris barabensis* Pallas (Orthoptera: Acridoidea). *Mitochondrial DNA A DNA Mapp Seq Anal* **27**, 1827-1828, doi:10.3109/19401736.2014.971244 (2016).
25 Han, H. B., Wang, N., Xu, L. B., Gao, S. J. & Liu, A. P. The complete mitochondrial genome of *Calliptamus abbreviatus* Ikovnnikov (Orthoptera: acridoidea). *Mitochondrial DNA B* **1**, 770-771, doi:10.1080/23802359.2016.1238754 (2016).
26 Han, H. B., Zhou, X. R. & Pang, B. P. The complete mitochondrial genome of *Angaracris rhodopa* Fischer & Walheim (Orthoptera: Acridoidea). *Mitochondrial DNA A* **27**, 2127-2128, doi:10.3109/19401736.2014.982589 (2016).
27 Hao, J., Liu, N., Zhou, F. & Huang, Y. The complete mitochondrial genome of *Bryodema miramae* (Orthoptera: Oedipodidae). *Mitochondrial DNA A* **27**, 2500-2501, doi:10.3109/19401736.2015.1036248 (2016).
28 Hu, Z., Guan, D. L. & Mao, B. Y. Characterization of the complete mitochondrial genome of the Yunnan endemic grasshopper *Yunnanacris yunnaneus* (Insecta: Orthoptera: Acrididae). *Conserv Genet Resour* **8**, 267-270, doi:10.1007/s12686-016-0552-9 (2016).
29 Leavitt, J. R., Hiatt, K. D., Whiting, M. F. & Song, H. J. Searching for the optimal data partitioning strategy in mitochondrial phylogenomics: A phylogeny of Acridoidea (Insecta: Orthoptera: Caelifera) as a case study. *Molecular Phylogenetics and Evolution* **67**, 494-508, doi:10.1016/j.ympev.2013.02.019 (2013).
30 Li, R., Jiang, G. F., Liang, A. P., Zhong, X. T. & Liu, Y. Characterization of the mitochondrial genome of the montane grasshopper, *Qinlingacris elaeodes* (Orthoptera: Catantopidae). *Mitochondrial DNA A* **27**, 1765-1766, doi:10.3109/19401736.2014.963802 (2016).
31 Li, X. J., Zhi, Y. C., Lang, L. & Yin, X. C. The complete mitochondrial genome of *Filchnerella beicki* Ramme, 1931 (Orthoptera: Acridoidea: Pamphagidae). *Mitochondr DNA* **25**, 348-349, doi:10.3109/19401736.2013.803087 (2014).
32 Li, X. J., Zhi, Y. C., Liu, G. J., Yin, X. C. & Zhang, D. C. The complete mitochondrial genome of *Asiotmethis jubatus* (Uvarov, 1926) (Orthoptera: Acridoidea: Pamphagidae). *Mitochondr DNA* **26**, 785-786, doi:10.3109/19401736.2013.855752 (2015).
33 Liu, C. X., Chang, J., Ma, C., Li, L. & Zhou, S. Y. Mitochondrial genomes of two Sinochlora species (Orthoptera): novel genome rearrangements and recognition sequence of replication origin. *Bmc Genomics* **14**, doi:Artn 114
10.1186/1471-2164-14-114 (2013).
34 Liu, F. Complete mitochondrial genome of *Conocephalus melaenus* (Orthoptera: Tettigoniidae): a tRNA-like sequence on the J-strand. *Mitochondrial DNA B* **3**, 105-106, doi:10.1080/23802359.2018.1424582 (2018).
35 Liu, Y. & Huang, Y. Sequencing and analysis of complete mitochondrial genome of *Chorthippus chinensis* Tarb. *Chinese Journal of Biochemistry and Molecular Biology* **24**, 329-335 (2008).
36 Liu, N. A. & Huang, Y. A. Complete Mitochondrial Genome Sequence of *Acrida cinerea* (Acrididae: Orthoptera) and Comparative Analysis of Mitochondrial Genomes in Orthoptera. *Comp Funct Genom*, doi:Artn 319486
10.1155/2010/319486 (2010).
37 Ma, C., Liu, C. X., Yang, P. C. & Kang, L. The complete mitochondrial genomes of two band-winged grasshoppers, *Gastrimargus marmoratus* and *Oedaleus asiaticus*. *Bmc Genomics* **10**, doi:Artn 156
10.1186/1471-2164-10-156 (2009).
38 Marino-Perez, R. & Song, H. J. On the origin of the New World Pyrgomorphidae (Insecta: Orthoptera). *Molecular Phylogenetics and Evolution* **139**, doi:ARTN 106537
10.1016/j.ympev.2019.106537 (2019).
39 Sheffield, N. C., Hiatt, K. D., Valentine, M. C., Song, H. J. & Whiting, M. F. Mitochondrial genomics in Orthoptera using MOSAS. *Mitochondr DNA* **21**, 87-104, doi:10.3109/19401736.2010.500812 (2010).
40 Shi, H.-W., Ding, F.-M. & Huang, Y. Complete sequencing and analysis of mtDNA in *Phlaeoba albonema* Zheng. *Chinese Journal of Biochemistry and Molecular Biology* **24**, 604-611 (2008).
41 Shi, Q. Y., Zheng, J. Y., Zhi, Y. C. & Li, X. J. The complete mitochondrial genome of *Sinotmethis brachypterus* Zheng & Xi, 1985 (Orthoptera: Acridoidea: Pamphagidae: Prionotropisinae). *Mitochondrial DNA A* **27**, 4063-4064, doi:10.3109/19401736.2014.1003844 (2016).
42 Song, H. J. *et al.* 300 million years of diversification: elucidating the patterns of orthopteran evolution based on comprehensive taxon and gene sampling. *Cladistics* **31**, 621-651, doi:10.1111/cla.12116 (2015).
43 Song, H. J., Marino-Perez, R., Woller, D. A. & Cigliano, M. M. Evolution, Diversification, and Biogeography of Grasshoppers (Orthoptera: Acrididae). *Insect Syst Diver* **2**, doi:UNSP ixy008
10.1093/isd/ixy008 (2018).
44 Song, N., Li, H., Song, F. & Cai, W. Z. Molecular phylogeny of Polyneoptera (Insecta) inferred from expanded mitogenomic data. *Sci Rep-Uk* **6**, doi:ARTN 36175
10.1038/srep36175 (2016).

45 Song, W. W., Ye, B. H., Cao, X. W., Yin, H. & Zhang, D. C. The complete mitochondrial genome of *Phlaeoba tenebrosa* (Orthoptera: Acridoidea: Acrididae). *Mitochondrial DNA A* **27**, 409-410, doi:10.3109/19401736.2014.898281 (2016).
46 Song, W. W., Zhi, Y. C., Liu, G. J., Yin, H. & Zhang, D. C. The complete mitochondrial genome of *Orinhippus tibetanus* Uvarov, 1921 (Orthoptera: Acridoidea: Gomphoceridae). *Mitochondrial DNA A* **27**, 76-77, doi:10.3109/19401736.2013.873907 (2016).
47 Sun, H. M., Zheng, Z. M. & Huang, Y. A. Sequence and phylogenetic analysis of complete mitochondrial DNA genomes of two grasshopper species *Gomphocerus rufus* (Linnaeus, 1758) and *Primnoa arctica* (Zhang and Jin, 1985) (Orthoptera: Acridoidea). *Mitochondr DNA* **21**, 115-131, doi:10.3109/19401736.2010.482585 (2010).
48 Tang, M. *et al.* Multiplex sequencing of pooled mitochondrial genomes-a crucial step toward biodiversity analysis using mito-metagenomics. *Nucleic Acids Research* **42**, doi:ARTN e166 10.1093/nar/gku917 (2014).
49 Xiao, B., Chen, W., Hu, C. C. & Jiang, G. F. Complete mitochondrial genome of the groundhopper *Alulatettix yunnanensis* (Insecta: Orthoptera: Tetrigoidea). *Mitochondr DNA* **23**, 286-287, doi:10.3109/19401736.2012.674122 (2012).
50 Xiao, B., Feng, X., Miao, W. J. & Jiang, G. F. The complete mitochondrial genome of grouse locust *Tetrix japonica* (Insecta: Orthoptera: Tetrigoidea). *Mitochondr DNA* **23**, 288-289, doi:10.3109/19401736.2012.674123 (2012).
51 Xu, Q., Hao, Y., Mei, K. T., Yin, H. & Zhang, D. C. The complete mitochondrial genome of *Ceracris versicolor* (Orthoptera: Acridoidea: Arcypteridae). *Mitochondrial DNA A* **27**, 512-513, doi:10.3109/19401736.2014.905833 (2016).
52 Yang, H. & Huang, Y. Analysis of the complete mitochondrial genome sequence of *Pielomastax zhengi*. *Zoological Research* **32**, 353-362 (2011).
53 Yang, J., Liu, Y. & Liu, N. The complete mitochondrial genome of the *Xenocatantops brachycerus* (Orthoptera: Catantopidae). *Mitochondrial DNA A* **27**, 2844-2845, doi:10.3109/19401736.2015.1053121 (2016).
54 Yang, J., Ren, Q. L., Zhang, Q. & Huang, Y. Complete mitochondrial genomes of three crickets (Orthoptera: Gryllidae) and comparative analyses within Ensifera mitogenomes. *Zootaxa* **4092**, 529-547 (2016).
55 Yang, J., Ye, F. & Huang, Y. Mitochondrial genomes of four katydids (Orthoptera: Phaneropteridae): New gene rearrangements and their phylogenetic implications. *Gene* **575**, 702-711, doi:10.1016/j.gene.2015.09.052 (2016).
56 Yang, M. R., Zhou, Z. J., Chang, Y. L. & Zhao, L. H. The mitochondrial genome of the quiet-calling katydids, *Xizicus fascipes* (Orthoptera: Tettigoniidae: Meconematinae). *J Genet* **91**, 141-153, doi:10.1007/s12041-012-0157-3 (2012).
57 Yang, R., Guan, D. L. & Xu, S. Q. Complete mitochondrial genome of the Chinese endemic grasshopper *Fruhstorferiola kulinga* (Orthoptera: Acrididae: Podismini). *Mitochondrial DNA A* **27**, 3240-3241, doi:10.3109/19401736.2015.1007364 (2016).
58 Ye, W., Dang, J.-P., Xie, L.-D. & Huang, Y. Complete mitochondrial genome of *Teleogryllus emma* (Orthoptera: Gryllidae) with a new gene order in Orthoptera. *Zoological Research* **29**, 236-244 (2008).
59 Zhang, C. & Huang, Y. Complete mitochondrial genome of *Oxya chinensis* (Orthoptera, Acridoidea). *Acta Bioch Bioph Sin* **40**, 7-18, doi:10.1111/j.1745-7270.2008.00375.x (2008).
60 Zhang, D. C., Zhi, Y. C., Yin, H., Li, X. J. & Yin, X. C. The complete mitochondrial genome of *Thrinchus schrenkii* (Orthoptera: Caelifera, Acridoidea, Pamphagidae). *Mol Biol Rep* **38**, 611-619, doi:10.1007/s11033-010-0147-6 (2011).
61 Zhang, H. L., Zeng, H. H., Huang, Y. & Zheng, Z. M. The complete mitochondrial genomes of three grasshoppers, *Asiotmethis zacharjini*, *Filchnerella helanshanensis* and *Pseudotmethis rubimarginis* (Orthoptera: Pamphagidae). *Gene* **517**, 89-98, doi:10.1016/j.gene.2012.12.080 (2013).
62 Zhang, H. L., Zhao, L., Zheng, Z. M. & Huang, Y. Complete Mitochondrial Genome of *Gomphocerus sibiricus* (Orthoptera: Acrididae) and Comparative Analysis in Four Gomphocerinae Mitogenomes. *Zool Sci* **30**, 192-204, doi:10.2108/zsj.30.192 (2013).
63 Zhang, Q., Guo, C. A. & Huang, Y. The complete mitochondrial genome of *Gonista bicolor* (Haan) (Orthoptera: Acrididae). *Mitochondrial DNA A* **27**, 4578-4579, doi:10.3109/19401736.2015.1101572 (2016).
64 Zhang, X. M., Li, X. J., Liu, F., Yuan, H. & Huang, Y. A. The complete mitochondrial genome of *Tonkinacris sinensis* (Orthoptera: Acrididae): A tRNA-like sequence and its implications for phylogeny. *Biochem Syst Ecol* **70**, 147-154, doi:10.1016/j.bse.2016.11.002 (2017).
65 Zhang, Y. L., Liu, B., Zhang, H. Y., Yin, H. & Zhang, D. C. The complete mitochondrial genome of *Pacris xizangensis* (Orthoptera: Acridoidea: Gomphoceridae). *Mitochondrial DNA A* **27**, 320-321, doi:10.3109/19401736.2014.892097 (2016).

66 Zhang, Y. L., Shao, D. D., Cai, M., Yin, H. & Zhang, D. C. The complete mitochondrial genome of *Gryllotalpa unispina* Saussure, 1874 (Orthoptera: Gryllotalpoidea: Gryllotalpidae). *Mitochondrial DNA A* **27**, 159-160, doi:10.3109/19401736.2013.878923 (2016).
67 Zhao, L., Zheng, Z. M., Huang, Y. A. & Sun, H. M. A Comparative Analysis of Mitochondrial Genomes in Orthoptera (Arthropoda: Insecta) and Genome Descriptions of Three Grasshopper Species. *Zool Sci* **27**, 662-672, doi:10.2108/zsj.27.662 (2010).
68 Zhi, Y. C., Dong, L. J., Yin, H. & Zhang, D. C. The complete mitochondrial genome of *Mekongiella kingdoni* (Uvarov, 1937) (Orthoptera: Acridoidea: Chrotogonidae). *Mitochondrial DNA A* **27**, 187-188, doi:10.3109/19401736.2013.879653 (2016).
69 Zhi, Y. C., Lang, L., Ding, B. Y., Yin, H. & Zhang, D. C. The complete mitochondrial genome of one band-winged grasshopper, *Bryodema luctuosum luctuosum* Stoll (Orthoptera: Acridoidea). *Mitochondr DNA* **24**, 257-259, doi:10.3109/19401736.2012.760073 (2013).
70 Zhi, Y. C., Liu, B., Han, G. F., Yin, H. & Zhang, D. C. The complete mitochondrial genome of *Kingdonella bicollina* (Orthoptera: Acridoidea: Catantopidae). *Mitochondrial DNA A* **27**, 391-392, doi:10.3109/19401736.2014.896000 (2016).
71 Zhi, Y. C., Zhang, N., Lu, X. C., Yin, H. & Zhang, D. C. The complete mitochondrial genome of *Peripolus nepalensis* Uvarov, 1942 (Orthoptera: Acridoidea: Catantopidae). *Mitochondrial DNA A* **27**, 26-27, doi:10.3109/19401736.2013.869677 (2016).
72 Zhou, F. & Huang, Y. The complete mitochondrial genome of *Spathosternum prasiniferum sinense* Uvarov, 1931 (Orthoptera: Acridoidea: Acrididae). *Mitochondrial DNA A* **27**, 1932-1933, doi:10.3109/19401736.2014.971293 (2016).
73 Zhou, Z. J., Huang, Y., Shi, F. M. & Ye, H. Y. The complete mitochondrial genome of *Deracantha onos* (Orthoptera: Bradyporidae). *Mol Biol Rep* **36**, 7-12, doi:10.1007/s11033-007-9145-8 (2009).
74 Zhou, Z. J., Huang, Y. A. & Shi, F. M. The mitochondrial genome of *Ruspolia dubia* (Orthoptera: Conocephalidae) contains a short A+T-rich region of 70 bp in length. *Genome* **50**, 855-866, doi:10.1139/G07-057 (2007).
75 Zhou, Z., Shang, N., Huang, Y., Shi, F. & Wei, S. Sequencing and analysis of the mitochondrial genome of *Conocephalus maculatus* (Orthoptera: Conocephalinae). *Acta Entomologica Sinica* **54**, 548-554 (2011).
76 Zhou, Z. J., Shi, F. M. & Huang, Y. The complete mitogenome of the Chinese bush cricket, *Gampsocleis gratiosa* (Orthoptera: Tettigonioidea). *J Genet Genomics* **35**, 341-348, doi:Doi 10.1016/S1673-8527(08)60050-8 (2008).
77 Zhou, Z. J., Shi, F. M. & Zhao, L. The First Mitochondrial Genome for the Superfamily Hagloidea and Implications for Its Systematic Status in Ensifera. *Plos One* **9**, doi:ARTN e86027
10.1371/journal.pone.0086027 (2014).
78 Zhou, Z., Yang, M., Chang, Y. & Shi, F. Comparative analysis of mitochondrial genomes of two long-legged katydids (Orthoptera: Tettigoniidae). *Acta Entomologica Sinica* **56**, 408-418 (2013).
79 Zhou, J. X., Jia, Y. C., Yang, X. C. & Li, Q. The complete mitochondrial genome of the black field cricket, *Teleogryllus oceanicus*. *Mitochondrial DNA A* **28**, 229-230, doi:10.3109/19401736.2015.1115854 (2017).
80 Zhou, Z. J., Ye, H. Y., Huang, Y. A. & Shi, F. M. The phylogeny of Orthoptera inferred from mtDNA and description of *Elimaea cheni* (Tettigoniidae: Phaneropterinae) mitogenome. *J Genet Genomics* **37**, 315-324, doi:10.1016/S1673-8527(09)60049-7 (2010).
81 Zhou, Z. J. *et al.* Towards a higher-level Ensifera phylogeny inferred from mitogenome sequences. *Molecular Phylogenetics and Evolution* **108**, 22-33, doi:10.1016/j.ympev.2017.01.014 (2017).
82 Meng, G., Li, Y., Yang, C. & Liu, S. MitoZ: a toolkit for animal mitochondrial genome assembly, annotation and visualization. *Nucleic Acids Res* **47**, e63, doi:10.1093/nar/gkz173 (2019).
83 Krogh, A., Brown, M., Mian, I. S., Sjolander, K. & Haussler, D. Hidden Markov-Models in Computational Biology - Applications to Protein Modeling. *Journal of Molecular Biology* **235**, 1501-1531, doi:DOI 10.1006/jmbi.1994.1104 (1994).
84 Wheeler, T. J. & Eddy, S. R. nhmmer: DNA homology search with profile HMMs. *Bioinformatics* **29**, 2487-2489, doi:10.1093/bioinformatics/btt403 (2013).
85 Gertz, E. M., Yu, Y. K., Agarwala, R., Schaffer, A. A. & Altschul, S. F. Composition-based statistics and translated nucleotide searches: improving the TBLASTN module of BLAST. *BMC Biol* **4**, 41, doi:10.1186/1741-7007-4-41 (2006).
86 Birney, E., Clamp, M. & Durbin, R. GeneWise and Genomewise. *Genome Res* **14**, 988-995 (2004).
87 Durbin, R., Eddy, S. R., Krogh, A. & Mitchison, G. *Biological Sequence Analysis: Probabilistic Models of Proteins and Nucleic Acids*. (Cambridge University Press, 1998).

88 Juhling, F. *et al.* Improved systematic tRNA gene annotation allows new insights into the evolution of mitochondrial tRNA structures and into the mechanisms of mitochondrial genome rearrangements. *Nucleic Acids Research* **40**, 2833-2845, doi:10.1093/nar/gkr1131 (2012).
89 Nawrocki, E. P. & Eddy, S. R. Infernal 1.1: 100-fold faster RNA homology searches. *Bioinformatics* **29**, 2933-2935, doi:10.1093/bioinformatics/btt509 (2013).
90 Kriventseva, E. V., Rahman, N., Espinosa, O. & Zdobnov, E. M. OrthoDB: the hierarchical catalog of eukaryotic orthologs. *Nucleic Acids Res* **36**, D271-275, doi:10.1093/nar/gkm845 (2008).
91 Waterhouse, R. M., Tegenfeldt, F., Li, J., Zdobnov, E. M. & Kriventseva, E. V. OrthoDB: a hierarchical catalog of animal, fungal and bacterial orthologs. *Nucleic Acids Res* **41**, D358-365, doi:10.1093/nar/gks1116 (2013).
92 Waterhouse, R. M., Zdobnov, E. M., Tegenfeldt, F., Li, J. & Kriventseva, E. V. OrthoDB: the hierarchical catalog of eukaryotic orthologs in 2011. *Nucleic Acids Res* **39**, D283-288, doi:10.1093/nar/gkq930 (2011).
93 Petersen, M. *et al.* Orthograph: a versatile tool for mapping coding nucleotide sequences to clusters of orthologous genes. *BMC Bioinformatics* **18**, 111, doi:10.1186/s12859-017-1529-8 (2017).
94 Kirkness, E. F. *et al.* Genome sequences of the human body louse and its primary endosymbiont provide insights into the permanent parasitic lifestyle. *Proc Natl Acad Sci U S A* **107**, 12168-12173, doi:10.1073/pnas.1003379107 (2010).
95 Mesquita, R. D. *et al.* Genome of Rhodnius prolixus, an insect vector of Chagas disease, reveals unique adaptations to hematophagy and parasite infection. *Proc Natl Acad Sci U S A* **112**, 14936-14941, doi:10.1073/pnas.1506226112 (2015).
96 Terrapon, N. *et al.* Molecular traces of alternative social organization in a termite genome. *Nature communications* **5**, 3636, doi:10.1038/ncomms4636 (2014).
97 Werren, J. H. *et al.* Functional and evolutionary insights from the genomes of three parasitoid Nasonia species. *Science* **327**, 343-348, doi:10.1126/science.1178028 (2010).
98 Slater, G. S. & Birney, E. Automated generation of heuristics for biological sequence comparison. *BMC Bioinformatics* **6**, 31, doi:10.1186/1471-2105-6-31 (2005).
99 Katoh, K. & Standley, D. M. MAFFT multiple sequence alignment software version 7: improvements in performance and usability. *Mol Biol Evol* **30**, 772-780, doi:10.1093/molbev/mst010 (2013).
100 Suyama, M., Torrents, D. & Bork, P. PAL2NAL: robust conversion of protein sequence alignments into the corresponding codon alignments. *Nucleic Acids Res* **34**, W609-612, doi:10.1093/nar/gkl315 (2006).
101 Punta, M. *et al.* The Pfam protein families database. *Nucleic Acids Res* **40**, D290-301, doi:10.1093/nar/gkr1065 (2012).
102 Eddy, S. R. Accelerated Profile HMM Searches. *PLoS Comput Biol* **7**, e1002195, doi:10.1371/journal.pcbi.1002195 (2011).
103 Misof, B. & Misof, K. A Monte Carlo Approach Successfully Identifies Randomness in Multiple Sequence Alignments : A More Objective Means of Data Exclusion. *Systematic Biology* **58**, 21-34 (2009).
104 Kück, P. *et al.* Parametric and non-parametric masking of randomness in sequence alignments can be improved and leads to better resolved trees. *Front Zool* **7**, 10 (2010).
105 ALICUT V2.0, http://www.utilities.zfmk.de (2009).
106 Misof, B. *et al.* Selecting informative subsets of sparse supermatrices increases the chance to find correct trees. *BMC Bioinformatics* **14**, 348, doi:10.1186/1471-2105-14-348 (2013).
107 Wong, T. K. F. *et al.* A minimum reporting standard for multiple sequence alignments. *NAR Genomics and Bioinformatics* **2**, doi:10.1093/nargab/lqaa024 (2020).
108 Lanfear, R., Frandsen, P. B., Wright, A. M., Senfeld, T. & Calcott, B. PartitionFinder 2: New Methods for Selecting Partitioned Models of Evolution for Molecular and Morphological Phylogenetic Analyses. *Mol Biol Evol* **34**, 772-773, doi:10.1093/molbev/msw260 (2017).
109 Pauli, T. *et al.* Phylogenetic analysis of cuckoo wasps (Hymenoptera: Chrysididae) reveals a partially artificial classification at the genus level and a species-rich clade of bee parasitoids. *Systematic Entomology* **44**, 322-335, doi:10.1111/syen.12323 (2019).
110 Gu, X., Fu, Y. X. & Li, W. H. Maximum likelihood estimation of the heterogeneity of substitution rate among nucleotide sites. *Mol Biol Evol* **12**, 546-557 (1995).
111 Kosiol, C. & Goldman, N. Different versions of the Dayhoff rate matrix. *Mol Biol Evol* **22**, 193-199, doi:10.1093/molbev/msi005 (2005).
112 Le, S. Q. & Gascuel, O. An improved general amino acid replacement matrix. *Mol Biol Evol* **25**, 1307-1320, doi:10.1093/molbev/msn067 (2008).
113 Muller, T. & Vingron, M. Modeling amino acid replacement. *J Comput Biol* **7**, 761-776, doi:10.1089/10665270050514918 (2000).
114 Soubrier, J. *et al.* The influence of rate heterogeneity among sites on the time dependence of molecular rates. *Mol Biol Evol* **29**, 3345-3358, doi:10.1093/molbev/mss140 (2012).

115 Veerassamy, S., Smith, A. & Tillier, E. R. A transition probability model for amino acid substitutions from blocks. *J Comput Biol* **10**, 997-1010, doi:10.1089/106652703322756195 (2003).
116 Whelan, S. & Goldman, N. A general empirical model of protein evolution derived from multiple protein families using a maximum-likelihood approach. *Mol Biol Evol* **18**, 691-699 (2001).
117 Yang, Z. Maximum likelihood phylogenetic estimation from DNA sequences with variable rates over sites: approximate methods. *J Mol Evol* **39**, 306-314 (1994).
118 Kalyaanamoorthy, S., Minh, B. Q., Wong, T. K. F., von Haeseler, A. & Jermiin, L. S. ModelFinder: fast model selection for accurate phylogenetic estimates. *Nat Methods* **14**, 587-589, doi:10.1038/nmeth.4285 (2017).
119 Nguyen, L. T., Schmidt, H. A., von Haeseler, A. & Minh, B. Q. IQ-TREE: a fast and effective stochastic algorithm for estimating maximum-likelihood phylogenies. *Mol Biol Evol* **32**, 268-274, doi:10.1093/molbev/msu300 (2015).
120 Edgar, R. C. MUSCLE: a multiple sequence alignment method with reduced time and space complexity. *BMC Bioinformatics* **5**, 113 (2004).
121 Vaidya, G., Lohman, D. J. & Meier, R. SequenceMatrix: concatenation software for the fast assembly of multi-gene datasets with character set and codon information. *Cladistics* **27**, 171-180, doi:10.1111/j.1096-0031.2010.00329.x (2011).
122 Lanfear, R., Calcott, B., Ho, S. Y. & Guindon, S. Partitionfinder: combined selection of partitioning schemes and substitution models for phylogenetic analyses. *Mol Biol Evol* **29**, 1695-1701, doi:10.1093/molbev/mss020 (2012).
123 Chernomor, O., von Haeseler, A. & Minh, B. Q. Terrace Aware Data Structure for Phylogenomic Inference from Supermatrices. *Syst Biol* **65**, 997-1008, doi:10.1093/sysbio/syw037 (2016).
124 Strimmer, K. & von Haeseler, A. Likelihood-mapping: a simple method to visualize phylogenetic content of a sequence alignment. *Proc Natl Acad Sci U S A* **94**, 6815-6819 (1997).
125 Simon, S., Blanke, A. & Meusemann, K. Reanalyzing the Palaeoptera problem - The origin of insect flight remains obscure. *Arthropod Struct Dev* **47**, 328-338, doi:10.1016/j.asd.2018.05.002 (2018).
126 Ho, S. Y. & Jermiin, L. Tracing the decay of the historical signal in biological sequence data. *Syst Biol* **53**, 623-637, doi:10.1080/10635150490503035 (2004).
127 Jermiin, L. S., Ho, S. Y. W., Ababneh, F., Robinson, J. & Larkum, A. W. D. The biasing effect of compositional heterogeneity on phylogenetic estimates may be underestimated. *Syst Biol* **53**, 638-643 (2004).
128 Mark, D. F., Rice, C. M. & Trewin, N. H. Discussion on 'A high-precision U–Pb age constraint on the Rhynie Chert Konservat-Lagerstätte: time scale and other implications'. *Journal, Vol. 168, 863–872* **170**, 701-703, doi:10.1144/jgs2011-110 (2013).
129 Evangelista, D. A. *et al.* An integrative phylogenomic approach illuminates the evolutionary history of cockroaches and termites (Blattodea). *Proc Biol Sci* **286**, 20182076, doi:10.1098/rspb.2018.2076 (2019).
130 Yang, Z. & Rannala, B. Bayesian estimation of species divergence times under a molecular clock using multiple fossil calibrations with soft bounds. *Mol Biol Evol* **23**, 212-226, doi:10.1093/molbev/msj024 (2006).
131 Rambaut, A., Drummond, A. J., Xie, D., Baele, G. & Suchard, M. A. Posterior Summarization in Bayesian Phylogenetics Using Tracer 1.7. *Syst Biol* **67**, 901-904, doi:10.1093/sysbio/syy032 (2018).
132 Gu, J.-J., Béthoux, O. & Ren, D. A new, rare and distinctive species of Panorthoptera (Insecta, Archaeorthoptera) from the Upper Carboniferous of Xiaheyan (Ningxia, China). *Fossil Record* **20**, 253-257 (2017).
133 Kukalová-Peck, J. & Brauckmann, C. Paleozoic Protorthoptera are ancestral hemipteroids: major wing braces as clues to a new phylogeny of Neoptera (Insecta). *Canadian Journal of Zoology/Revue Canadienne de Zoologie* **70**, 2452-2473 (1992).
134 Sharov, A. G. Filogeniya orthopteroidnykh nasekomykh. *Trudy Paleontologicheskogo instituta, Akademiya Nauk SSSR* **118**, 1–216 (1968).
135 Sharov, A. G. *Phylogeny of the Orthopteroidea*. (Israel Program for Scientific Translations, 1971).
136 Béthoux, O. *Grylloptera* – a unique origin of the stridulatory file in katydids, crickets, and their kin (*Archaeorthoptera*). *Arth. Syst. Phyl.* **70**, 43–68 (2012).
137 Hebard, M. A revision of the North American species of the genus *Myrmecophila* (Orthoptera; Gryllidae; Myrmecophilinae). *Transactions of the American Entomological Society* **46**, 91-111 (1920).
138 Ward, P. S. The phylogeny and evolution of ants. *Annual Review of Ecology, Evolution and Systematics* **45**, 23-43 (2014).
139 Ramme, W. Systematisches, Verbereitung und Morpho-Biologisches aus der Gryllacriden-Unterfamilie Schizodactylinae (Orth). *Zeitschrift für Morphologie und Őkologie der Tiere* **22**, 163-172 (1931).
140 Béthoux, O. King crickets, raspy crickets & weta, their wings, their fossil relatives. *J. Orthopt. Res.* **21**, 179–225, doi:10.1665/034.021.0206 (2012).

141 Song, H. *et al.* 300 million years of diversification: elucidating the patterns of orthopteran evolution based on comprehensive taxon and gene sampling. *Cladistics* **31**, 621–651, doi:10.1111/cla.12116 (2015).
142 Vandergast, A. G. *et al.* Tackling an intractable problem: Can greater taxon sampling help resolve relationships within the Stenopelmatoidea (Orthoptera: Ensifera)? *Zootaxa* **4291**, 1–33, doi:10.11646/zootaxa.4291.1.1 (2017).
143 Desutter-Grandcolas, L. Phylogeny and the evolution of acoustic communication in extant Ensifera (Insecta, Orthoptera). *Zoologica Scripta* **32**, 525–561 (2003).
144 Gwynne, D. T. Phylogeny of the Ensifera (Orthoptera): a hypothesis supporting multiple origins of acoustical signalling, complex spermatophores and maternal care in crickets, katydids, and weta. *Journal of Orthoptera Research* **4**, 203-218 (1995).
145 Zeuner, F. E. *Fossil Orthoptera Ensifera*. (British Museum (Natural History), 1939).
146 Heads, S. W., Thomas, M. J. & Wang, Y. A remarkable new pygmy grasshopper (Orthoptera, Tetrigidae) in Miocene amber from the Dominican Republic. *Zookeys* **429**, 87-100 (2014).
147 Dirsh, V. M. *Classification of the Acridomorphoid Insects*. (E.W. Classey Ltd., 1975).
148 Parham, J. F. *et al.* Best practices for justifying fossil calibrations. *Syst Biol* **61**, 346–359, doi:10.1093/sysbio/syr107 (2012).
149 Béthoux, O. Groundplan, nomenclature, homology, phylogeny, and the question of the insect wing venation pattern. *Alavesia* **2**, 219–232 (2008).
150 Ragge, D. R. *The wing-venation of the Orthoptera Saltatoria with notes on dictyopteran wing-venation*. (British Museum (Natural History), 1955).
151 Gorochov, A. V. System and evolution of the suborder Ensifera (Orthoptera). Part I. *Proceedings of the Zoological Institute, Russian Academy of Sciences* **260**, 1–224 (1995).
152 Béthoux, O. & Nel, A. Venation pattern of Orthoptera. *Journal of Orthoptera Research* **10**, 195–198, doi:10.1665/1082-6467(2001)010[0195:VPOO]2.0.CO;2 (2001).
153 Béthoux, O. & Nel, A. Venation pattern and revision of Orthoptera sensu nov. and sister groups. Phylogeny of Palaeozoic and Mesozoic Orthoptera sensu nov. *Zootaxa* **96**, 1–88, doi:10.11646/zootaxa.96.1.1 (2002).
154 Gorochov, A. V. System and evolution of the suborder Ensifera (Orthoptera). Part II. *Proceedings of the Zoological Institute, Russian Academy of Sciences* **260**, 1–207 (1995).
155 Kluge, N. G. Structure of ovipositors and Cladoendesis of Saltatoria, or Orchesopia. *Entomological Review* **96**, 1015–1040 (2016).
156 Ander, K. Vergleichend anatomische und phylogenetische Studien über die Ensifera (Saltatoria). *Opuscula Entomologica Supplementum* **2**, 1–306 (1939).
157 Desutter-Grandcolas, L. *et al.* 3-D imaging reveals four extraordinary cases of convergent evolution of acoustic communication in crickets and allies (Insecta). *Sci Rep-Uk* **7**, 1–8, doi:10.1038/s41598-017-06840-6 (2017).
158 Chivers, B. D. *et al.* Functional morphology of tegmina-based stridulation in the relict species *Cyphoderris monstrosa* (Orthoptera: Ensifera: Prophalangopsidae). *Journal of Experimental Biology* **220**, 1112–1121, doi:10.1242/jeb.153106 (2017).
159 Ansorge, J. Insekten aus dem oberen Lias von Grimmen (Vorpommern, Norddeutschland). *Neues Paläontologie Abhandlungen* **2**, 1–132 (1996).
160 Jarzembowski, E. A. in *Grasshoppers and crickets [cockroaches and earwigs] of Surrey* (ed D. W. Baldock) 20–22, pls. 21–22 (Surrey Wildlife Trust, 1999).
161 Gorochov, A. V., Jarzembowski, E. A. & Coram, R. A. Grasshoppers and crickets (Insecta: Orthoptera) from the Lower Cretaceous of southern England. *Cret. Res.* **27**, 641–662, doi:10.1016/j.cretres.2006.03.007 (2006).
162 Gorochov, A. V. The most interesting finds of orthopteroid insects at the en of the 20th century and a new recent genus and species. *Journal of Orthoptera Research* **10**, 353-367 (2001).
163 Heads, S. & Martins-Neto, R. G. in *The Crato fossil beds of Brazil* (eds D. M. Martill, G. Bechly, & R. F. Loveridge) 265–283 (Cambridge University Press, 2007).
164 Martins-Neto, R. G. Complementos ao estudo sobre os Ensifera (Insecta, Orthopteroida) da Formação Santana, Cretáceo inferior do Nordeste do Brasil. *Revista brasileira de Entomologia* **39**, 321–345 (1995).
165 Martins-Neto, R. G. Um novo gênero e duas nov expécies de Tridactylidae (Insecta, Orthopteridea na Formação Santana (Cretáceo Inferior do Nordeste do Brasil). *Anais da Academia Brasileira de Ciências* **62**, 51–59 (1990).
166 Rentz, D. C. F. in *The Insects of Australia* Vol. 1 (eds I. D. Naumann *et al.*) 369–393 (Melbourne University Press, 1991).
167 Günther, K. K. Einige Bemerkungen über die Gattungen der Familie Tridactylidae Brunner und zur Klassification der Tridactylodea. *Deutsche Entomologische Zeitschrift* **26**, 255–264, doi:10.1002/mmnd.19790260408 (1979).

168 Scudder, S. H. *The Tertiary insects of North America*. (Government Printing Office, 1890).
169 Wolfe, J. A., Daley, A. C., Legg, D. A. & Edgecombe, G. D. Fossil calibrations for the arthropod Tree of Life. *Earth-Science Reviews* **160**, 43–110, doi:10.1016/j.earscirev.2016.06.008 (2016).
170 Gorochov, A. V. Novye iskopaemye priamokrylye semieïstv Adumbratomorphidae fam. n., Pruvostitidae i Proparagryllacrididae (Orthoptera, Ensifera) iz permskikh i triassovykh otlojeniï SSSR. *Vestnik Zoologii* **4**, 20–28 (1987).
171 Riek, E. F. A re-examination of the Mecopteroid and Orthopteroid fossils (Insecta) from the Triassic beds at Denmark Hill, Queensland, with description of further specimens. *Australian Journal of Zoology* **4**, 98–110 (1956).
172 Gorochov, A. V. Triassovie priamokrilie nadsimeistva Hagloidea (Orthoptera) [Triassic Orthoptera of the superfamily Hagloidea (Orthoptera)]. *USSR Academy of Sciences, Proceedings of the Zoological Institute, Leningrad* **143**, 65–100 (1986).
173 Heads, S. & Leuzinger, L. On the placement of the Cretaceous orthopteran *Brauckmannia groeningae* from Brazil, with notes on the relationships of Schizodactylidae (Orthoptera, Ensifera). *Zookeys* **77**, 17–30 (2011).
174 Pérez de la Fuente, R., Heads, S. W. & Hinojosa-Díaz, I. A. The first record of Protogryllinae from the Jurassic of India (Orthoptera: Protogryllidae). *Journal of the Kansas Entomological Society* **85**, 53–58 (2012).
175 Haughton, S. H. The fauna and stratigraphy of the Stormberg Series. *Annals of the South African Museum* **12**, 323–497 (1924).
176 Trümper, S. *et al.* Age and depositional environment of the Xiaheyan insect fauna, embedded in marine black shales (Early Pennsylvanian, China). *Palaeogeography, Palaeoclimatology, Palaeoecology*, doi:10.1016/j.palaeo.2019.109444 (in press).
177 Cohen, K. M., Finney, S. C., Gibbard, P. L. & Fan, J.-X. The ICS International Chronostratigraphic Chart (updated v2017/02). *Episodes* **36**, 199–204 (2013).
178 Radley, J. D. & Allen, P. The Wealden (non-marine Lower Cretaceous) of the Weald Sub-basin, southern England. *Proceedings of the Geologists' Association* **123**, 245–318, doi:10.1016/j.pgeola.2012.01.003 (2012).
179 Rasnitsyn, A. P., Jarzembowski, E. A. & Ross, A. Wasps (Insecta: Vespida = Hymenoptera) from the Purbeck and Wealden (Lower Cretaceous) of southern England and their biostratigraphical and palaeoenvironmental significance. *Cretaceous Research* **19**, 329–391, doi:10.1006/cres.1997.0114 (1998).
180 Prothero, D. R. & Sanchez, F. Magnetic stratigraphy of the upper Eocene Florissant Formation, Teller County, Colorado. *New Mexico Museum of Natural History and Science Bulletin* **26**, 143–147 (2004).
181 Revell, L. J. phytools: an R package for phylogenetic comparative biology (and other things). *Methods Ecol Evol* **3**, 217-223, doi:10.1111/j.2041-210X.2011.00169.x (2012).
182 van Staaden, M. J. & Romer, H. Evolutionary transition from stretch to hearing organs in ancient grasshoppers. *Nature* **394**, 773-776, doi:Doi 10.1038/29517 (1998).
183 Houston, T. F. Observations of the biology and immature stages of the sandgroper *Cylindraustralia kochii* (Saussure), with notes on some congeners (Orthoptera: Cylindrachetidae). *Records of the Western Australian Museum* **23**, 219-234 (2007).
184 Dirsh, V. M. Tanaoceridae and Xyronotidae: Two new families of Acridoidea (Orthoptera). *The Annals and Magazine of Natural History* **8**, 285-288 (1955).
185 Massa, B. The role of the Krauss's organ in sound production in Pamphagidae (Caelifera: Orthoptera). *Ital J Zool* **79**, 441-449, doi:10.1080/11250003.2012.667158 (2012).
186 Lopez, H., Garcia, M. D., Clemente, E., Presa, J. J. & Oromi, P. Sound production mechanism in pamphagid grasshoppers (Orthoptera). *J Zool* **275**, 1-8, doi:10.1111/j.1469-7998.2007.00394.x (2008).
187 Rabosky, D. L. *et al.* BAMMtools: an R package for the analysis of evolutionary dynamics on phylogenetic trees. *Methods Ecol Evol* **5**, 701-707, doi:10.1111/2041-210x.12199 (2014).
188 Cigliano, M. M., Braun, H., Eades, D. C. & Otte, D. (<http://Orthoptera.SpeciesFile.org>, 2019).
189 Beaulieu, J. M. & O'Meara, B. C. Detecting hidden diversification shifts in models of trait-dependent speciation and extinction. *Syst. Biol.* **65**, 583-601 (2016).
190 Maddison, W. P., Midford, P. E. & Otto, S. P. Estimating a binary character's effect on speciation and extinction. *Syst. Biol.* **56**, 701-710, doi:10.1080/10635150701607033 (2007).
