# Supplementary material for: Phylogenomic analysis sheds light on the evolutionary pathways towards acoustic communication in Orthoptera
Source: Nat Commun. 2020 Oct 2;11:4939. doi: 10.1038/s41467-020-18739-4 (PMC7532154; doi:10.1038/s41467-020-18739-4)
Supplement: Supplementary file 3 — Reporting Summary [file 41467_2020_18739_MOESM3_ESM.pdf]

nature research

Corresponding author(s): Hojun Song

Last updated by author(s): Aug 28, 2020

Reporting Summary

Nature Research wishes to improve the reproducibility of the work that we publish. This form provides structure for consistency and transparency in reporting. For further information on Nature Research policies, see Authors & Referees and the Editorial Policy Checklist .

Statistics

For all statistical analyses, confirm that the following items are present in the figure legend, table legend, main text, or Methods section.

n/a	Confirmed	
☒	☐	The exact sample size (*n*) for each experimental group/condition, given as a discrete number and unit of measurement
☒	☐	A statement on whether measurements were taken from distinct samples or whether the same sample was measured repeatedly
☒	☐	The statistical test(s) used AND whether they are one- or two-sided<br>*Only common tests should be described solely by name; describe more complex techniques in the Methods section.*
☒	☐	A description of all covariates tested
☒	☐	A description of any assumptions or corrections, such as tests of normality and adjustment for multiple comparisons
☒	☐	A full description of the statistical parameters including central tendency (e.g. means) or other basic estimates (e.g. regression coefficient) AND variation (e.g. standard deviation) or associated estimates of uncertainty (e.g. confidence intervals)
☒	☐	For null hypothesis testing, the test statistic (e.g. *F*, *t*, *r*) with confidence intervals, effect sizes, degrees of freedom and *P* value noted<br>*Give P values as exact values whenever suitable.*
☐	☒	For Bayesian analysis, information on the choice of priors and Markov chain Monte Carlo settings
☒	☐	For hierarchical and complex designs, identification of the appropriate level for tests and full reporting of outcomes
☒	☐	Estimates of effect sizes (e.g. Cohen's *d*, Pearson's *r*), indicating how they were calculated

Our web collection on statistics for biologists contains articles on many of the points above.

Software and code

Policy information about availability of computer code

Data collection	MitoZ , tBlastn v2.2.19, Genewise v2.2.0, MiTFi and Geneious 10 for mitochondrial genome assembly and annotation; SOAPdenovo-Trans v1.01 and Trinity v2.2.0 for transcriptome assembly; VecScreen and UniVec database build 7.1 for checking contamination; Orthograph v0.5.3 for for orthology prediction; MAFFT v7.130b and MUSCLE v3.8.425 for multiple sequence alignment; PAL2NAL v14 for making corresponding nucleotide sequences for amino acid alignment; Aliscore v1.2 to identify blocks of putative alignment ambiguities; AliCUT v2.3 to remove putative alignment ambiguities; MARE v0.1.2-rc to assess the information content of each data block; SymTest v.2.0.47 to test for data homogeneity; AliStat v1.6 to evaluate the coverage of all transcriptome-based datasets; PartitionFinder 2.0.0 (prerelease 17) for finding best-fit partitioning schemes; IQ-TREE v1.5.4 to select the best substitution model for each of the metapartitions
Data analysis	IQ-TREE v1.5.4 for maximum likelihood analysis and Four-cluster Likelihood Mapping (FcLM); PAML v4.9 for divergence time estimate analysis using MCMCTree; phytools v0.6.99 for ancestral character state reconstruction and Pagel's (1994) binary character correlation test in R version 3.6.1; BAMM v2.5.0 and the R package BAMMtools v2.1.7 for diversification analysis; hisse v.1.9.6 for trait-dependent diversification analysis in R version 3.6.1

For manuscripts utilizing custom algorithms or software that are central to the research but not yet described in published literature, software must be made available to editors/reviewers. We strongly encourage code deposition in a community repository (e.g. GitHub). See the Nature Research guidelines for submitting code & software for further information.

Data

Policy information about availability of data

All manuscripts must include a data availability statement. This statement should provide the following information, where applicable:

- Accession codes, unique identifiers, or web links for publicly available datasets
- A list of figures that have associated raw data
- A description of any restrictions on data availability

Data availability statement has been included in the manuscript. Supplementary Information is available for this paper. All data used for this study have been

uploaded to the Dryad Digital Repository (https://doi.org/10.5061/dryad.qjq2bvqc6). All sequencing data are available at NCBI via the accession numbers provided in Supplementary Data 1 and 3.

Field-specific reporting

Please select the one below that is the best fit for your research. If you are not sure, read the appropriate sections before making your selection.

☐ Life sciences ☐ Behavioural & social sciences ☒ Ecological, evolutionary & environmental sciences

For a reference copy of the document with all sections, see nature.com/documents/nr-reporting-summary-flat.pdf

Ecological, evolutionary & environmental sciences study design

All studies must disclose on these points even when the disclosure is negative.

Study description	This study is a molecular phylogenetic analysis based on transcriptome and mitochondrial genome data generated from RNA-grade and DNA-grade specimens. The taxonomic focus of this study is the insect order Orthoptera, which includes familiar insects such as grasshoppers, katydids, and crickets. This study is an output from the 1000 Insect Transcriptome Evolution (1KITE) consortium.
Research sample	The samples used for this study are 249 insect specimens (10 polyneopteran outgroups and 239 orthopteran ingroups) designed to study the phylogenetic relationships within the order Orthoptera. The samples were selected based on the phylogenetic diversity to represent all major lineages (all 16 superfamilies and 36 families of extant Orthoptera). We included 60 transcriptomes, of which 39 orthopteran species were newly generated either by the 1K Insect Transcriptome Evolution (1KITE) consortium or by the Song Lab at Texas A&M University. The remaining 21 transcriptomes (11 orthopteran and 10 polyneopteran) were from the previous publications (see Supplementary Methods 1.1). To increase taxon sampling, we then combined the transcriptome data with 169 previously and 80 newly generated mtgenomes from 249 taxa. The taxon sampling information with proper accession numbers is presented in Supplementary Data 1 and 3.
Sampling strategy	The samples were selected based on the phylogenetic diversity to represent all major lineages, as well as the availability of RNA-grade and DNA-grade tissue samples. Our sampling strategy to combine (i) backbone taxa with both transcriptome and mitochondrial genome data, and (ii) additional taxa with mitochondrial genome data, has not been attempted in phylogenomic studies within insects. A conceptually similar approach of combining data-rich backbone taxa and additional taxa with less data had been applied previously in Song et al. 2015 [Song, H. et al. 300 million years of diversification: elucidating the patterns of orthopteran evolution based on comprehensive taxon and gene sampling. Cladistics 31, 621–651 (2015).]
Data collection	RNA extraction, cDNA library preparation, and transcriptome sequencing and assembly were performed within the 1KITE project using the protocols detailed in Supplementary Methods 1.2 and 1.5. Protocols used for the Song Lab samples, as well as for mtgenome data generation are detailed in Supplementary Methods 1.3 and 1.4. H.S., A.D., H.L., and B.W. collected or provided samples. H.S., A.D., H.L., S.L., L.P., X.Z., and S.Si. assembled and processed the transcriptomes. H.S., S.L., and G.M. assembled and processed the mitochondrial genomes. S.Sh. performed orthology search. S.Si. performed Four-cluster Likelihood Mapping (FcLM) and permutation analyses. O.B. compiled fossil calibration choices.
Timing and spatial scale	The sequence data were collected between 2016 and 2018. This time frame coincides with the active data generation period of the 1KITE project and the project ongoing in the Song Lab.
Data exclusions	We removed potential sequence contaminations before transcriptome/mitochondrial genome assemblies. Once the phylogenetic matrix was formulated, no data were excluded.
Reproducibility	We have provided all data used in the study in Dryad, and provided detailed methods in the Supplementary Information and we believe that the study is highly reproducible.
Randomization	This is a phylogenetic analysis, which is based on taxon sampling and character sampling, and applying specific models of nucleotide substitution to infer likelihood. The methods used in phylogenetics is fundamentally different from a standard experimental design that requires control and treatment groups with a randomized design.
Blinding	The type of questions and analyses pursued in this study does not require blinding experiment design, because there is no participants who may be influenced by the treatments. Blinding is not applicable to phylogenetic analyses.

Did the study involve field work? ☐ Yes ☒ No

Reporting for specific materials, systems and methods

We require information from authors about some types of materials, experimental systems and methods used in many studies. Here, indicate whether each material, system or method listed is relevant to your study. If you are not sure if a list item applies to your research, read the appropriate section before selecting a response.

Materials & experimental systems

n/a	Involved in the study
☒	☐ Antibodies
☒	☐ Eukaryotic cell lines
☒	☐ Palaeontology
☐	☒ Animals and other organisms
☒	☐ Human research participants
☒	☐ Clinical data

Methods

n/a	Involved in the study
☒	☐ ChIP-seq
☒	☐ Flow cytometry
☒	☐ MRI-based neuroimaging

Animals and other organisms

Policy information about studies involving animals; ARRIVE guidelines recommended for reporting animal research

Laboratory animals	The study did not involve laboratory animals.
Wild animals	The insects that were used for generating sequence data were originally collected in the wild. They were collected, killed, and directly preserved in RNA-later or 100% ethanol to preserve RNA and DNA, respectively. This study did not involve any live animals.
Field-collected samples	The sequence data used in this study largely came from field-collected insect specimens. The insects were collected directly in RNA-later or 100% ethanol.
Ethics oversight	There was no ethical approval or guidance required for this study because the type of study organisms were common insects.

Note that full information on the approval of the study protocol must also be provided in the manuscript.
