# Supplementary material for: Phylogenomic analysis sheds light on the evolutionary pathways towards acoustic communication in Orthoptera
Source: Nat Commun. 2020 Oct 2;11:4939. doi: 10.1038/s41467-020-18739-4 (PMC7532154; doi:10.1038/s41467-020-18739-4)
Supplement: Supplementary file 4 — Description of Additional Supplementary Files [file 41467_2020_18739_MOESM4_ESM.pdf]

Description of Additional Supplementary Files

Supplementary Data 1

Detailed transcriptome species list including NCBI accession numbers.

Supplementary Data 2

Cross-contamination and assembly statistics. Information on sequences removed during various contamination filtering steps. *A negative number indicates an increase by the number of sequences. This can happen if a sequence is split in two parts by removing the contaminant.

Supplementary Data 3

Detailed mitochondrial genome species list including NCBI accession numbers.

Supplementary Data 4

Orthograph statistics. Number of orthologous sequence clusters used for each species for which transcripts were identified; non-overlapping transcripts identified for one sequence cluster were concatenated.

Supplementary Data 5

Detailed information of the generated datasets.

Supplementary Data 6

Selected groups for testing position of Rhaphidophoridae within Ensifera for the four transcriptome only datasets.

Supplementary Data 7

Selected groups for testing position of Rhaphidophoridae within Ensifera for the two combined datasets (transcriptome+mitochondrial).

Supplementary Data 8

Selected groups for testing position of Gryllotalpidae within Gryllidea for the four transcriptome only datasets.

Supplementary Data 9

Selected groups for testing position of Gryllotalpidae within Gryllidea for the two combined datasets (transcriptome+mitochondrial).

Supplementary Data 10

Selected groups for testing position of Pamphagidae within Caelifera for the four transcriptome only datasets.

Supplementary Data 11

Selected groups for testing position of Pamphagidae within Caelifera for the two combined datasets (transcriptome+mitochondrial).

Supplementary Data 12

Selected fossil calibrations used in our study. Information on used fossils are derived from Evangelista et al. (2019) or elaborated in Supplementary material S2.

Supplementary Data 13

Results of sensitivity analysis. Divergence time estimates for 11 selected nodes and excluding different calibration points.

Supplementary Data 14

Complete list of characters used for ancestral state reconstruction.

Supplementary Data 15

Presence-absence matrix for both hearing and sound production within Orthoptera.
