# Supplementary material for: Phylogenomic analysis sheds light on the evolutionary pathways towards acoustic communication in Orthoptera
Source: Nat Commun. 2020 Oct 2;11:4939. doi: 10.1038/s41467-020-18739-4 (PMC7532154; doi:10.1038/s41467-020-18739-4)
Supplement: Supplementary file 2 — Peer Review [file 41467_2020_18739_MOESM2_ESM.pdf]

Reviewers' comments:

Reviewer #1 (Remarks to the Author):

"Phylogenomic analysis of Orthoptera sheds new light on the evolution of acoustic communication"
by Song et al., submitted to NATURE COMMUNICATIONS

General:
Orthoptera provide important model species for the study of animal behaviour and communication, sexual selection, speciation, and physiology. While it is hard to overestimate their importance for biological research, evolutionary analyses are also hampered by the lack of a robust phylogeny at the higher taxon level. The morphological and molecular phylogenies published over the last 20 years resulted in contradicting relationships, or lacked rigorous distinction of coding between the species level and higher taxonomic levels. The manuscript submitted by Song et al. addresses this phylogenetic problem with a phylogenomic data set as part of the 1KITE project as well as novel sequencing by the contributing labs covering 239 species from all 36 families of extant Orthoptera, and applying phylogenetic methods.

Molecular phylogenetic approaches are particularly relevant as the morphological level of homoplasies (e. g. repeated loss of wings) is likely high in Orthoptera which can make morphological data sets difficult to interpret. The strength of the present study is the thorough taxon coverage leading to the most robust molecular phylogeny elaborated to date with high support values for the nodes, often including species which have been studied in detail for behaviour and bioacoustics, and the timing of divergences by fossil calibration, lineage diversification, and mapping of morphological characters to address specific evolutionary modifications.

The results will be relevant for all researchers on Orthoptera and insects in general as reference for evolutionary relationships to interpret data, or even as help in choosing study groups for future research. The manuscript is written in a very clear way and, from this fairly broad topic, chooses several biological questions on the communication, ecology, and diversification for discussion.
A common evolutionary problem for Caelifera and Ensifera is the origin or multiple origins of the elaborate sound-producing structures and hearing organs presented as a "bird's-eye view" on the evolution of acoustic communication, the work obviously focuses on some topics over others, which however is justified as most phylogenetic analysis previously had insights on acoustic signalling at the centre of their analysis (Gwynne, 1995; Desutter-Grandcolas, 2003; Jost & Shaw, 2006), and keeps the manuscript concise. The figures provided are of very high quality, represent the large amount of information collected here very well, and will be instructive to the readers.

The resulting phylogeny with highest support re-confirms some well-established taxa like monophyletic Caelifera and Ensifera, but also indicates relationships which are remarkably gaining congruence to a previous morphology-based study, and it highlights the repeated origins and losses of specific structures, giving a relevant representation of the evolutionary dynamics of signalling/sensory structures. While it is obviously a simpler explanation that sound-producing structures evolved only once ancestrally, for the first time, ancestral state reconstruction for every node and the inclusion of structural data and considerations on the repeated origins of hearing organs in insects give a much more varied picture which is here supported by rigorous phylogenetic methods. The ancestral state reconstruction does not completely dissolve the ambiguities here (see l. 254), but importantly lends a quantitative basis to each of the scenarios. Hence, the results are highly important, thorough, and will become the standard reference in this field.
I recommend acceptance after a minor revision for some aspects to deepen the interpretation or make the presentation clearer:

Major

l. 39 the abstract is a very general summery of the findings, however it would be helpful if you spell out the main finding of "new light on the evolution of acoustic communication" more specifically, distinguishing e. g. between Caelifera and Ensifera, to emphasise how the results run against current paradigms.

l. 164 Given the involvement of wing movements in sound production, how do your phylogenetic findings relate to another theory that the wing movements ancestrally helped to spread pheromones for mate finding (e. g. in Bailey 1991, a reference cited in ms)? Could this be related to the evolution of stridulatory structures, or pre-date them? The reference also suggests that sound signalling probably derived from pheromone distribution via substrate vibration as main signalling channel to evoke another multi-step process of signal evolution. The role of wing venation is not elaborated here substantially, but supposedly it plays a great role in understanding the evolution of tegminal stridulation or wing-leg stridulation, considering the work of Bethoux, Arthropod Syst Phyl (2012) 70: 43.

l. 174 The analysis indicates now a greater congruence of relationships to those from a previous morphology-based phylogeny. On the other hand, molecular data sets have resulted in radically different placements e. g. of Tettigoniidae or Gryllidae. Can you comment or explain why your larger data set might have been required to gain some similarity between the two approaches?

l. 251 You state that hearing organs were present in the ancestral lineage of Anostostomatidae, but in the Fig. 3, the deep nodes in the anostostomatid species shows higher support for an organisation without tympana. Please resolve this, also keeping in mind that the suggested ancestral anostostomatid (Transaevum) has tympana (Johns, J Orthoptera Res (1997) 6: 125).

l. 312 This scenario on the origins of tympanal membranes seems to contradict the independent evolution in crickets and mole crickets presented by Desutter-Grandcolas (2003) Zool Scripta 32: 525 – could you elaborate this aspect and indicate why the convergent evolution does not find support in this case?

l. 399 within Acrididae, the diversification of stridulatory mechanisms seems to increase, including repeated evolution of certain mechanism – is there any behavioural or ecological correlation for this?

l. 412 The role of acoustic communication driving the orthopteran diversity seems somewhat underdeveloped. I'd suggest to move the introductory parts of this section into the introduction and formulate a hypothesis there. Since you state that such a test has not been carried out for insects, it might be appropriate to emphasise this issue. Since the acoustic signals are often species-specific, they could be expected to have an important role as key feature in radiations. The end of the section (l. 439) somewhat lacks a conclusive argument.

l. 522 With respect to acoustic communication, the fossils used for timing are not providing the same informations for sound-producing organs and hearing organs, as they do not cover tympana? This may be directly relevant to include in the discussion amid the difficulties to rule out tympana in the ancestral lineage of both main groups (l. 348).

Figures:
Figure 1 and 2 should share the same colour coding for identical BS values. Also, consider avoiding the green/red coding in favour of green/magenta colours, or similar.

Minor

l. 70 "…questions about the origins and original functions of these organs and how they have been modified through time remain inadequately addressed(4)."
It is not clear what you are aiming at here, especially as the sentence is supported by a single

vertebrate-specific reference. The follow-up continues with insect references and examples, so this linking sentence can already be shaped up addressing insect ears also. In this respect, the evolutionary reconstruction of the ear origins and modifications has a very clear concept, as it has been shown that the hearing organs are usually modified chordotonal organs which are structurally and functionally adapted to sound detection, see e. g. Stumpner, von Helversen (2001) Evolution and function of auditory systems in insects. Naturwissenschaften 88: 159 - who discuss that in Orthoptera, the initial predation detection is accepted for grasshoppers, but not for crickets - or Göpfert, Hennig (2016) Annu Rev Entomol 61: 257, Yack (2004) Microsc Res Tech 63: 315. Given the structural elements elaborated in hearing organs, the multiple evolutionary origins are a possibility in the light of the adaptation of different already existing elements.
l. 147 a very similar number is given earlier (l. 85) for all Orthoptera?
l. 189 Include a reference for the venation homology
l. 217 Consider connecting the time information more closely by moving the origin time up in the sentence: "…and the earliest diverging lineage originated in the Jurassic and includes monkey grasshoppers…"
l. 253 How would sound generation have been detected then?
l. 258, 352 state the test again together with the p value?
l. 303 when you speak about bidirectional hearing in the ancestral Ensifera here, does this conflict with the repeated evolution of tympana in separate lineages presented before
(l. 250)? In other words, is the support for the ancestral presence of tympana actually stronger?
l. 313 Do you base these identifications on homology assessment?
l. 347 Can you discuss how this relates to the origins of tympana inferred e. g. by Flook et al. (2000) Mol Phylogenet Evol 15: 345
l. 362 Do Caelifera also use vibratory signals, for example in the non-hearing species?
l. 379 some support for this correlation has been published by comparison of different species: Riede et al. (1990) Zoomorphology 109: 223, and Lehmann et al. (2010) J Comp Physiol A 196: 807.
l. 395 species-specific sounds: is there a reference in support for this, or other (unpublished) evidence?
l. 428 double
l. 542 are these phylogenetic independent contrasts?
l. 821 TO: tympanal organ, as in l. 311?

Reviewer #2 (Remarks to the Author):

In this manuscript, the authors generate a large-scale time-calibrated phylogeny for orthopteran insects based on phylogenomic data. They then use this phylogeny to analyze the evolution of acoustic communication. They test for (and find) significant associations between the evolution of acoustic communication and hearing. They also test for an association between acoustic communication and increases in diversification rates, but do not find an association.

Overall, I found this manuscript to be interesting and potentially appropriate for Nature Communications. I found the new phylogenomic analysis and the analyses of communication were valuable separately, but together they make for a relatively strong paper.

However, I do have several concerns about the manuscript as presently written, which the authors should address before the paper is published. I list these below.

1) I was not that happy with the way that the manuscript is written. It is not that unusual to combine results and discussion. However, as the manuscript is presently written, it can be quite hard to distinguish: (a) results of the present paper, (b) results of previous papers, and (c) speculation based on "a" or "b". The inability to distinguish "a" and "c" is particularly problematic. I think that a good

solution to this problem would be to rewrite it to clearly distinguish the results of this particular study and discussion based on these results (i.e. have a separate Results and Discussion section). This will force the authors to clarify what their actual results are, and which results are from previous studies (or speculation).

2) Along these lines, the manuscript is a serious chore to read. For example, there are long unbroken paragraphs in long unbroken sections. Paragaphs are hard to follow, and so are the sections. Dividing the manuscript into additional sections and subsections could help (i.e. Results with different subheadings within it; Discussion with different subheadings within it). Subdividing long paragraphs could also help.

3) Along these lines, I had to read the supplementary files to get basic information that should have been present in the main text. For example, there is no mention in the main text of the method used to estimate the phylogenies. Similarly, the results do not really contain the results of the tests of correlated evolution between sound production and hearing (the results reported in the Supplementary Information are overall quite different from those reported in the main text). Again, I think that the paper needs to be extensively rewritten to clarify what was done and what the results were.

4) Although testing for an association between acoustic communication and diversification is one of the stated goals of the paper, there is not really a test of this association in the paper. Basically, the authors reconstruct trait evolution and diversification rate shifts on the phylogeny, but there is no statistical test for an association between them. That is not acceptable. In fact, there are methods that are explicitly designed to test for an association between diversification rates and traits (like BiSSE/HiSSE). BAMM is not one of those methods. Moreover, BAMM is actually quite controversial, but there is no mention here of the papers that have found serious problems with it (e.g. Moore et al. 2016; PNAS; Meyer and Wiens 2018; Evolution). The developer of BAMM has responded to these papers, but has not actually fixed the problems. Indeed, many of the problems pointed out in these papers were apparent in the original paper in which BAMM was proposed. In addition, the description of these BAMM analyses in the present paper is not very detailed, even in the Supplementary Information.

5) It is a bit troubling that there are transcriptomic data for only 60 taxa, and only mitochondrial data for 189 of the 249 included taxa. It would be good to know if, for the 60 taxa with overlapping data, the mitogenomic data alone give a good approximation of the tree from transcriptomic data alone. If not, I would be concerned about those parts of the tree with no transcriptomic data (based only on mitochondrial data). For example, many of the basal divergences in Caelifera appear to be only weakly supported.

6) The authors trace the evolution of sound production and hearing, but talk about the evolution of acoustic communication (e.g. in the title). There can be both sound production and hearing without acoustic communication, so it would be good to clarify this.

Reviewer #3 (Remarks to the Author):

Review of manuscript 'Phylogenomic analysis of Orthoptera sheds new light on the evolution of acoustic communication' by H. Song et al.

This manuscript focuses on an important subject in evolutionary biology and animal behavior. The authors have conducted a very thorough analysis of the phylogeny of this group and how acoustic communication evolved differently in different clades, and the article should be published following

some revisions. My main recommendations are : 1) As currently written, the article appears to have two main objectives, developing a reliable phylogeny of the Orthoptera and discerning the evolution of acoustic communication. The authors may not intend that their manuscript focuses simply on a new and thorough phylogeny, but so much space (lines 107-240) and detail are devoted to this objective that a reader is likely to lose sight the ultimate aim. The authors should clarify more strongly that the new phylogeny is needed in order to elucidate the evolution of acoustic communication, and this might be aided by relegating some of the phylogenetic details to supplementary material. Also, a brief resume in the text reporting the main differences between the current phylogeny and previous ones would be helpful. 2) Numerous inferences need to be reconsidered in light of other information and possibilities.

Line 50 ‘easily recognisable’ - do you mean conspicuous to a human observer ? Thus, unlikely to be unnoticed, especially given the availability of instruments for sensing ultrasound, etc.

Line 60 The statement about vertebrate ears is somewhat misleading, and the reference (6) is not appropriate. In vertebrates, all inner ear structures are thought to have descended from the same structure that evolved in the Devonian (400 MYA) (Popper, A. N., Platt, C., & Edds, P. L. (1992). Evolution of the vertebrate inner ear: An overview of ideas. In D. B. Webster, R. R. Fay, & A. N. Popper (Eds.), The evolutionary biology of hearing (pp. 49–57). New York: Springer-Verlag). Later, different external and affiliated structures evolved repeatedly and often independently.

Line 63 Listing Embioptera among the hearing orders is premature at this point. Reference 12 indicates an external region that resembles a simple tympanum, but there is no other information, behavioral or physiological, that supports a hearing function of this region (device ?).

Line 66 I am unaware of any sound-producing structure, or sound for that matter, in the Mantodea. In any case, reference 10 does not list Mantodea as sound producing.

Line 76 Regarding reference 16, it may be premature to defintively accept its proposition that Lepidopteran hearing evolved prior to the appearance of insectivorous bats (ca 65 MYA). A careful reading of figure 1 in this reference indicates that the confidence limits of most of the critical nodes are wide and extend to dates more recent than 65 MYA ; this includes the nodes with fossil calibration. It certainly is curious that so many lepidopteran, as well as other insect groups (Coleoptera, Neuroptera), evolved ultrasound perception at approximately the same time in evolutionary history. What pressure appeared 90 MYA ? Here, Mantodea and one lepidopteran group (Tineidae) seem to be exceptions, having developed ears well before the Paleogene.

Line 78 ‘ephemeral’ Do you basically mean that behavior – hearing or producing sound, for example – may not leave fossil evidence and thus its history is difficult to interpret ?

Line 86 delete ‘known’

Lines 92-93 Nonetheless, one should reference those works that focused separately on Caelifera and on Ensifera (see references listed in reference 10)

Line 120 ‘topographical’ - do you mean topological ?

Line 124 ‘not particularly relevant’ - This is one case where more detail would be helpful. How do you justify ignoring these ‘conflicts’ ?

Line 144 four main extant lineages

Lines 252-257 Is it possible that a less conspicuous, e.g. internal chordotonal organ with auditory function, originated once in Ensifera, with separate external (specialized tympana) evolving in the

several lineages shortly thereafter ? The initial and single origin of tegminal-tegminal stridulation in the Ensifera is likely to have been sexual, not a predator deterrent : I am unaware of any evidence suggesting that these male stridulatory devices were originally found in both sexes and then lost in the females. Note : the few cases of female stridulation in the Ensifera involve structures that are not homologous with the male file and scraper.

***

General point : Given that the main objective of this paper is evolution of acoustic communication in the Orthoptera, it would be helpful to present the general theories on the evolution of sexual signaling : 1) Signals evolve via 'sensory bias' wherein one sex, generally the male, develops signals that the other sex, generally the female, already has perception for and in a general, non-sexual context. 2) Signals (in males) and perception and preference (in females) co-evolve with each other from their origin ; the so-called Fisherian mechanism. Can you infer which mechanism(s) were likely responsible for the evolution of acoustic communication in the several orthopteran lineages ? See reference 10.

Line 294 'peculiar position' - explain

Lines 294-296 Your suggestion here reflects my comment on lines 252-257 ; hearing and stridulation coevolved early, and all were functioning in a sexual, rather than generalized, context.

Line 304 Again, more evidence favoring early coevolution.

Line 329 'comes'

Line 336 'show'

Line 379 Regarding the connection between flight and hearing in insects, it is interesting to note that hearing is only found in groups that fly or are secondarily flightless (e.g. brachypterous tettigoniids). However, some very aerial groups have little or no hearing. One might infer that flight is a necessary precursor because the air chamber in tympanal ears is derived from the tracheal system, and aerial insects will have more developed trachea to support their higher oxygen demands. But devoting a major portion of the tracheal system to perception rather than supplying oxygen may not have been economically sustainable in the most aerial insects that fly very often and with high wingbeat rhythms (e.g. Hymenoptera, Diptera, Odonata). Additionally, those groups that truly excel in flight have, by necessity, very well developed eyes, and the evolution of a second main perception – hearing – may not have been necessary – save in nocturnally active species – and might have detracted from visual ability. See Greenfield, M. D. (2002). Signalers and receivers: Mechanisms and evolution of arthropod communication. New York: Oxford University Press ; page 223 and pages 219-229 in general).

Line 412 - While it might be said that sexual selection is a likely factor in speciation (general reference should be given - MJ West Eberhard, e.g.), there is no reason to expect sexual selection to be a greater factor in singing insects than in groups relying on other signaling modalities, eg. Visual display as in butterflies. That is, there is nothing special about acoustic signaling in comparison with other displays.

Line 470 'group'

Line 471 Origin in an anti-predator context is unlikely given that these stridulatory structures are male and there is no evidence that presence in females was lost secondarily. See comments for lines 252-257.

Figure 1 Having the illustrations on the right side of the figure is very helpful, but it would be much

more helpful if the specific figures were connected to specific groups (clades).

Figure 4 Using Pagel's test is most appropriate, but one wonders how it would reflect the possible joint origin of hearing and singing in Ensifera ; see comments on lines 252-257 and the general point on Fisherian coevolution.

Response to reviewers' comments

Below we provide our responses to the reviewers' comments in blue. I have incorporated most of the suggested changes as well as additional analyses. The manuscript has now gone through an extensive revision and reorganization, and we sincerely hope that this revision is satisfactory to the reviewers.

Reviewer #1 (Remarks to the Author):

"Phylogenomic analysis of Orthoptera sheds new light on the evolution of acoustic communication"
by Song et al., submitted to NATURE COMMUNICATIONS

General:
Orthoptera provide important model species for the study of animal behaviour and communication, sexual selection, speciation, and physiology. While it is hard to overestimate their importance for biological research, evolutionary analyses are also hampered by the lack of a robust phylogeny at the higher taxon level. The morphological and molecular phylogenies published over the last 20 years resulted in contradicting relationships, or lacked rigorous distinction of coding between the species level and higher taxonomic levels. The manuscript submitted by Song et al. addresses this phylogenetic problem with a phylogenomic data set as part of the 1KITE project as well as novel sequencing by the contributing labs covering 239 species from all 36 families of extant Orthoptera, and applying phylogenetic methods.

Molecular phylogenetic approaches are particularly relevant as the morphological level of homoplasies (e. g. repeated loss of wings) is likely high in Orthoptera which can make morphological data sets difficult to interpret. The strength of the present study is the thorough taxon coverage leading to the most robust molecular phylogeny elaborated to date with high support values for the nodes, often including species which have been studied in detail for behaviour and bioacoustics, and the timing of divergences by fossil calibration, lineage diversification, and mapping of morphological characters to address specific evolutionary modifications.

The results will be relevant for all researchers on Orthoptera and insects in general as reference for evolutionary relationships to interpret data, or even as help in choosing study groups for future research. The manuscript is written in a very clear way and, from this fairly broad topic, chooses several biological questions on the communication, ecology, and diversification for discussion.

A common evolutionary problem for Caelifera and Ensifera is the origin or multiple origins of the elaborate sound-producing structures and hearing organs presented as a "bird's-eye view" on the evolution of acoustic communication, the work obviously focuses on some topics over others, which however is justified as most phylogenetic analysis previously had insights on acoustic signalling at the centre of their analysis

(Gwynne, 1995; Desutter-Grandcolas, 2003; Jost & Shaw, 2006), and keeps the manuscript concise. The figures provided are of very high quality, represent the large amount of information collected here very well, and will be instructive to the readers.

The resulting phylogeny with highest support re-confirms some well-established taxa like monophyletic Caelifera and Ensifera, but also indicates relationships which are remarkably gaining congruence to a previous morphology-based study, and it highlights the repeated origins and losses of specific structures, giving a relevant representation of the evolutionary dynamics of signalling/sensory structures. While it is obviously a simpler explanation that sound-producing structures evolved only once ancestrally, for the first time, ancestral state reconstruction for every node and the inclusion of structural data and considerations on the repeated origins of hearing organs in insects give a much more varied picture which is here supported by rigorous phylogenetic methods. The ancestral state reconstruction does not completely dissolve the ambiguities here (see l. 254), but importantly lends a quantitative basis to each of the scenarios. Hence, the results are highly important, thorough, and will become the standard reference in this field. I recommend acceptance after a minor revision for some aspects to deepen the interpretation or make the presentation clearer:

We appreciate Reviewer 1’s very positive comments and that the reviewer found our manuscript to be clear and highly important.

Major

l. 39 the abstract is a very general summery of the findings, however it would be helpful if you spell out the main finding of “new light on the evolution of acoustic communication” more specifically, distinguishing e. g. between Caelifera and Ensifera, to emphasise how the results run against current paradigms.

We have completely re-written the abstract to reflect the new finding and the discussion. Given the 150-word limit, we could not highlight everything, but the newly written abstract is now more specific than the previous one.

l. 164 Given the involvement of wing movements in sound production, how do your phylogenetic findings relate to another theory that the wing movements ancestrally helped to spread pheromones for mate finding (e. g. in Bailey 1991, a reference cited in ms)? Could this be related to the evolution of stridulatory structures, or pre-date them? The reference also suggests that sound signalling probably derived from pheromone distribution via substrate vibration as main signalling channel to evoke another multi-step process of signal evolution. The role of wing venation is not elaborated here substantially, but supposedly it plays a great role in understanding the evolution of tegminal stridulation or wing-leg stridulation, considering the work of Bethoux, Arthropod Syst Phyl (2012) 70: 43.

While some orthopterans rely on cuticular hydrocarbon as a chemical cue, there is no strong evidence that orthopteran insects have seriously relied on volatile pheromone for

sexual communication. We have carefully thought about the merit of the theory mentioned by the reviewer, but decided that it does not fit with the data and the patterns observed in the study, so we did not include it in the text. We argue that the stridulatory apparatus probably evolved in the context of producing defensive signal against vertebrate predators in crown-Orthoptera. We also argue that the ancestral Ensifera already had the ability to move the wings as a plesiomorphic trait based on the fossil evidence of other crown-orthopterans also having stridulatory tegmina, although the specific stridulatory files were non-homologous to those of Ensifera.

l. 174 The analysis indicates now a greater congruence of relationships to those from a previous morphology-based phylogeny. On the other hand, molecular data sets have resulted in radically different placements e. g. of Tettigoniidae or Gryllidae. Can you comment or explain why your larger data set might have been required to gain some similarity between the two approaches?

Previous studies used either nuclear ribosomal RNA genes (Jost and Shaw 2006) or mitochondrial genomes (Song et al. 2015), and a close inspection of these papers reveals that the nodal support values for higher-level relationships within Ensfera were not high. Some of these markers had been shown to be affected by non-stationarity and heterotachy, which could have affected the resulting relationships. It is also likely that the previously used markers did not have enough phylogenetic signal to robustly resolve deep relationships. Our current study used phylogenomic data and we performed a rigorous analysis of the phylogenetic signal in our datasets. The differences in topology between our current study compared to the previous studies reflect the improvement in our understanding and the robustness of our dataset.

l. 251 You state that hearing organs were present in the ancestral lineage of Anostostomatidae, but in the Fig. 3, the deep nodes in the anostostomatid species shows higher support for an organisation without tympana. Please resolve this, also keeping in mind that the suggested ancestral anostostomatid (Transaevum) has tympana (Johns, J Orthoptera Res (1997) 6: 125).

Our taxon sampling for Anostostomatidae (only 4 taxa included) is not comprehensive enough to make a definitive inference about the evolution of tympana in this family. According to our Figure 3, it is true that there is a higher probability for the absence of hearing organs for Anostostomatidae, which is caused by one of the four included taxa not having the tympana. What we know from the family is that the presence of tympana is widespread and likely to be ancestral. In order to fully resolve this, we will need to expand the taxon sampling for this group a lot, which we are working on right now as a part of ongoing project, but it could not be incorporated into the manuscript.

l. 312 This scenario on the origins of tympanal membranes seems to contradict the independent evolution in crickets and mole crickets presented by Desutter-Grandcolas (2003) Zool Scripta 32: 525 – could you elaborate this aspect and indicate why the convergent evolution does not find support in this case?

Based on a careful reading of Desutter-Grandcolas (2003), it is clear that her idea of the independent evolution of tympanal membranes in crickets and mole crickets is largely influenced by her hypothesis that the stridulatory apparatus in these two groups are not homologous. Unfortunately, her study does not give any morphological evidence of two independent evolution of tympana in crickets and mole crickets. Based on her character optimization, she could have easily interpreted the same result as a single origin of tympana in the common ancestor of Grylloidea and Gryllotalpoidea. Based on our own research, we also cannot find any morphological or phylogenetic evidence that suggest tympana being not homologous between the two. Therefore, we think that there is not much to discuss about the point that the reviewer has raised.

l. 399 within Acrididae, the diversification of stridulatory mechanisms seems to increase, including repeated evolution of certain mechanism – is there any behavioural or ecological correlation for this?

It is not clear what the reviewer means by "the diversification of stridulatory mechanisms seems to increase." Within Acrididae, we did find a few different stridulatory mechanisms, but most of them are concentrated on a clade consisting of Acridinae, Gomphocerinae, and Oedipodinae. We think that the use of hind femora to produce sound is not unique to Acrididae as it has repeatedly evolved in Pamphagidae, Pneumoridae, and even in Ensifera. Ecologically speaking these singing acridids appeared to have diversified along with the diversification of grasslands, but how that could have influenced a particular sound producing mechanism is not entirely clear. In this revision, we have focused on the evolution of hearing and sound producing organs, and the revised text does not have this section any more.

l. 412 The role of acoustic communication driving the orthopteran diversity seems somewhat underdeveloped. I'd suggest to move the introductory parts of this section into the introduction and formulate a hypothesis there. Since you state that such a test has not been carried out for insects, it might be appropriate to emphasise this issue. Since the acoustic signals are often species-specific, they could be expected to have an important role as key feature in radiations. The end of the section (l. 439) somewhat lacks a conclusive argument.

Thanks for this comment. We have considerably revised the introduction so that there are clear hypotheses to test, and we have tightened the text to be focused on the evolutionary dynamics of hearing and sound producing organs. Furthermore, we have added trait-based diversification models in the revision, which specifically tests whether the evolution of acoustic communication has affected the net diversification rate. We find that it did not, and this is indeed one of the major findings of our study.

l. 522 With respect to acoustic communication, the fossils used for timing are not providing the same informations for sound-producing organs and hearing organs, as they do not cover tympana? This may be directly relevant to include in the discussion

amid the difficulties to rule out tympana in the ancestral lineage of both main groups (l. 348).

In order to estimate divergence times, we need reliable fossil calibration points. We have performed extensive topographical homology conjecture of many available fossils and applied rigorous criteria to select the fossils used for our analysis. Of the 11 fossils used for calibration, 6 were orthopterans. Of these, one was of crown-Orthoptera, 3 were ensiferans, and two were caeliferans. The ensiferan fossils include clear wing veins, and thus we can infer the antiquity of acoustic communication in Ensifera. However, the reviewer is correct that other fossils do not include features relevant for either hearing or sound production. Therefore, the sole purpose of divergence time estimation is to provide a temporal perspective to our study, and the inferences related to the evolution of hearing and sound producing organs came entirely from the ancestral character state reconstruction based on examination of the extant taxa. Our revised discussion explicitly notes the uncertainty associated with our current analysis.

Figures:
Figure 1 and 2 should share the same colour coding for identical BS values. Also, consider avoiding the green/red coding in favour of green/magenta colours, or similar.

Thanks for catching this. We have made the BS value colour scheme consistent. We kept our colour scheme for the phylogeny as is.

Minor

l. 70 "…questions about the origins and original functions of these organs and how they have been modified through time remain inadequately addressed(4)."
It is not clear what you are aiming at here, especially as the sentence is supported by a single vertebrate-specific reference. The follow-up continues with insect references and examples, so this linking sentence can already be shaped up addressing insect ears also. In this respect, the evolutionary reconstruction of the ear origins and modifications has a very clear concept, as it has been shown that the hearing organs are usually modified chordotonal organs which are structurally and functionally adapted to sound detection, see e. g. Stumpner, von Helversen (2001) Evolution and function of auditory systems in insects. Naturwissenschaften 88: 159 - who discuss that in Orthoptera, the initial predation detection is accepted for grasshoppers, but not for crickets - or Göpfert, Hennig (2016) Annu Rev Entomol 61: 257, Yack (2004) Microsc Res Tech 63: 315. Given the structural elements elaborated in hearing organs, the multiple evolutionary origins are a possibility in the light of
the adaptation of different already existing elements.

Thanks for the suggestion. We have extensively revised the introduction and largely focused on insects. The new introduction is now clearer and more focused.

l. 147 a very similar number is given earlier (l. 85) for all Orthoptera?

The number in the introduction "the number of orthopteran species that primarily use acoustic signaling as a main mode of sexual communication (~16,000 spp.)" refers to all singing orthopterans from both suborders. Orthoptera overall contains about 28,000 spp. The number in the discussion (~15,500 spp.) is the total number of extant species within Ensifera.

l. 189 Include a reference for the venation homology

This part has been moved to Supplementary Information and we have provided a reference within SI.

l. 217 Consider connecting the time information more closely by moving the origin time up in the sentence: "…and the earliest diverging lineage originated in the Jurassic and includes monkey grasshoppers…"

We have moved this section to Supplementary Information.

l. 253 How would sound generation have been detected then?

Our study lays out a scenario that sound producing organs were ancestral (plesiomorphic) for Ensifera, and tibial tympana evolved multiple times. The ensiferan ears consist of internal hearing sensory organs and external tympana. It is unclear what the original form of sound detection was, but it is conceivable that thin cuticle of foretibia could have functioned as a resonator for the internal sensory organs to pick up the sound wave. We infer that thinning of the cuticle evolved multiple times to give rise to tympanal membrane within Ensifera.

l. 258, 352 state the test again together with the p value?

Our test was Pagel's character correlation test. Instead of presenting the p-values, we have included the weighted AIC scores for the best fitting models for each analysis.

l. 303 when you speak about bidirectional hearing in the ancestral Ensifera here, does this conflict with the repeated evolution of tympana in separate lineages presented before (l. 250)? In other words, is the support for the ancestral presence of tympana actually stronger?

Externally tibial tympana look similar and they are in the same position, but internally the evolution of hearing sensory organs (SGO, IO, TO, CAH, CA) shows a step-wise pattern. We do not doubt that the evolution of tibial tympana is ancient. However, without fossil evidence, and based on inferences solely on the extant taxa, it is unclear whether the tibial tympana evolved in the common ancestor of all Ensifera. Thus, we have to go by the inference allowed by the ancestral character state reconstruction. We have included a sentence in the main text expressing the uncertainty.

l. 313 Do you base these identifications on homology assessment?

No. Because we do not have neuroanatomical data across our entire taxon sampling, we made an assumption that these neuroanatomical features are conserved at the level of family. So, we simply optimized the characters on the tree, without ancestral character state reconstruction. This is clearly explained in the Methods section.

l. 347 Can you discuss how this relates to the origins of tympana inferred e. g. by Flook et al. (2000) Mol Phylogenet Evol 15: 345

We have since revised this sentence to be clearer. We infer that abdominal tympana evolved multiple times based on ancestral character state reconstruction and that it dated back to the Cretaceous when Pyrgomorphidae originated. Flook et al. (2000) put forth the first study on the phylogeny of basal Caeliferans and included two hypotheses on the origin of tympana, which they correlated with the evolution of stridulation. The first was the coevolution of hearing and sound producing organs followed by the repeated losses of sound producing organs, and the second was the evolution of tympana to detect predators (especially the bats). Our study has considered what the phylogenetic pattern would look like in both cases, and our results are consistent with the separate evolutionary trajectories of hearing and sound producing organs in Caelifera and later evolution of hearing organs, possibly in the context of flight modulation. We cited Flook et al. (2000) in discussing the possibilities, but did not elaborate too much since their ideas were already incorporated in the hypotheses (which were tested) also proposed by others.

l. 362 Do Caelifera also use vibratory signals, for example in the non-hearing species?

Vibratory signalling is mostly unknown in Caelifera. There are some rudimentary studies suggesting its existence in Tetrigidae and even in Eumastacidae, but without much experimental data. Since our study focused on acoustic signalling, we did not elaborate on this aspect in the manuscript.

l. 379 some support for this correlation has been published by comparison of different species: Riede et al. (1990) Zoomorphology 109: 223, and Lehmann et al. (2010) J Comp Physiol A 196: 807.

Thanks for letting us know about these papers. We have included them in the reference.

l. 395 species-specific sounds: is there a reference in support for this, or other (unpublished) evidence?

We have included a citation.

l. 428 double

Corrected.

l. 542 are these phylogenetic independent contrasts?

As we used discrete traits, we did not use phylogenetic independent contrasts.

l. 821 TO: tympanal organ, as in l. 311?
Corrected.

Reviewer #2 (Remarks to the Author):

In this manuscript, the authors generate a large-scale time-calibrated phylogeny for orthopteran insects based on phylogenomic data. They then use this phylogeny to analyze the evolution of acoustic communication. They test for (and find) significant associations between the evolution of acoustic communication and hearing. They also test for an association between acoustic communication and increases in diversification rates, but do not find an association.

Overall, I found this manuscript to be interesting and potentially appropriate for Nature Communications. I found the new phylogenomic analysis and the analyses of communication were valuable separately, but together they make for a relatively strong paper.

We appreciate the reviewer's positive attitude toward our manuscript.

However, I do have several concerns about the manuscript as presently written, which the authors should address before the paper is published. I list these below.

We have carefully considered each of the comments and revised the manuscript accordingly.

1) I was not that happy with the way that the manuscript is written. It is not that unusual to combine results and discussion. However, as the manuscript is presently written, it can be quite hard to distinguish: (a) results of the present paper, (b) results of previous papers, and (c) speculation based on "a" or "b". The inability to distinguish "a" and "c" is particularly problematic. I think that a good solution to this problem would be to rewrite it to clearly distinguish the results of this particular study and discussion based on these results (i.e. have a separate Results and Discussion section). This will force the authors to clarify what their actual results are, and which results are from previous studies (or speculation).

Although we think that this comment has more to do with the reviewer's preference than the actual merit of the manuscript, we agree that the way it was originally written could have been confusing in terms of distinguishing our results from previous results. Therefore, we have completely revised our manuscript and it now has separate results

and discussion sections. We think that this new organization flows better and we appreciate the reviewer’s suggestion.

2) Along these lines, the manuscript is a serious chore to read. For example, there are long unbroken paragraphs in long unbroken sections. Paragaphs are hard to follow, and so are the sections. Dividing the manuscript into additional sections and subsections could help (i.e. Results with different subheadings within it; Discussion with different subheadings within it). Subdividing long paragraphs could also help.

Given the fact that other reviewers found our manuscript to be clear and easy to follow, this comment also seems to reflect the reviewer’s personal preference. However, took the reviewer’s comments seriously and made sure that the long paragraphs were broken into digestible sections.

3) Along these lines, I had to read the supplementary files to get basic information that should have been present in the main text. For example, there is no mention in the main text of the method used to estimate the phylogenies. Similarly, the results do not really contain the results of the tests of correlated evolution between sound production and hearing (the results reported in the Supplementary Information are overall quite different from those reported in the main text). Again, I think that the paper needs to be extensively rewritten to clarify what was done and what the results were.

We have expanded the method section and moved some of the methods from the Supplementary Information to the main text.

4) Although testing for an association between acoustic communication and diversification is one of the stated goals of the paper, there is not really a test of this association in the paper. Basically, the authors reconstruct trait evolution and diversification rate shifts on the phylogeny, but there is no statistical test for an association between them. That is not acceptable. In fact, there are methods that are explicitly designed to test for an association between diversification rates and traits (like BiSSE/HiSSE). BAMM is not one of those methods. Moreover, BAMM is actually quite controversial, but there is no mention here of the papers that have found serious problems with it (e.g. Moore et al. 2016; PNAS; Meyer and Wiens 2018; Evolution). The developer of BAMM has responded to these papers, but has not actually fixed the problems. Indeed, many of the problems pointed out in these papers were apparent in the original paper in which BAMM was proposed. In addition,
the description of these BAMM analyses in the present paper is not very detailed, even in the Supplementary Information.

We appreciate this comment and we have now performed HiSSE and incorporated the results into the study. We are well aware of the controversies surrounding BAMM. In fact, we believe that most of the diversification analyses available today have some inherent issue (some methodological and some philosophical). However, BAMM still has merits as it can detect rate shifts and calculate clade-specific diversification rate, so as a tool to understand the general pattern of diversification, it has its own utility. So, we

kept the analysis and figure as is. The reviewer is correct that BAMM is not a right approach if we are to test trait-dependent diversification. For binary traits, BiSSE and HiSSE are available, with HiSSE being a more robust method since it can account for hidden states. So, in our study, we used HiSSE in a multimodel inference framework and fitted 24 different models of trait-based diversification. These 24 models were identical to those used by the developers of HiSSE in their 2016 paper. Of the 24 models, 4 models correspond to BiSSE with various parameter combinations, 16 models were HiSSE models with various parameter combinations. 4 models were what is called the trait-independent diversification models. Our collective results suggest the best fitting model for the evolution of acoustic communication was one of the trait-independent diversification, suggesting that the evolution of acoustic communication was independent of the species diversification. This inference is in fact in line with our original finding, hypothesized based on BAMM results. Now, we have both analyses that suggest the same patter, which we think makes our study stronger.

5) It is a bit troubling that there are transcriptomic data for only 60 taxa, and only mitochondrial data for 189 of the 249 included taxa. It would be good to know if, for the 60 taxa with overlapping data, the mitogenomic data alone give a good approximation of the tree from transcriptomic data alone. If not, I would be concerned about those parts of the tree with no transcriptomic data (based only on mitochondrial data). For example, many of the basal divergences in Caelifera appear to be only weakly supported.

Certainly, more data are better. However, this study was originally conceived as a subproject for 1KITE, and the taxon sampling for transcriptome was pre-determined. We view our approach, combining transcriptome data with mitochondrial genome data as a strength. Because of the exact concern by the reviewer, we have performed extensive tests vetting the data, and what we present in the paper is the best possible way to analyse this particular dataset. We have also extensively analysed mitochondrial genome data only, and they indeed give a good approximation of the tree presented in the manuscript. The reviewer is incorrect that the support for the basal nodes for Caelifera is weak. In all of the analyses, higher-level relationships within Caelifera are strongly supported. The low support is found at the shallow-level relationships, but this has little implication for the broad evolutionary patterns discussed in this paper.

6) The authors trace the evolution of sound production and hearing, but talk about the evolution of acoustic communication (e.g. in the title). There can be both sound production and hearing without acoustic communication, so it would be good to clarify this.

We appreciate this comment and we have refocused the manuscript so that we are talking mostly about the evolution of hearing and sound producing organs. We left the title as is, because ultimately the paper is about the evolution of acoustic communication.

Reviewer #3 (Remarks to the Author):

Review of manuscript ‘Phylogenomic analysis of Orthoptera sheds new light on the evolution of acoustic communication’ by H. Song et al.

This manuscript focuses on an important subject in evolutionary biology and animal behavior. The authors have conducted a very thorough analysis of the phylogeny of this group and how acoustic communication evolved differently in different clades, and the article should be published following some revisions. My main recommendations are : 1) As currently written, the article appears to have two main objectives, developing a reliable phylogeny of the Orthoptera and discerning the evolution of acoustic communication. The authors may not intend that their manuscript focuses simply on a new and thorough phylogeny, but so much space (lines 107-240) and detail are devoted to this objective that a reader is likely to lose sight the ultimate aim. The authors should clarify more strongly that the new phylogeny is needed in order to elucidate the evolution of acoustic communication, and this might be aided by relegating some of the phylogenetic details to supplementary material. Also, a brief resume in the text reporting the main differences between the current phylogeny and previous ones would be helpful. 2) Numerous inferences need to be reconsidered in light of other information and possibilities.

We appreciate the reviewer’s comment. This comment helped us reshape the introduction and reorganize the manuscript. We have completely revised the manuscript this and other reviewer comments, and it should now be satisfactory to the reviewer.

Line 50 ‘easily recognisable’ - do you mean conspicuous to a human observer ? Thus, unlikely to be unnoticed, especially given the availability of instruments for sensing ultrasound, etc.

We have changed this word to conspicuous.

Line 60 The statement about vertebrate ears is somewhat misleading, and the reference (6) is not appropriate. In vertebrates, all inner ear structures are thought to have descended from the same structure that evolved in the Devonian (400 MYA) (Popper, A. N., Platt, C., & Edds, P. L. (1992). Evolution of the vertebrate inner ear: An overview of ideas. In D. B. Webster, R. R. Fay, & A. N. Popper (Eds.), The evolutionary biology of hearing (pp. 49–57). New York: Springer-Verlag). Later, different external and affiliated structures evolved repeatedly and often independently.

We have clarified this part and cited the recommended citation.

Line 63 Listing Embioptera among the hearing orders is premature at this point. Reference 12 indicates an external region that resembles a simple tympanum, but there is no other information, behavioral or physiological, that supports a hearing function of this region (device ?).

We agree that the presence of hearing organs in Embioptera is premature. We initially included it to be comprehensive, but the reviewer is correct that there is no experimental data for their hearing. So, we have removed the citation, and we have also removed it from our ancestral character state reconstruction.

Line 66 I am unaware of any sound-producing structure, or sound for that matter, in the Mantodea. In any case, reference 10 does not list Mantodea as sound producing.

Mantids have stridulatory organs. We have included this citation: Hill, S. A. Sound generation in Mantis religiosa (Mantodea: Mantidae): stridulatory structures and acoustic signal. J. Orthoptera Res. 16, 35-49 (2007).

Line 76 Regarding reference 16, it may be premature to defintively accept its proposition that Lepidopteran hearing evolved prior to the appearance of insectivorous bats (ca 65 MYA). A careful reading of figure 1 in this reference indicates that the confidence limits of most of the critical nodes are wide and extend to dates more recent than 65 MYA ; this includes the nodes with fossil calibration. It certainly is curious that so many lepidopteran, as well as other insect groups (Coleoptera, Neuroptera), evolved ultrasound perception at approximately the same time in evolutionary history. What pressure appeared 90 MYA ? Here, Mantodea and one lepidopteran group (Tineidae) seem to be exceptions, having developed ears well before the Paleogene.

Our reason for including this reference (Kawahara et al. 2019) was because it was one of the recently published study also using 1KITE data that deal with the evolution of acoustic communication in Lepidoptera. That paper presented an evolutionary scenario that hearing in Lepidoptera evolved prior to the evolution of bats, and like any scientific hypothesis, it is subject to testing and possible criticism. Our study shows that the evolution of hearing and sound producing organs both in both suborders were ancient, certainly pre-dating the bats. So, we have since edited the sentence and make no reference to bat avoidance.

Line 78 ‘ephemeral’ Do you basically mean that behavior – hearing or producing sound, for example – may not leave fossil evidence and thus its history is difficult to interpret ?

We have removed this sentence as we completely re-wrote the introduction.

Line 86 delete ‘known’

Deleted.

Lines 92-93 Nonetheless, one should reference those works that focused separately on Caelifera and on Ensifera (see references listed in reference 10)

We have removed this sentence as we completely re-wrote the introduction.

Line 120 ‘topographical’ - do you mean topological ?

Topographical is correct

Line 124 ‘not particularly relevant’ - This is one case where more detail would be helpful. How do you justify ignoring these ‘conflicts’ ?

We have revised this sentence as “Nevertheless, the ambiguous placements of these two latter families had little impact in inferring the evolution of hearing and sound producing organs.” We did not ignore this issue; instead we considered whether the inference regarding the evolution of hearing and sound producing organs would be affected by the ambiguity. We concluded that it did not, which justifies this sentence.

Line 144 four main extant lineages

Corrected.

Lines 252-257 Is it possible that a less conspicuous, e.g. internal chordotonal organ with auditory function, originated once in Ensifera, with separate external (specialized tympana) evolving in the several lineages shortly thereafter ? The initial and single origin of tegminal-tegminal stridulation in the Ensifera is likely to have been sexual, not a predator deterrent : I am unaware of any evidence suggesting that these male stridulatory devices were originally found in both sexes and then lost in the females. Note : the few cases of female stridulation in the Ensifera involve structures that are not homologous with the male file and scraper.

Our new discussion makes this point clear. We do not have enough neuroanatomical data to explicitly show that internal hearing sensory organs were ancestral, but it is not impossible. What we can tell is that external tympana probably evolved multiple times. We also make it clear that the evolution of tegmino-tegminal stridulation repeatedly occurred in crown-Orthoptera and that the common ancestor of Ensifera evolved a male-specific stridulatory apparatus probably in the context of sexual communication.

***

General point : Given that the main objective of this paper is evolution of acoustic communication in the Orthoptera, it would be helpful to present the general theories on the evolution of sexual signaling : 1) Signals evolve via ‘sensory bias’ wherein one sex, generally the male, develops signals that the other sex, generally the female, already has perception for and in a general, non-sexual context. 2) Signals (in males) and perception and preference (in females) co-evolve with each other from their origin ; the so-called Fisherian mechanism. Can you infer which mechanism(s) were likely responsible for the evolution of acoustic communication in the several orthopteran lineages ? See reference 10.

We have revised our introduction to reflect this suggestion and presented these two hypotheses up front. Throughout the discussion, we comment on whether an observed

pattern fits with either hypothesis.

Line 294 ‘peculiar position’ - explain

We replaced the word ‘peculiar’ with ‘specific’ and explained our hypothesis for the evolution of directional hearing early in the diversification of Ensifera in the context of sexual communication.

Lines 294-296 Your suggestion here reflects my comment on lines 252-257 ; hearing and stridulation coevolved early, and all were functioning in a sexual, rather than generalized, context.

Yes it does, and we have revised our text to reflect this.

Line 304 Again, more evidence favoring early coevolution.

We have revised our text to reflect this.

Line 329 ‘comes’

Corrected.

Line 336 ‘show’

Corrected.

Line 379 Regarding the connection between flight and hearing in insects, it is interesting to note that hearing is only found in groups that fly or are secondarily flightless (e.g. brachypterous tettigoniids). However, some very aerial groups have little or no hearing. One might infer that flight is a necessary precursor because the air chamber in tympanal ears is derived from the tracheal system, and aerial insects will have more developed trachea to support their higher oxygen demands. But devoting a major portion of the tracheal system to perception rather than supplying oxygen may not have been economically sustainable in the most aerial insects that fly very often and with high wingbeat rhythms (e.g. Hymenoptera, Diptera, Odonata). Additionally, those groups that truly excel in flight have, by necessity, very well developed eyes, and the evolution of a second main perception – hearing – may not have been necessary – save in nocturnally active species – and might have detracted from visual ability. See Greenfield, M. D. (2002). Signalers and receivers: Mechanisms and evolution of arthropod communication. New York: Oxford University Press ; page 223 and pages 219-229 in general).

Thank you for sharing this thought. These are important concepts that will need to be tested at a much larger context across the insects. What we present in the paper are the particular patterns we observe in Orthoptera, in which, the secondary loss of wings is correlated with the secondary loss of hearing. If we consider that the original function

of abdominal tympana was not as a hearing organ, but as an auxiliary organ for flight (modulating flight), then our study does make sense.

Line 412 - While it might be said that sexual selection is a likely factor in speciation (general reference should be given - MJ West Eberhard, e.g.), there is no reason to expect sexual selection to be a greater factor in singing insects than in groups relying on other signaling modalities, eg. Visual display as in butterflies. That is, there is nothing special about acoustic signaling in comparison with other displays.

Our result showing that the evolution of acoustic communication did not affect species diversification rate is consistent with this.

Line 470 'group'

We have removed this sentence as we completely re-wrote the discussion.

Line 471 Origin in an anti-predator context is unlikely given that these stridulatory structures are male and there is no evidence that presence in females was lost secondarily. See comments for lines 252-257.

We have clarified this by suggesting that tegmino-tegminal stridulation evolved repeated in crown-Orthoptera, and the male-specific stridulatory apparatus evolved in the common ancestor of Ensifera.

Figure 1 Having the illustrations on the right side of the figure is very helpful, but it would be much more helpful if the specific figures were connected to specific groups (clades).

Thank you for this suggestion. We have revised to figure to be more clear.

Figure 4 Using Pagel's test is most appropriate, but one wonders how it would reflect the possible joint origin of hearing and singing in Ensifera ; see comments on lines 252-257 and the general point on Fisherian coevolution.

Pagel's test can only show the strength of the correlation, and the possibility of joint evolution is inferred from this correlation. We believe that the revised discussion makes it clear that hearing and sound producing organs within Ensifera could have jointly co-evolved.

Reviewer #1 (Remarks to the Author):

Revision of the manuscript submitted to NATURE COMMUNICATIONS: "Phylogenomic analysis sheds new light on the evolutionary pathways towards acoustic communication in Orthoptera".

This version of the manuscript is thoroughly revised to address the points raised in the previous round of reviewing, and to include new analyses and one new figure. The study itself addresses a fundamental evolutionary problem on orthopteran insects and provides important insights into the relationships of orthopteran insects. It will be essential to open new perspectives on the biology and behaviour of this taxon. To work out the main lines of evolutionary trajectories in the species-rich group requires a selection of investigated species, but the authors are careful in their discussion to point out further possible explanations of their results, or problems resulting e. g. from secondary losses of traits.

The manuscript is now reshaped based on the question of the evolutionary mechanisms of sexual signalling, following the comments from reviewer #3, and this provides a helpful structure for the presentation and discussion of the findings. The points raised previously are incorporated in the revision, and I have only some more discussion points which may add to the clarity of the (admittedly broad) topics covered here:

It might be helpful to keep in mind that the definition of "hearing" can be difficult with respect to the hearing mechanism – usually, tympanal organs clearly enable far-field hearing over a broad frequency range and at high sensitivity, but this on the other hand does not mean that animals lacking tympana are "deaf" (l. 284, 369) or "without the ability to hear" (see l. 186). There are such exceptions as the atympanate hearing organs in bladder grasshoppers that you discuss, and tibial organs may also be sensitive to sound without tympana, as found in cockroaches (Shaw SR, J Exp Biol 193: 13 – 47), which very much supports your reasoning in response to this general problem of the "starting point" of hearing (below). There might even be other mechanisms occuring for hearing, like sensory hairs on the body surface. When covering the tympanal organs and their mechanism in the introduction (l. 79), it may be helpful to mention the main features of tympana for hearing, and change the wording on the lack of hearing.
"l. 253 How would sound generation have been detected then?
Our study lays out a scenario that sound producing organs were ancestral (plesiomorphic) for Ensifera, and tibial tympana evolved multiple times. The ensiferan ears consist of internal hearing sensory organs and external tympana. It is unclear what the original form of sound detection was, but it is conceivable that thin cuticle of foretibia could have functioned as a resonator for the internal sensory organs to pick up the sound wave. We infer that thinning of the cuticle evolved multiple times to give rise to tympanal membrane within Ensifera."

l. 185 the neuroanatomical distinction of dipartite/tripartite sensors: this terminology is clear as it is presented in the text, but it is also somewhat problematic since the accessory organ has been documented recently in some Ensifera as a further sensory organ (e. g. your reference 62). To my reading, the sections with the terminology will also be clear and instructive if the "-partite" references are omitted without necessarily changing the text further.

l. 367 You are right to comment that the homology of the tympana in crickets and mole is not questionable so far, and this would distract too much from the broader discussion of the manuscript – however, the mole crickets have highly modified forelegs, and it would be very interesting to learn in more details about your morphological studies between these two clades at some point.

l. 381 For the katydid input system, this is the standard layout present in most species while the trachea can also expand considerably in the thorax to meet at the midline, potentially allowing for a

coupling between the two body sides: Bailey WJ (1990) The ear of the bushcricket, In Bailey WJ, Rentz DCF (eds) The Tettigoniidae: Biology, Systematics and Evolution, pp 217–247

l. 674 Gryllotalpidae?

l. 787 file

l. 795 Reference 33 is identical to reference 30.

Reviewer #2 (Remarks to the Author):

In this manuscript, the authors estimate a new time-calibrated phylogeny of orthopterans based on transcriptomic data (for ~50 orthopteran species) and mitogenomic data (for an additional ~200 species). They then use this phylogeny to test for correlated evolution of hearing and sound-producing structures. They also test whether these structures are related to variation in diversification rates. I think that the topics are interesting and the paper is relatively data rich. The analyses generally seem reasonable. Overall, the manuscript seems potentially appropriate for Nature Communications.

This is my second time reviewing the manuscript. I think that the authors have made several improvements, which I appreciate (e.g. using HiSSE to directly relate diversification rates and traits). I especially appreciate that the authors have distinguished the Results and Discussion, which now makes it easier to distinguish their results from speculation. However, the writing is still problematic in some places, as explained below.

Note that whether the manuscript is poorly written or not has a great deal to do with the "actual merit of the manuscript." The "actual merit of the manuscript" does relate to the writing, because it is different from "the actual merit of the data" or "the actual merit of the analyses." In the end, why should a major journal publish a poorly written manuscript? And why would the authors want that?

a) Here is one example of problematic writing. Look at lines 168–175. What are these results based on? There is no reference to a figure or table, or citations to other work. The problem arises again on lines 244–263. The authors talk about their results, but there is no reference to any figures or tables containing these results.

Whenever the authors refer to their results, they need to refer to the specific figures, tables, and datasets in which those results are shown. Providing specific support for one's statements is what separates scientific results from speculation.

b) Similarly, every statement that is not from the authors' work should have a supporting citation. For example, on line 213, how do you know that Pagel's test is sensitive to taxon sampling? Where is the citation that supports this?

c) Instead of simply listing their methodological choices, the authors should justify those choices over alternatives. For example, on line 698, why did the authors set the LambdaIsTimeVariblePrior in BAMM to 0, instead of using the default value of 1?

d) The results should be described in an unbiased fashion. For example, on lines 244–263, the authors found that lineages with hearing organs have diversification that are more than twice as high as those without. But they say: "However, the difference between the highest (r = 0.032064) and the lowest (r = 0.013633) diversification rates was not dramatic." What is the exact number required for the difference to be "dramatic", and how is it even relevant? It appears that the authors did a statistical test, came up with a significant difference, and then decided afterwards that the significant difference

was not big enough. That is not really appropriate at all.

Overall, I think that the paper could be publishable in Nature Communications, but the authors should pay more attention to the writing.

The authors say that BAMM “still has merits as it can detect rate shifts and calculate clade-specific diversification rate.” Well, the problem is that (based on simulations) it often does those things incorrectly. No mention is made in the paper of the problems with this method in accurately performing these specific tasks.

Reviewer #3 (Remarks to the Author):

This version of the manuscript is vastly improved. It is now a pleasure to read, as the authors have focused on the evolution of acoustic communication and specified how their thorough phylogenetic analysis provides the necessary framework for evaluating acoustic evolution. The details and analyses of the acoustics add much precision and will be of interest to many readers. I have only two minor points that will sharpen their findings and inferences :

1 Lines 459-466 : The specification that abdominal tympana, as opposed to tympana located elsewhere on the body, may have been involved in flight regulation is a valuable point. Bolstering this inference is that in the Tettigoniidae, two subfamilies (not isolated species or genera), Bradyporinae and Hetrodinae, are extremely brachypterous and flightless, but they retain acoustic communication (hearing and singing). Perhaps tibial tympana are not involved in flight regulation ; additionally, once acoustic communication is well developed, it may not be lost if brachyptery and flightlessness evolve under other selection pressures.

2 Lines 521-531 : Regarding the link between sexual selection and speciation, I do not find the results surprising. First, the theory pertains to sexual selection of any kind, not simply acoustic signaling. If the appearance of acoustic signaling accompanied a loss of other forms of signaling (chemical, visual, substrate vibration), the overall strength of sexual selection may not have changed. Thus, no uptick in speciation occurred. But it is most interesting that the one group in which a relationship was found was the Tettigoniidae : This is the family in which acoustic communicaation appears to be the most widespread and dominant. Unlike Gryllids, and Acridoids, there is little or no evidence for pheromones or visual (sexual) signaling in Tettigoniidae. Perhaps the origin of acoustic communication in this group did represent a major increase in overall sexual selection ?

3 very minor point : Line 376 Sound traveling internally to reach the tympanum also travels over a longer distance than sound reaching the tympanum from the outside.

Michael D Greenfield

RESPONSES TO REVIEWERS' COMMENTS

Reviewer #1 (Remarks to the Author):

Revision of the manuscript submitted to NATURE COMMUNICATIONS: "Phylogenomic analysis sheds new light on the evolutionary pathways towards acoustic communication in Orthoptera".

This version of the manuscript is thoroughly revised to address the points raised in the previous round of reviewing, and to include new analyses and one new figure. The study itself addresses a fundamental evolutionary problem on orthopteran insects and provides important insights into the relationships of orthopteran insects. It will be essential to open new perspectives on the biology and behaviour of this taxon. To work out the main lines of evolutionary trajectories in the species-rich group requires a selection of investigated species, but the authors are careful in their discussion to point out further possible explanations of their results, or problems resulting e. g. from secondary losses of traits.

Thank you for your kind words.

The manuscript is now reshaped based on the question of the evolutionary mechanisms of sexual signalling, following the comments from reviewer #3, and this provides a helpful structure for the presentation and discussion of the findings. The points raised previously are incorporated in the revision, and I have only some more discussion points which may add to the clarity of the (admittedly broad) topics covered here:

It might be helpful to keep in mind that the definition of "hearing" can be difficult with respect to the hearing mechanism – usually, tympanal organs clearly enable far-field hearing over a broad frequency range and at high sensitivity, but this on the other hand does not mean that animals lacking tympana are "deaf" (l. 284, 369) or "without the ability to hear" (see l. 186). There are such exceptions as the atympanate hearing organs in bladder grasshoppers that you discuss, and tibial organs may also be sensitive to sound without tympana, as found in cockroaches (Shaw SR, J Exp Biol 193: 13 – 47), which very much supports your reasoning in response to this general problem of the "starting point" of hearing (below). There might even be other mechanisms occuring for hearing, like sensory hairs on the body surface. When covering the tympanal organs and their mechanism in the introduction (l. 79), it may be helpful to mention the main features of tympana for hearing, and change the wording on the lack of hearing.

Thanks for this suggestion. Indeed, it is true that the lack of tympanal ears does not necessarily mean that the species is deaf or has no ability to hear. We have clarified this point in the introduction and made it clear that our paper mostly concerns far-field hearing. We have also revised those places where we initially referred to atympanate ensiferans as being deaf, and correctly suggested that they are unable to hear far-field.

“l. 253 How would sound generation have been detected then?
Our study lays out a scenario that sound producing organs were ancestral (plesiomorphic) for Ensifera, and tibial tympana evolved multiple times. The ensiferan ears consist of internal hearing sensory organs and external tympana. It is unclear what the original form of sound detection was, but it is conceivable that thin cuticle of foretibia could have functioned as a resonator for the internal sensory organs to pick up the sound wave. We infer that thinning of the cuticle evolved multiple times to give rise to tympanal membrane within Ensifera.”

Thanks for this suggestion. We have revised the section where we talk about the evolution of complex tibial organs, and incorporated the suggested wordings.

l. 185 the neuroanatomical distinction of dipartite/tripartite sensors: this terminology is clear as it is presented in the text, but it is also somewhat problematic since the accessory organ has been documented recently in some Ensifera as a further sensory organ (e. g. your reference 62). To my reading, the sections with the terminology will also be clear and instructive if the “-partite” references are omitted without necessarily changing the text further.

Thanks for this suggestion. We agree with the suggestion and removed all the references to bipartite/tripartite sensors.

l. 367 You are right to comment that the homology of the tympana in crickets and mole is not questionable so far, and this would distract too much from the broader discussion of the manuscript – however, the mole crickets have highly modified forelegs, and it would be very interesting to learn in more details about your morphological studies between these two clades at some point.

Thanks for this comment. In the interest of keeping the argument focused, we did not change any wording regarding the mole crickets.

l. 381 For the katydid input system, this is the standard layout present in most species while the trachea can also expand considerably in the thorax to meet at the midline, potentially allowing for a coupling between the two body sides: Bailey WJ (1990) The ear of the bushcricket, In Bailey WJ, Rentz DCF (eds) The Tettigoniidae: Biology, Systematics and Evolution, pp 217–247

Thanks for this suggestion. We have added a sentence about acoustic bullae in katydids. Initially we did not include this for conciseness, but we think that addition of this sentence makes the argument comprehensive.

l. 674 Gryllotalpidae?

Thanks for catching this typo. We have corrected it.

l. 787 file

Thanks for catching this mistake. It has been corrected.

l. 795 Reference 33 is identical to reference 30.

Thanks for catching this mistake. We have removed the duplicate reference.

Reviewer #2 (Remarks to the Author):

In this manuscript, the authors estimate a new time-calibrated phylogeny of orthopterans based on transcriptomic data (for ~50 orthopteran species) and mitogenomic data (for an additional ~200 species). They then use this phylogeny to test for correlated evolution of hearing and sound-producing structures. They also test whether these structures are related to variation in diversification rates. I think that the topics are interesting and the paper is relatively data rich. The analyses generally seem reasonable. Overall, the manuscript seems potentially appropriate for Nature Communications.

This is my second time reviewing the manuscript. I think that the authors have made several improvements, which I appreciate (e.g. using HiSSE to directly relate diversification rates and traits). I especially appreciate that the authors have distinguished the Results and Discussion, which now makes it easier to distinguish their results from speculation.

Thanks for your comments and we are encouraged to learn that you appreciate our efforts.

However, the writing is still problematic in some places, as explained below.

Note that whether the manuscript is poorly written or not has a great deal to do with the "actual merit of the manuscript." The "actual merit of the manuscript" does relate to the writing, because it is different from "the actual merit of the data" or "the actual merit of the analyses." In the end, why should a major journal publish a poorly written manuscript? And why would the authors want that?

We understand the points you raised. We have tried to improve the quality of our writing in this revision.

a) Here is one example of problematic writing. Look at lines 168–175. What are these results based on? There is no reference to a figure or table, or citations to other work. The problem arises again on lines 244–263. The authors talk about their results, but there is no reference to any figures or tables containing these results.

We have gone through the manuscript and inserted figure references or appropriate citations where they are necessary.

Whenever the authors refer to their results, they need to refer to the specific figures, tables, and datasets in which those results are shown. Providing specific support for one's statements is what separates scientific results from speculation.

We have now added specific figure numbers and references whenever we refer to our results.

b) Similarly, every statement that is not from the authors' work should have a supporting citation. For example, on line 213, how do you know that Pagel's test is sensitive to taxon sampling? Where is the citation that supports this?

Thanks for this suggestion. We have gone through the manuscript and made sure every statement that is not from our work has a proper citation. We have also included a citation for the issue of taxon sampling in correlation analyses.

c) Instead of simply listing their methodological choices, the authors should justify those choices over alternatives. For example, on line 698, why did the authors set the LambdaIsTimeVariblePrior in BAMM to 0, instead of using the default value of 1?

We believe that we have sufficiently provided enough rationale for our methodological choices, which can be replicated. The one point the reviewer asked about regarding the BAMM parameter, we have provided an appropriate explanation in the text. This parameter was selected to ensure that the analysis will achieve convergence, following the program's manual.

d) The results should be described in an unbiased fashion. For example, on lines 244–263, the authors found that lineages with hearing organs have diversification that are more than twice as high as those without. But they say: "However, the difference between the highest (r = 0.032064) and the lowest (r = 0.013633) diversification rates was not dramatic." What is the exact number required for the difference to be "dramatic", and how is it even relevant? It appears that the authors did a statistical test, came up with a significant difference, and then decided afterwards that the significant difference was not big enough. That is not really appropriate at all.

We have removed that sentence because it does represent our bias. It does not change anything with this removal and thanks for this suggestion.

Overall, I think that the paper could be publishable in Nature Communications, but the authors should pay more attention to the writing.

The authors say that BAMM "still has merits as it can detect rate shifts and calculate clade-specific diversification rate." Well, the problem is that (based on simulations) it often does those things incorrectly. No mention is made in the paper of the problems

with this method in accurately performing these specific tasks.

We have included two citations, one outlining the criticisms of BAMM and one rebutting the criticisms. We acknowledge the controversy and we try not to over-interpret the result.

Reviewer #3 (Remarks to the Author):

This version of the manuscript is vastly improved. It is now a pleasure to read, as the authors have focused on the evolution of acoustic communication and specified how their thorough phylogenetic analysis provides the necessary framework for evaluating acoustic evolution. The details and analyses of the acoustics add much precision and will be of interest to many readers.

Thank you for this kind word.

I have only two minor points that will sharpen their findings and inferences :

1 Lines 459-466 : The specification that abdominal tympana, as opposed to tympana located elsewhere on the body, may have been involved in flight regulation is a valuable point. Bolstering this inference is that in the Tettigoniidae, two subfamilies (not isolated species or genera), Bradyporinae and Hetrodinae, are extremely brachypterous and flightless, but they retain acoustic communication (hearing and singing). Perhaps tibial tympana are not involved in flight regulation ; additionally, once acoustic communication is well developed, it may not be lost if brachyptery and flightlessness evolve under other selection pressures.

Thanks for raising this point. We have added a sentence at the end of the paragraph suggesting, the retention of the hearing ability in brachypterous katydids and crickets provides an indirect support of our argument.

2 Lines 521-531 : Regarding the link between sexual selection and speciation, I do not find the results surprising. First, the theory pertains to sexual selection of any kind, not simply acoustic signaling. If the appearance of acoustic signaling accompanied a loss of other forms of signaling (chemical, visual, substrate vibration), the overall strength of sexual selection may not have changed. Thus, no uptick in speciation occurred. But it is most interesting that the one group in which a relationship was found was the Tettigoniidae : This is the family in which acoustic communicaation appears to be the most widespread and dominant. Unlike Gryllids, and Acridoids, there is little or no evidence for pheromones or visual (sexual) signaling in Tettigoniidae. Perhaps the origin of acoustic communication in this group did represent a major increase in overall sexual selection ?

This is a valid point. Especially, we appreciate the suggestion about katydids being special, and we have included a sentence suggesting a possibility of the evolution of

acoustic signalling increasing the diversification rate as it is the most widespread and dominant form of communication in this lineage.

3 very minor point : Line 376 Sound traveling internally to reach the tympanum also travels over a longer distance than sound reaching the tympanum from the outside.

Thanks for this point. We have clarified this sentence.

Michael D Greenfield

REVIEWERS' COMMENTS:

Reviewer #1 (Remarks to the Author):

This phylogenetic analysis of Orthoptera is based on the transcriptomic data from 50 species and the mitochondrial genomes from ~250 species in the largest data set gathered so far for the taxon. This revised version of the manuscript is corrected for the points raised previously and is now more concise. The manuscript does not dwell on the differences and similarities to most previous studies, but shows in the resulting phylogeny the relevance of large data sets to resolve deep branching patterns, and provides the scaffold for the insights on the evolution of acoustic communication in the different lineages. The strength is the approach with multiple representatives for the majority of clades and the calibration by fossils.

Overall, it is now acceptable for publication.
For this manuscript version, I have only few (very minor) remarks for edits on the text:

l. 41 This sentence should be checked – should it be "…in the suborder Caelifera, abdominal tympanal ears…" or "…in the suborder Caelifera, the abdominal tympanal ear…"

l. 687 …using the Akaike Information Criterion (AIC)

l. 867 "fawed" – should be "flawed"

Check the references, as some for some references the journal names are not abbreviated, see
l. 868, 961, 971, 1004

l. 912 missing the title of the reference?

l. 1043 should read "The names in white, in red or blue bars…" (with additional comma after "white")

Reviewer #2 (Remarks to the Author):

I am reasonably satisfied that the authors have done their best to address my previous set of concerns (and those of the other reviewers). Therefore, I am happy to recommend that the paper be accepted now. Congratulations to the authors on an interesting and data-rich study!

Reviewer #3 (Remarks to the Author):

I am satisfied with the latest revision of the manuscript.

RESPONSES TO REVIEWERS' COMMENTS

Our responses in red.

Reviewer #1 (Remarks to the Author):

This phylogenetic analysis of Orthoptera is based on the transcriptomic data from 50 species and the mitochondrial genomes from ~250 species in the largest data set gathered so far for the taxon. This revised version of the manuscript is corrected for the points raised previously and is now more concise. The manuscript does not dwell on the differences and similarities to most previous studies, but shows in the resulting phylogeny the relevance of large data sets to resolve deep branching patterns, and provides the scaffold for the insights on the evolution of acoustic communication in the different lineages. The strength is the approach with multiple representatives for the majority of clades and the calibration by fossils.

Overall, it is now acceptable for publication.

Thank you so much for your positive review.

For this manuscript version, I have only few (very minor) remarks for edits on the text:

l. 41 This sentence should be checked – should it be "…in the suborder Caelifera, abdominal tympanal ears…" or "…in the suborder Caelifera, the abdominal tympanal ear…"

We can corrected this sentence.

l. 687 …using the Akaike Information Criterion (AIC)

Corrected.

l. 867 "fawed" – should be "flawed"

Corrected.

Check the references, as some for some references the journal names are not abbreviated, see l. 868, 961, 971, 1004

All references have been checked carefully and journal names are now all properly abbreviated.

l. 912 missing the title of the reference?

Corrected.

l. 1043 should read “The names in white, in red or blue bars…” (with additional comma after “white”)

Corrected.

Reviewer #2 (Remarks to the Author):

I am reasonably satisfied that the authors have done their best to address my previous set of concerns (and those of the other reviewers). Therefore, I am happy to recommend that the paper be accepted now. Congratulations to the authors on an interesting and data-rich study!

Thank you so much for your positive review.

Reviewer #3 (Remarks to the Author):

I am satisfied with the latest revision of the manuscript.

Thank you so much for your positive review.
